# Pseudopterosin Biosynthesis: Unravelling a Decades Old Problem in Animal Specialized Metabolism

**DOI:** 10.1021/jacs.4c09925

**Published:** 2025-01-16

**Authors:** Paul D. Scesa, Eric W. Schmidt

**Affiliations:** Department of Medicinal Chemistry, University of Utah, 30 South 2000 East, Salt Lake City, Utah 84112, United States

## Abstract

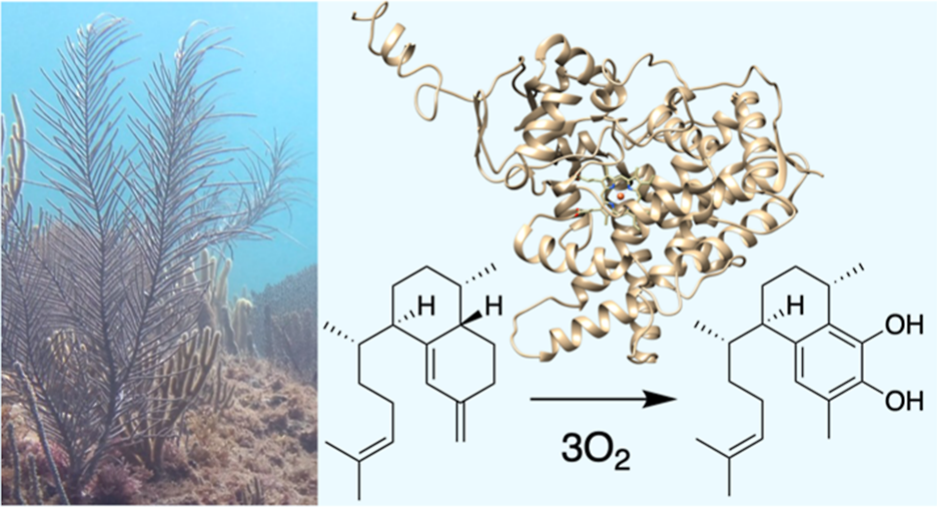

Soft corals are prolific producers of terpenoids, such as pseudopterosins. The exact biosynthetic pathway of these anti-inflammatory diterpene glycosides has eluded the scientific community for decades. Using a forward genetic approach, we have identified, cloned, and expressed the key genes involved in pseudopterosin biosynthesis. We characterized a unique class of multifunctional cytochrome P450 enzymes that catalyze a cascade reaction that produces a nearly mature natural product using a single enzyme. This clarifies the previously proposed biosynthetic pathways to pseudopterosin A and its relatives. The mechanism of the oxidative cascade was probed using in vivo feeding studies in *Saccharomyces cerevisiae* expressing heterologous coral genes. The cascade produces the pseudopterosin aglycone 7,8-dihydroxyerogorgiaene via elisabethatrienol and its epimer, starting from elisabethatriene. This discovery demonstrates the potential to produce this valuable class of natural products using fermentation.

## Introduction

**Figure 1:**
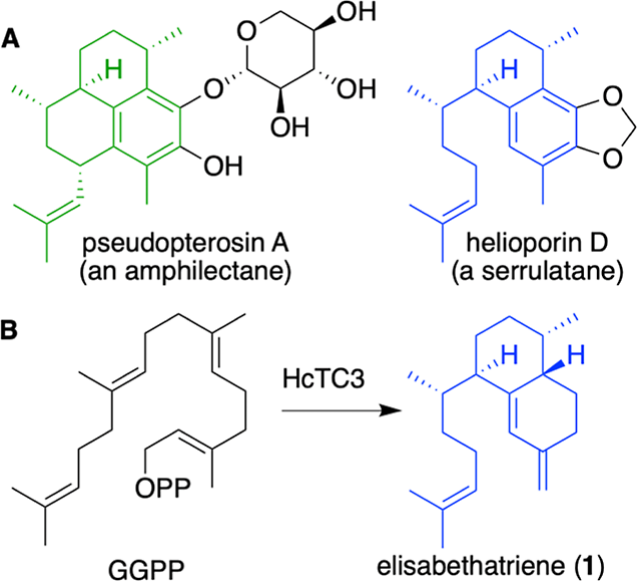
(A) Examples of helioporin and pseudopterosin natural products. The amphilectane core (green) is believed to form in corals from the serrulatane core (blue) after initial terpene cyclization. (B) Formation of **1** is catalyzed by elisabethatriene synthases such as HcTC3.

Octocorals are among the most prolific producers of terpenoids known, with over 2500 reported examples.^1^ Among the most noteworthy coral terpenoids are the pseudopterosin and helioporin classes (Figure 1a).^2,3^ Examples include helioporins A and G from *Heliopora coerulea*, which demonstrate antiviral activity against herpes simplex HSV-1 (IC_50_ = 12.2 μM) and cytotoxicity against murine leukemia P388 (IC_50_ = 6.4 μM), respectively.^3^ On the other hand, first reported in 1985 by the Fenical group, pseudopterosin A from *Antillogorgia elisabethae* is a more potent subcutaneous analgesic in mice than indomethacin (ED_50_ of 3.12 mg/kg vs 10 mg/kg).^4^ Pseudopterosins do not possess opioid agonistic or histamine antagonistic activity and are therefore promising tool molecules for the development of much needed drugs to target chronic pain. Of historical note, pseudopterosin preparations in the form of crude sea whip extracts are one of only two commercially used products derived from octocorals thus far,^5^ the other being prostaglandins.^6^ While their use as topical anti-inflammatories in humans shows tremendous promise, the environmental concerns surrounding the wild harvest of tropical reef octocorals for extraction has stymied further development of pseudopterosins.^7−10^ Although the total chemical synthesis of these compounds has been reported on numerous occasions, this approach has yet to stimulate further industrial development.^11,12^ Synthetic biology as an industrial production platform has shown increasing promise but would rely on extensive elucidation and engineering of the biosynthetic pathways that were previously unknown.

**Scheme 1:**
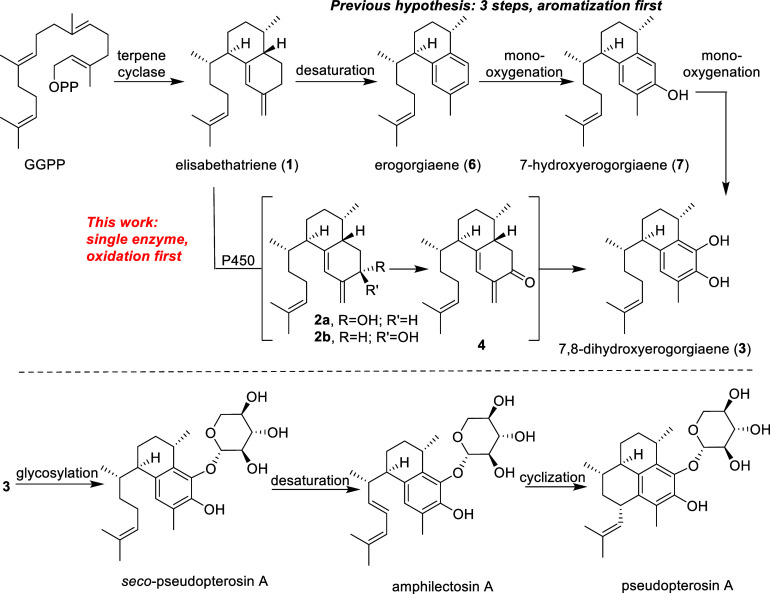
Biosynthetic Hypothesis for the Origin of Pseudopterosin Metabolites

In the early 2000s, Kerr’s group at Florida Atlantic University undertook the ambitious task of determining the biosynthetic pathway to the pseudopterosins using a reverse genetic approach. Determining the exact genes and host organism of pseudopterosin biosynthesis in a prenext generation sequencing world presented a daunting technical challenge. Nonetheless, over many years the Kerr group painstakingly pieced together a biosynthetic hypothesis (Scheme 1) using radiolabeling studies based on incubation with cell-free coral extracts followed by radioactivity-guided and NMR-guided isolation of intermediates.^13,14^ Ultimately, they were able to map out a proposed biosynthetic pathway and, using a radioactivity-assay-guided fractionation of crude coral protein extracts, purify a terpene cyclase to homogeneity. Upon incubation with GGPP, this enzyme produced elisabethatriene (**1**), the first dedicated precursor in the biosynthetic pathway. Unfortunately, proteomic sequencing of this purified protein was never reported, and elucidation of pseudopterosin biosynthesis stalled for nearly two decades.

In 2022, Moore’s group at the Scripps Institution of Oceanography reported the characterization of an elisabethatriene synthase from the coral *H. coerulea* using a forward genetic approach (Figure 1b).^15^ Taking advantage of the abundance of new coral genomes and transcriptomes produced by modern next-generation sequencing, it was shown that octocorals produce terpenoids de novo using genes found in the animal genome.^1,15^ Furthermore, these genes were found to colocalize with other potential biosynthetic genes including cytochrome P450 sequences. Shortly thereafter, the first draft genome of *H. coerulea* and raw transcriptomic data for *A. elisabethae* was published, along with sequencing data for various other presumed elisabethatriene producing corals (see Table S1 for accession numbers).^16^ These data provided a great opportunity to elucidate the pseudopterosin and helioporin biosynthetic pathways using modern approaches. In this work, we describe the draft sequencing and analysis of *H. coerulea*, revealing a colocalization of helioporin biosynthetic genes relative to the known terpene cyclase HcTC3. Phylogenetic analysis and biochemical characterization in yeast (*Saccharomyces cerevisiae*) of the colocalized genes revealed a unique type of multifunctional cytochrome P450s, which produce elisabethatrienol (**2a**) and dihydroxyerogorgiaene (**3**), key precursors in a unified pathway toward the helioporin and pseudopterosin classes of diterpenoids. These genes are conserved throughout both orders of Octocorallia and show promise in the biosynthetic production of these important classes of natural products.

## Results and Discussion

### *H. coerulea* Genome Contains Colocalized Terpene Cyclase and Cytochrome P450 Genes

**Figure 2:**
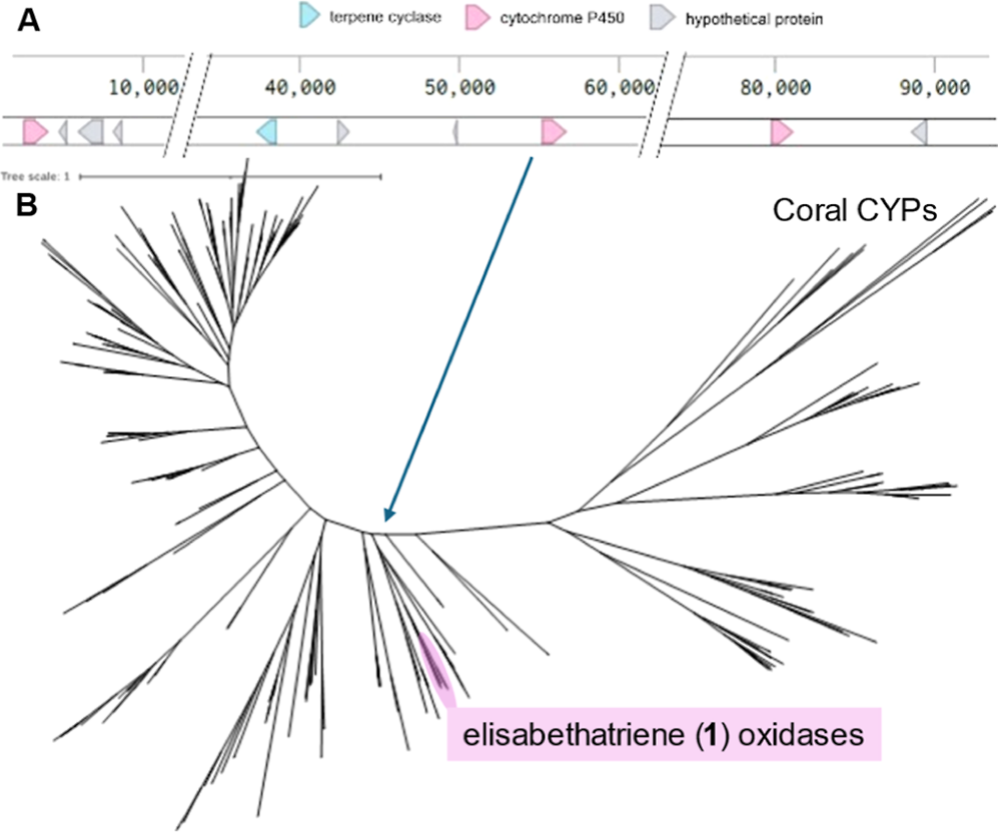
Genomic and phylogenetic analysis of P450 genes. (A) Genome sequencing of *H. coerulea* revealed colocalization of HcTC3 (blue) with a P450 on a coral chromosome (pink). (B) Maximum-likelihood phylogenetic tree of octocoral P450 protein sequences showing a conserved group of putative elisabethatriene (**1**) oxidases (pink), characterized in this work.

The initial report of the elisabethatriene synthase HcTC3, identified from publicly available transcriptomic data, inspired us to use *H. coerulea* as the starting point for elucidating the pseudopterosin and helioporin pathways.^15,17^ The *H. coerulea* transcriptome contains about 60 different cytochrome P450 genes (72 tblastn hits for one published transcriptome and 56 for another), making screening P450 genes biochemically one by one to identify the relevant gene technically challenging. A way to prioritize P450 genes for heterologous expression and characterization was needed. Recently we, concurrently with the Moore group, determined that coral terpene cyclases are present in animal biosynthetic gene clusters.^1,15^ As such, we set out to generate a genome assembly for *H. coerulea*, which was unreported at the time. Using a combination of Illumina short read and Nanopore Minion long reads, we generated a hybrid assembly that showed a 263 kb contig encoding HcTC3 (Figure 2a). Adjacent to HcTC3 was a tandem duplicated cytochrome P450 gene (HcCYP), defining colocalized genes responsible for helioporin biosynthesis. During the course of this work, a draft genome of *H. coerulea* was published (NCBI Bioproject# PRJNA936655).^16^ This high-quality assembly, also generated using long and short reads, showed a similar contig with an identical set of terpene cyclase and P450 genes (GenBank# JASJOG010000029.1). This further supported our hypothesis that a colocalized set of biosynthetic genes was responsible for secondary metabolite production.

### Identification and Characterization of Elisabethatriene Synthases in Pseudopterosin-Producing Octocorals

Having identified a putative elisabethatriene oxidase P450 gene, we questioned whether similar types of genes were conserved in other corals that potentially produce serrulatane or amphilectane diterpenoids. Two elisabethatriene synthases are currently known: HcTC3 and PbTC1 were reported by the Moore group. Phylogenetic analysis of a set of terpene cyclases from publicly available coral transcriptomic and genomic data, as well as an *Antillogorgia acerosa* transcriptome sequenced in house, revealed putative elisabethatriene synthases from diverse corals (Table S1). In addition to those previously identified in *H. coerulea* and *Paramuricea biscaya*, these included two known pseudopterosin producers *Antillogorgia bipinnata* and *A. elisabethae*, and four further octocorals from which amphilectanes or serrulatanes had not been previously reported: *Paramuricea clavata*, *Rhodaniridogorgia sp.*, *Chrysogorgia sp.,* and *A. acerosa*.

The function of four elisabethatriene synthases (Table S2) was confirmed by expression and analysis. The pESC-leu2d vector was used to produce an elisabethatriene synthase and the GGPP synthase (XdCrtE), following our previously reported protocol.^18^ Terpene synthases originated in corals *Rhodaniridogorgia sp.*, *A. bipinnata*, *A. elisabethae*, and *A. acerosa*. When transformed into the YPH499 haploid *S. cerevisiae* strain, the first three terpene synthases produced **1** as detected by TLC and gas chromatography/mass spectrometry (GCMS) analysis (Figure S1). Production was confirmed by preparative scale fermentation, purification of elisabethatriene (**1**), and analysis by NMR (Supporting Information). The terpene cyclase from *A. acerosa* produced a previously unreported isomer of **1**, isoelisabethatriene C (**S1**). The formation of this product supports one of the two proposed cyclization mechanisms for **1** (Scheme S1). Of these coral species, *A. elisabethae* is the commercial pseudopterosin producer.

### Elisabethatriene Oxidase is Conserved throughout All Elisabethatriene Synthase-Containing Corals

Using HcCYP as a query, we used tblastn searches to identify P450 genes in the transcriptomes of the above potential elisabethatriene producers. Phylogenetic analysis of the P450 sequences revealed a clade of P450 genes, which had at least one representative in corals containing genes from the elisabethatriene clade (Figure 2b). This type of P450 gene was not found when the elisabethatriene synthase clade was absent. Based on this information, we inferred HcCYP represents a class of elisabethatriene oxidases conserved throughout both orders of Octocorallia, Scleralcyonacea, and Malacalcyonacea. This indicates that an ancient secondary metabolic pathway was conserved in these animals.

**Figure 3:**
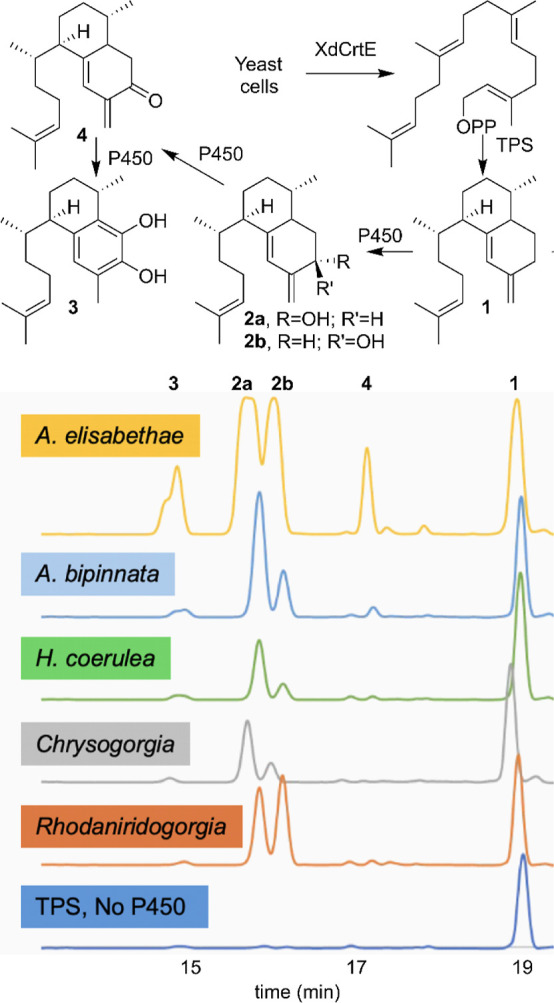
HPLC analysis of crude extracts of yeast harboring terpene cyclase/synthase (TPS) and GGPP synthase XdCrtE genes coexpressed with cytochrome P450 genes. Analytes are detected at λ = 240 nm by DAD. Diterpene hydrocarbon substrate and product peaks are annotated using compound numbers. These genes were identified from diverse coral species, indicated by color.

**Scheme 2:**
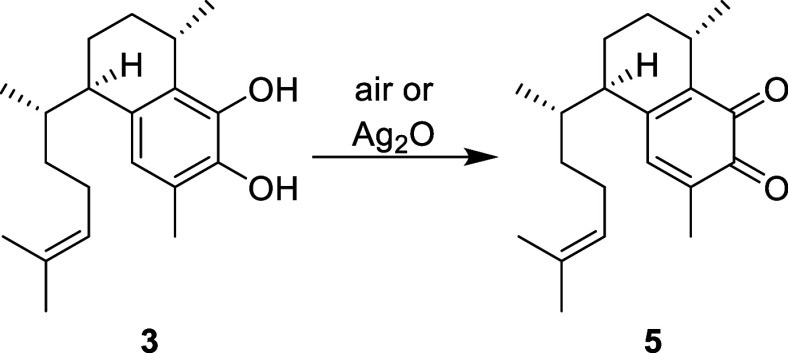
Oxidation of **3** to **5**

To confirm the involvement of these unique coral P450 genes, we cloned and coexpressed five of them (Table S2) with three different elisabethatriene synthases (Figures 3, S2–S5). The P450 genes were inserted into the pESC-URA vector backbone, along with the *H. coerulea* cytochrome P450 reductase (CPR). This CPR was readily identified, as coral transcriptomes usually contain only one CPR encoded by the animal genome, while many different dinoflagellate-derived CPR genes can also be detected. Plasmids encoding both the HcCPR and putative elisabethatriene oxidases were transformed into the YPH500 haploid *S. cerevisiae* strain. Mating between the terpene-producing YPH499 derivative strains and YPH500 strains harboring coral P450 genes produced a set of diploid strains that would produce diterpenes while expressing the coral P450 in an adequate redox environment. Growth of these strains in synthetic complete media with galactose induction for 4 days at 20 °C with shaking produced a mixture of products that could be detected by UPLC-HRESIMS. These products were not present in a negative control expressing the elisabethatriene synthase, GGPPS, and HcCPR but without P450. Scaled up cultures grown in a 12 L fermenter allowed purification of compounds **2a**, **2b**, and **3**, the structures of which were determined by NMR analysis (Figure S6 and Supporting Information spectra).^19−21^ In addition, the ketone intermediate (**4**) was identified by UPLC-HRESIMS comparison of yeast extracts with a semisynthetic standard of **4** produced by Swern oxidation of a mixture of **2a** and **2b** isolated from the yeast expression experiment.^22^ Compound **2a** has been previously reported as an *A. elisabethae* metabolite. Our 1D ^1^H and ^13^C NMR data for **2a** matched the literature report quite well and confirmed the structure. The epimer **2b** was not previously reported, necessitating structure elucidation with 2D NMR analysis (Figure S6 and Table S3). The instability of **3** presented challenges for purification and structural characterization by NMR. We found that **3** was readily oxidized to the *o*-quinone **5** (Scheme 2), so rapid purification and NMR analysis was needed to limit exposure to air, and long-term storage under inert atmosphere was necessary.^21^ This is consistent with reports by Fenical, where *seco*-pseudopterosins were hydrolyzed to **3** but underwent rapid oxidation to the *o*-quinone. Interestingly, our NMR data for **3** match that reported by Fenical for the corresponding *o*-quinone **5** (this work did not report NMR data for catechol **3**, as it was presumed **3** was completely oxidized in a matter of hours). These inconsistencies led us to perform full structural elucidation of **3** based on 1D ^1^H, 1D ^13^C, and homonuclear as well as heteronuclear 2D NMR spectra and negative mode HRESIMS (Figure S6 and Table S3), thus confirming our proposed structure for **3**. Quantitative conversion of a sample of **3** to **5** (Scheme 2) using silver(II) oxide allowed full spectroscopic characterization of **5** as well.

### Octocorals Use a Multifunctional Cytochrome P450 Enzyme to Catalyze Pseudopterosin Formation

**Figure 4:**
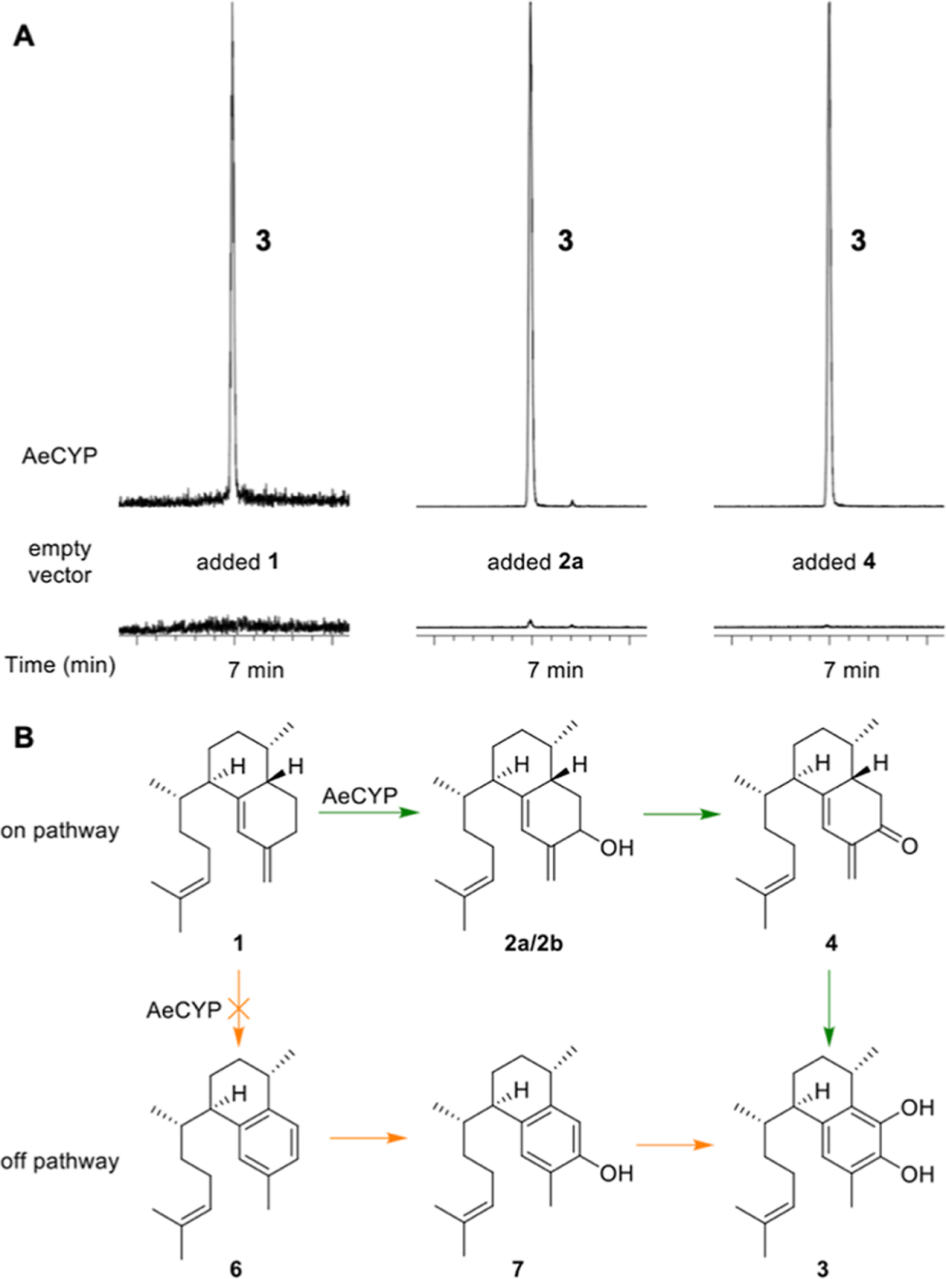
AeCYP is a multistep P450 enzyme (additional data Figures S7–S12). (A) Purified **1**, **2a**, and **4** were fed to AeCYP in *S. cerevisiae*, leading to robust production of **3** only when the enzyme was present, as determined by UPLC-HRESIMS in negative mode. (B) Biosynthesis proposed based on these results (green). By contrast, previously proposed intermediates **6** and **7** are very poor substrates (orange).

**Scheme 3:**
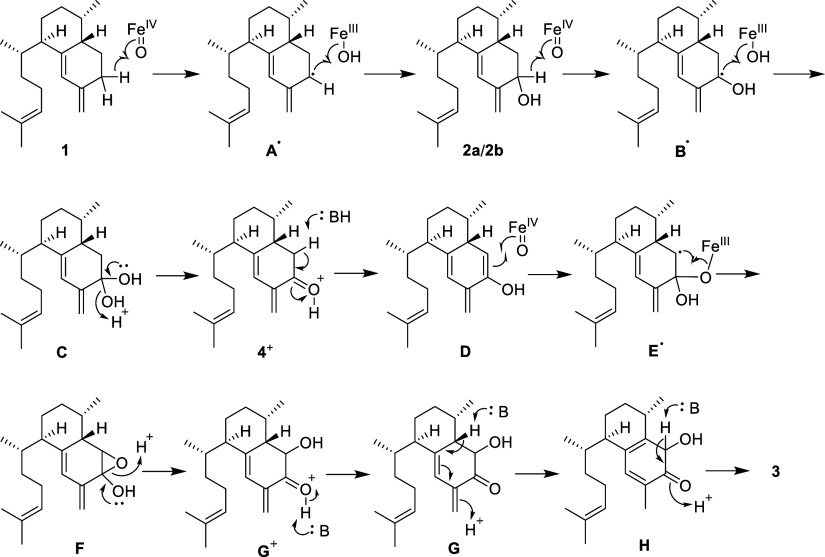
Proposed Mechanism for the Formation of Dihydroxyerogorgiaene

We were inspired to perform a detailed mechanistic analysis of the elisabethatriene oxidases based on feeding studies in yeast using AeCYP from a pseudopterosin producer, ultimately determining a potential enzymatic mechanism and a possible biosynthetic pathway toward the helioporins and pseudopterosins. Fermentation and semisynthesis allowed for facile preparation of proposed biosynthetic intermediates. Compounds **1**, **2a**, **2b**, and **3** could readily be isolated from yeast cultures expressing coral TPS and CYP450 genes. Erogorgiaene (**6**) and 7-hydroxyerogorgiaene (**7**) were produced semisynthetically starting from a mixture of **2a**/**2b** by acid-catalyzed dehydration or PCC-mediated oxidation followed by acid-catalyzed isomerization, respectively. Incubation of **1** with whole yeast cells expressing AeCYP produced **3**, which was detectable by UPLC-HRESIMS (Figure 4a). Although conversion rates were low, this validated our biotransformation conditions as a viable approach to studying the formation of **3**. Subjecting **2a** or **2b** to these conditions also produced detectable quantities of **3** (Figures 4a, S7–S13). Incubation of ketone **4** with the AeCYP expressing yeast strain produced **3** as well. Yeast coexpressing AeTPS and AeCYP did not produce detectable amounts of **6** or **7**. Subjecting semisynthetic **6** and **7** to the biotransformation conditions showed only slow and very minor conversion to **3** (Figures S11, S12). This likely indicates that **6** and **7** are not on pathway and are in fact shunt products, **6** forming by dehydration of **2a** or **2b** and **7** forming by isomerization of **4** (Scheme 1). However, we could not completely rule out other possibilities, such as, **6** and **7** might represent true intermediates under conditions not used in this study. If so, our result might reflect a metabolic network, which has been proposed for some terpenoids as well as other classes of natural products.^23,24^ Based on this information, we propose an alternative hydroxylation-aromatization cascade mediated by a single multifunctional CYP450 (Scheme 3). Initial monooxygenation of **1** occurs via a P450 compound I-mediated radical rebound hydroxylation via radical **A**^**•**^ to afford a mixture of **2a** and **2b**. The unusual stereochemical promiscuity of these multifunctional P450 enzymes then allows for either an alcohol epimer (**2a** or **2b**) to undergo hydrogen atom abstraction and radical rebound at the opposing face of the cyclohexene ring, affording geminal diol **C**, which would rapidly eliminate water to afford a ketone, **4**. This mode of ketone formation mediated by P450 enzymes is exemplified by the albaflavenone biosynthetic pathway.^25^ In any case, a cyclohexadienone could tautomerize (via deprotonation of **4**^**+**^) to the enol **D**, and then P450-mediated radical monooxygenation via **E**^**•**^ would afford epoxide **F**. The latter would undergo acid–base-mediated isomerization via intermediates **G** and **H** to afford **3**. Indeed, an intermediate strikingly similar to **G** has been isolated and characterized during biosynthetic studies of the gossypol pathway (oxidative aromatization of (+)-δ-cadinene).^26^ In contrast to the pseudopterosin pathway in which all three reactions are catalyzed by a single enzyme, in the gossypol pathway separate enzymes perform each reaction in the pathway.^26^ As found in the precedent of the enzyme aromatase,^27^ it is probable that in the synthesis of **3** the substrate would need to leave the enzyme between oxidative steps to allow the P450 reductase to bind and complete the catalytic cycle before another molecule of oxygen binds the heme center. Such an aromatization-dihydroxylation cascade is rare and redefines how we think about animal cytochrome P450 genes. As opposed to the canonical functions such as monooxygenation of xenobiotics or demethylation of steroid hormones, this unique type of P450 enzyme catalyzes the formation of a nearly mature natural product by a single enzyme. Some types of biosynthetic enzymes, such as iterative PKSs, are known for making complex natural product cores as the sole enzymatic protein, including in animals. By contrast, anabolic P450 proteins in animals are usually associated with single oxidative steps such as demethylation of testosterone to produce estrone. The capability of the octocoral P450 to perform multiple steps on multiple carbons profoundly changes our perspective on new types of reactions that animals may use to produce specialized metabolites for purposes such as chemical defense. Possibly, even more highly evolved and dedicated enzymes exist in animals for this purpose. Furthermore, this represents a unique type of biocatalyst to be exploited for the targeted synthesis of the pseudopterosins and helioporins, which are desirable for their potential medicinal activities but still not accessible at scale.

### New Proposal for Pseudopterosin Biosynthesis

The characterization of this enzyme class also allows us to propose a new biosynthetic pathway for the formation of pseudopterosins in coral. Our proposed pathway is as follows (Scheme 1). An animal genome-encoded elisabethatriene synthase (AeTPS) catalyzes the formation of the diterpene serrulatane skeleton from GGPP via a type I cyclization mechanism. Then, a single animal type II CYP450 (AeCYP) catalyzes an aromatization-dihydroxylation cascade to afford the core structure of the *seco*-pseudopterosins, as in **3**. Elisabethatrienol (**2a**) and **4** are intermediates in this cascade, with erogorgiaene (**6**) and quinone (**5**) formed from **3** being shunt products. Additional steps briefly summarized in Scheme 1 would lead to the *seco*-pseudopterosin, pseudopterosin, serrulotane, and amphilectane compounds, while methylation instead of glycosylation would afford the helioporins.

The formation of **3** was particularly intriguing and indicated this class of P450 genes corresponded to multifunctional results leading us to question the biosynthetic pathway toward the pseudopterosins presented in the literature. Previously, the biosynthesis of **3**, a key aglycone precursor to the *seco*-pseudopterosins, was determined by the Kerr group using a combination of radiolabeling studies and consideration of known *A. elisabethae* metabolites reported in the literature.^14^ In 2000, the Kerr group used incubation of ^3^H-GGPP with cell-free coral extracts to identify elisabethatriene (**1**) as the first dedicated precursor of the pseudopterosins.^28^ Indeed, AeTPS does, in fact, produce **1**, supporting this conclusion. In 2001, the Rodriguez group isolated **6** and **7** from the hexane extracts of *A. elisabethae*.^29^ This led Kerr and co-workers to believe that the formation of **3** occurred via successive hydroxylation of **6** to **7** followed by further hydroxylation to **3** (Scheme 1). Feeding ^3^H-GGPP to coral cell free extracts lead to isolation of radiolabeled **1**, **3**, **6**, and **7**.^14^ Feeding these radiolabeled substrates back into coral cell free extracts led to isolation of subsequent metabolic products with radioactivity, supporting their hypothesis. Glycosylation to *seco*-pseudopterosin A, desaturation to amphilectosin A/B, and Friedel-Craft cyclization to pseudopterosin A and diversification by late-stage acetylation of the sugar moiety accounted for the diversity of the pseudopterosin class.^13,30^

Again, each of these steps was supported by the respective radiolabeling studies. In 2006, Fujimoto and co-workers described the isolation and characterization of **2a**, which was believed to be a direct monooxygenase precursor to erogorgiaene (**6**) via dehydration.^19^ This overall pathway is summarized in Scheme 1. While careful spectroscopic characterization verified the presence of each of these molecules in *A. elisabethae*, the metabolic relationship between each molecule was inferred solely from radiolabeling studies, a method that has since been called into question.^31^ In an era when sequencing of animal genomes was in its infancy and very few genetic tools existed for corals, no genetic evidence was used to support these claims. Classical biochemical purification and characterization of the enzyme involved in each step presented considerable technical challenges, and only the terpene cyclase was enzymologically characterized, despite the lack of sequence or purity information.^7^ The overall biosynthetic proposal was further convoluted by the assertion that the genes responsible for this pathway were encoded in the symbiotic dinoflagellate genome, a hypothesis challenged by numerous bodies of counterevidence.^1,15,31,32^ Overall, the detailed biochemical characterization of the P450 oxidative cascade described herein provides a biosynthetic proposal that accounts for all data.

Another interesting aspect of this work is that some of these corals have yet unknown natural product chemistry, such as *Rhodaniridogorgia* and *Chrysogorgia* from the deep ocean. On the other hand, *A. acerosa* has very well-studied chemistry but has been found to produce cembrene-derived diterpenoids exclusively. Although not metabolically apparent, *A. acerosa* seemingly has genetic potential to produce pseudopterosins. This would indicate that, like in bacteria and fungi, some secondary metabolite genes may be “silent” or not expressed to a significant extent. This opens the door for the possibility that animals, including soft corals, could have cryptic biosynthetic pathways and genetic approaches as described here will allow access to new genetic potential.

## Conclusions

We characterized a class of multifunctional cytochrome P450 genes present in soft coral genomes. The genes encode the catechol fragment of the pseudopterosin and helioporin classes of natural products using an enzyme that catalyzes a cascade of oxidative reactions leading to an advanced intermediate from a simple hydrocarbon. These results have allowed us to unequivocally demonstrate a biosynthetic pathway for the *seco*-pseudopterosins, demonstrating that a single multifunctional P450 alone is sufficient to catalyze conversion of **1** to **3**. Such an enzyme would have significant biotechnological utility, opening the door to producing these valuable natural products through heterologous expression and microbial fermentation. Given the illustrious history of the field using very challenging experiments with radioisotopes,^31^ this study also provides precise molecular tools for future work reinvestigating the biosynthesis in the corals themselves. Perhaps these experiments will reinforce the previous acquired knowledge or may open unimagined possibilities in biochemistry and chemical ecology.

## Supporting Information Available

Detailed experimental procedures, NMR spectra, and protein sequences (PDF)

## Abbreviations

TC: terpene cyclase
CYP450: cytochrome P450
TLC: thin-layer chromatography.
