# Supplementary material for: Pseudopterosin Biosynthesis: Unravelling a Decades Old Problem in Animal Specialized Metabolism
Source: J Am Chem Soc. 2025 Jan 16;147(4):3072–9. doi: 10.1021/jacs.4c09925 (PMC11784711; doi:10.1021/jacs.4c09925)
Supplement: Supplementary file 1 — ja4c09925_si_001.pdf [file ja4c09925_si_001.pdf]

Supporting Information

Pseudopterosin biosynthesis: Unravelling a decades old problem in animal specialized metabolism

Paul D. Scesa and Eric W. Schmidt *

Department of Medicinal Chemistry, University of Utah, 30 South 2000 East, Salt Lake City, UT 84112 (USA)

Table of Contents

I. Sequencing and bioinformatic analysis 3

A. H. coerulea *DNA extraction and sequencing* 3

B. RNA extraction and sequencing 3

C. Phylogenetic analysis of CYP genes 3

II. Biochemical screening, preparative scale fermentation and structure elucidation 4

A. General chemical procedures and instrumentation 4

B. Strains and plasmids 4

C. Validation of terpene cyclases in yeast 7

D. Biochemical screen of CYP genes by co-expression 9

E. Preparative scale fermentation and compound purification 9

*F. Oxidation of **3** for purification and characterization of **5*** 10

*G. Structure elucidation of epi-elisabethatrienol (**2b**)* 11

III. Semisynthesis of proposed biosynthetic intermediates and feeding studies in yeast 18

A. Semisynthesis of proposed biosynthetic intermediates 18

B. Feeding studies in yeast 19

IV. Selected NMR spectra 28

I. Sequencing and bioinformatic analysis

Table S1. NCBI accession numbers for SRA sequencing data

entry	Accession #	species	Type	reference
1	ERX4592531	*Antillogorgia elisabethae*	transcriptome	unpublished
2	ERX4592532	*Antillogorgia elisabethae*	transcriptome	unpublished
3	ERX4592533	*Antillogorgia elisabethae*	transcriptome	unpublished
4	ERX4592534	*Antillogorgia elisabethae*	transcriptome	unpublished
5	ERX4592535	*Antillogorgia bipinnata*	transcriptome	unpublished
6	ERX4592536	*Antillogorgia bipinnata*	transcriptome	unpublished
7	ERX4592537	*Antillogorgia bipinnata*	transcriptome	unpublished
8	ERX4751451	*Antillogorgia bipinnata*	transcriptome	unpublished
9	ERS3886405	*Paramuricea clavata*	genome	Ledoux et al
10	SRS16816628	*Heliopora coerulea*	genome	Ip et al
11	SRR15421816	*Rhodaniridogorgia* sp.	transcriptome	unpublished
12	SRR25705989	*Chrysogorgia* sp.	transcriptome	unpublished

A. H. coerulea *DNA extraction and sequencing*

A live sample of *H. coerulea* was purchased from LivaAquaria.com. A portion of tissue was frozen in liquid nitrogen and ground with a mortar and pestle. The DNA was extracted by incubating in CTAB buffer (20 ml) containing proteinase K for 1 hour at 50 °C. The suspension was centrifuged, extracted with chloroform-phenol (20 ml), washed with chloroform-isoamyl alcohol (10 ml) and the DNA precipitated with isopropanol (20 ml). The precipitate was centrifuged, washed with 75% ethanol, air dried and dissolved in tris buffer. The crude DNA was treated with RNase for 30 min at 37 °C, then purified on a Zymo DNA clean and concentrate spin column. The DNA was subjected to Nanopore long read and Illumina short read sequencing. A hybrid assembly was generated using MASURCA. Contigs containing TPS genes were identified using BLAST with the reported HcTC-3 sequence as query. These contigs were used for gene prediction with the AUGUSTUS web server. An hmmscan was performed in hmmer3 and the output in table format was used to manually annotate genes on the TPS containing contigs.

B. RNA extraction and sequencing

A live specimen of *A. acerosa* was purchased from KP Aquatics. *A. acerosa* and *H. coerulea* tissue were frozen in liquid nitrogen then ground in a mortar and pestle. In a microcentrifuge tube, the tissue was suspended in 1 ml of Trizol reagent, vortexed briefly, then extracted with 0.5 ml chloroform-isoamyl alcohol and centrifuged. RNA was precipitated using 1 ml of isopropanol then washed with 75% ethanol. The RNA was air dried, then suspended in 3 M lithium chloride solution followed by centrifugation. The RNA pellet was washed with 75% ethanol, air dried and treated with the DNA*free* removal kit (Invitrogen). The RNA was subjected to Illumina sequencing and the reads assembled with SPADES in RNA mode. The *A. bipinnata* and *A. elisabethae* raw RNA sequence read archives (SRA) were downloaded from NCBI and assembled using SPADES in the same manner.

C. Phylogenetic analysis of CYP genes

CYP protein sequences were found by BLAST. The *Antillogorgia* transcriptomes were queried using standalone BLAST, and the *Chrysogorgia* sp., *H. coerulea* and *Rhodaniridogorgia* sp. were found using

the BLAST webserver and downloaded. An initial alignment was made using Geneious and the aligned sequences were trimmed to remove unaligned regions as well as sequences that aligned poorly. These trimmed sequences were used for a second multiple sequence alignment in Geneious, which was then used to generate a maximum-likelihood phylogenetic tree using the IQtree webserver.

II. Biochemical screening, preparative scale fermentation and structure elucidation

A. General chemical procedures and instrumentation

Yields refer to chromatographically and spectroscopically (^{1}H NMR) homogeneous materials, unless otherwise stated. Analytical thin layer chromatography (TLC) was performed on 0.25 mm aluminum backed 60 Å F-254 TLC plates, visualized by UV light (254 nm) and iodine vapor. UV-spectra were obtained on a Nanodrop spectrophotometer using 1 cm glass cuvettes (Thermo Fisher). IR spectra were recorded on a Nicolet iS5 FT-IR spectrometer operating in transmission mode (Thermo Scientific). Optical rotations were recorded on a Model 341 polarimeter (Perkin Elmer). Flash chromatography was performed on a Teledyne ISCO CombiFlash system equipped with a UV detector and autocollector. GCMS was performed on an Agilent 7890B GC equipped with a 5977B MSD and 7693 autosampler and an Agilent DP5 MS +DG column. Analytical HPLC was performed on a Thermo Dionex UltiMate 3000 system equipped with a photodiode array detector. Preparative HPLC was performed on a Hitachi Primaide system equipped with 1110 pump and a 1430 DAD photodiode array detector. High-resolution mass spectra (HR-MS) were obtained using a Waters Acquity UPLC linked to a Waters Xevo G2-XS Q-tof. ^{1}H NMR and ^{13}C NMR spectra were recorded on a Varian iNOVA 500 (1H 500 MHz) NMR spectrometer equipped with a 3 mm Nalorac MDBG probe or Varian iNOVA 600 (1H 600 MHz) NMR spectrometer equipped with a 5 mm Nalorac inverse HCN probe operated using VNMRJ 4.2. Data were processed and analyzed using MestreNova 15.0.0. Chemical shifts were referenced to the solvent residual proton for ^{1}H NMR (δ 7.26 for $CDCl_3$) and the ^{13}C signal for ^{13}C NMR (δ 77.2 for $CDCl_3$).

B. Strains and plasmids

All genes were purchased from Twist Biosciences and cloned in yeast expression vectors with 2µ origin of replication and P_{Gal1}/P_{Gal10} promoters (pESC-URA and pESC-leu2d vector backbones). All inserts included a Kozak sequence (5’-AAAAC<u>ATG</u>-3’) immediately upstream of the start codon (underlined) along with a stop codon at the 3’-end. All protein sequences used are included in Table S2. The design of a plasmid vector for co-expression of coral TPS genes and a geranyl geranyl pyrophosphate synthase (GGPPS) has been described previously.[SI-1] The elisabethatriene synthases from *Antillogorgia acerosa* (AaTPS) *Antillogorgia bipinnata* (AbTPS), *Antillogorgia elisabethae* (AeTPS) and *Rhodaniridogorgia sp.* (RhTPS) were synthesized by Twist Biosciences (codon optimized for *Saccharomyces cerevisiae*) and cloned into the pESC-leu2d vector already containing the GGPP synthase XdCrtE. The TPS containing plasmids were transformed into YPH499 (Agilent) using the Sc. EasyComp Kit (Invitrogen) and grown on agar containing 2% dextrose and synthetic complete media with leucine dropped out (SC -leu) at 30 °C for 3 days. The cytochrome P450 (CYP) genes from *A. acerosa* (AaCYP) *A. bipinnata* (AbCYP), *A. elisabethae* (AeCYP),

[SI-1]Scesa, P. D.; Schmidt, E. W. Biomimetic Approach to Diverse Coral Diterpenes from a Biosynthetic Scaffold. *Angewandte Chemie International Edition* **2023**, *62* (39), e202311406. https://doi.org/10.1002/anie.202311406.

Chrysogorgia sp. (ChCYP), *H. coerulea* (HcCYP) and *Rhodaniridogorgia sp.* (RhCYP) were synthesized by Twist Biosciences (codon optimized for *Saccharomyces cerevisiae*) and cloned into the pESC-URA vector already containing the *H. coerulea* cytochrome P450 reductase (HcCPR). The CYP containing plasmids were transformed into YPH500 (Agilent) as described above and grown on agar containing 2% dextrose and synthetic complete media with uracil dropped out (SC -ura) at 30 °C for 2 days. CYP and TPS genes were combined by yeast mating as follows: Haploid yeast strains (YPH499 with TPS and YPH500 with CYP) were grown overnight in either SC -leu or SC -ura with 2% dextrose at 30 °C, then an aliquot of both a CYP strain and a TPS strain were added to yeast peptone dextrose (YPD) medium and grown for 8 hours at 30 °C. The cells were centrifuged, washed with water then plated onto agar containing 2% dextrose and SC medium with leucine and uracil dropped out (SC -leu/-ura) and grown 3 days at 30 °C. This resulted in a diploid yeast strain which harbored both a TPS and CYP for co-expression. The appropriate empty vector controls were also generated.

Table S2. Protein sequences of enzymes characterized in this study, in FASTA format.

>AaTPS
MACSKQLRVPKQWLKYHDDIFKEPVNSKLFSEDELCVWLKDLDLCHNKSVIAKYVQTTKPYHL
MRHQIVLLPSNALCEKIFTLWTNTIIALFIGDDVLETLNKAEMGEICDAFQLLDEHTHDQFPQIPTI
AEMRQFLMQQKVNEKFIPHVIYFQDFSNNVVKCMLEHGNSSKEDVKDFWRRLVVMIAFYYEGV
EDEVKSSVGVYADDVWKRSLSSAAMVWMIAQEITSEVIGKTTVHAPLLNELYFLGTFYSMVVN
DIYSYKREMLLEASVCNLVQTFTVSKVVPGESEAVQKCVDILNEVVKVMYQKIEKVKQENAGD
QDLCKLFDNIGMATVGWYYFHHYSPRYDDSLWRLPIVPVEDDELKEWRQCTDKDPMEEVMAL
LIKYCEKAKRISDAIIGGKVNMHTNL*
>AbTPS
MSCSKEIYVPRRWVQRHKKIPVLAQPAIEKLISMNELIELVIECGLCDKTSISKMHEKINTYQFMW
CMVDTVPASQYAEEIFKSSLHFLCALFLVDDAVESYSAKEMQDLSRSYDILEQQVCKTFPNFPSIN
EMNESLVHLRKPFDRASITFCMQYVNKITAILLKEGNTPHDVVYNLRRRTSNAISIAFQAVLIKSK
CGSIITSDEMLWRRVFDGLVILFYQFGELISGTTEIAQQHIPVLTELRMLGCLHCIVINDLYSYQRD
KPALSDNIIKTWLLEKSVSSLLEATARCGQILDSIMKYMYQRVEQCKQSNPSCSQLKSLLETTIYT
TVGWIRSHATVVPRYCESQLKVSLVEVEEGEIPKWLAKRDEYGWNVVEKFVETLNDEKHKGIL
NALQGIVDGRDQLLKTQLDMHDDVV*
>AeTPS
MSCSKEIYVPRKWAQRHEKIPVLAQTTIEKLISMNELIELVIECGLCDKTCISKMYERINTYQFMW
CMVDTVPASQYAEELFKISLHFLCALFLVDDAVESYSANEMWDMSRSYDALEQQVCKTYPNFP
SISEMKESLVHLPNPFDRASITFCMQYVNKITAILLKEGNTPNDVVYNLRRRTSNAISIAFQAVLIK
SKCGSTVTSHEMLWRRVFDGLVILFYQFDELISGTTEIAQQHIPVLTELRMLGCLYCIVINDLYSY
QRDKLALSDNIVKTWLLQKSVSSLSEATARCGQILDSIMKYMYQRVEQCKQSNPSCPQLKSLLET
TIFTTVGWIRSHTTVVPRYSESQLKVSLVEVEEEEIPKWLTKRDEYGWNVVETFVENLNDEKHK
GLLDALQGIVDGRDQLLKTQLDMHDDIM*
>RhTPS
MPFSKHIHVPSHWLDGQKAMLNKERDETLISMEKLIDLVTECGLCDEASIRKIDKKINTLQFMWT
LVDTSPSDQWSTEIFINCSHFLCAAFLIDDAVESYSAVEMEKLSNAYDLLEQQSCEAFPNFPSICE
MKKSLEGLMLNVFDIASVTFCMQYVNKIASILLKQGNTRNNLAFNVRRRTSNAISISLQAVLVKS
KHGSKVAPHEMLWRRIFDGLVILFYQFGELTSGVTKHVQQHIAIVTELRMLGCLYCVVLNDLYS
YHRDKFAPSDSIIKTWFTNKTVSSLSEATTKCCQILDAVLKYMYERVEECKLRFPDSPELKVLLES
TVYTTVGWIFSHTIIVPRYAESPLKVILVEVEKLELAAWLAQKDEYGCNVVEEFIKTMNNKENKG
IIDALSGCVEGRGQLVKTLVSDQ*
>AaCYP

MLIEVTATLFTVWILWYIVKIYFERRKMPPGPIPLPIIGNLHQSGMDIPYSMEEICKKYGDLYTVTF
PIGHVVVINSVALMKEALVTKKDDFAGRSSISLYPLDIVLERGDIVSSDFCPALMFRRKVFKTALH
MFGEGKSEAEMRVRDGVIELLDEIEATNGHAFSPKKYIAATIVIVLWKWLVNKKCNYGDPTVDA
LLEFNHKMTSLVAQGSLHQLFPFLRYIPSEFHKTIDKLEDIKKNVFEPELAEHRRSYKECVTRDIT
DSFLFAYEKEQRKSNSKDISNVDVVKCMMVDVILGGADTTSGFFNWFILYMILYPDLQKKLQRE
MDEAVEKNHLPSLQDIPNLHWLQATVCEIIRHASFLPVAMPHSATRNTVLQGYEIRKDTVVFLNL
YRINKDPTVWDEPTEFKPDRFIGADGKFIGWAAFPGYLPFGLGRRNCVGESLAKVQVFIVTSCLL
HRFSFEVVKGEPRPKLEVGPPSSVRNPKEYKCVAKRRM*
>AbCYP
MFVEVLLSLFLIWFISSAARSFIKRRKMPPGPFPLPFIGNVHQLGSDPPFTMDDIRKKYGDIITITTPI
GHVVIVSSGALAREVMVGKKDDFAGRPLYFPAYELLENKDLIAGDYGPLFQFRRQIMLSALHMF
GEGVQTAEEKVNKEVDWLLKEIEDTNERQFVPKKLMMTTTIRVITNWLFCQKYESDDPVLKEL
MDFDEDMLKLNCQCGYYQVLPFLKYFPTDFMKTFTAVRAKIDNFFWSNLKHHETTYKNGVVR
DIMDALIDSYKKEKTKHPHKDIGTIDDLRFLVVDAFLGASDTTSSILSWFLLYMIHYQDVQEEIFK
ELNEIVGRDKLPCLQDIENLPYLRATVCEVMRHSAFAPLSAPHKAIRDSVIEGYHVPKDTILFLNH
WRIHYDSREWDEPTLFKPQRFLEANGDFVGWNTLPGFVPFGFGRRACVGQALGKMQLFIITSRL
LHQYRFEIPEGEPVPAFDGEISAVRYPKEYKLIAKKRF*
>AeCYP
MFIEFSLTLFLIWFISSVARSFMKRRKMPPGPFPLPFIGNVHQLGSNPPFTMDEIRKKYGDVFTITTP
IGDVVVVNSGALAREVMIAKKDDFAGRPLYFPAYELLENKDLIAGDYGPLFQFRRKVMLSALH
MFGEGVQSAEERVNTEVDWLLKKIEDTNERQFIPKKLVMATTIRVITNWLFSQKFETKDPILQEL
MDFDEDMLKLNCQCGYYQVLPFLKYFPTDFMKTFASVRAKIDNFFWSNLKQHQKTYKKGVVR
DIMDALFDSYEKEKTKSPHKDLGTIDDLRFLVVDAFLGTSDTTSSTISWFLLYMIRYLDVQDKIFK
ELNEVVGKDRLPCLEDMENLPYLRATVCEVMRHSVFAPLSAPHKAIRDSMIEGYHVPKDTIVFL
NHWRIHYDPRDWDEPALFKPQRFLEANGNFVGWNTLPAFVPFGFGRRACVGQALAKVQLFIITS
RLLHQYRFEIPDGEPVPPFDGEISAVRYPKEYKLVAKKRF*
>ChCYP
MYAEISILLIVIWFVRSAVVTYIKRRDMPPGPFPLPLLGNIYNIGTEPPFSMDGLRVKYGDVFTVTT
PVGEVVIVSSGALAREALVTKKDDFAGRPQSFPAHELLESKDLLAGDYGPLLQFRRRILISALHLF
GDAMKTVEKRVNREVTWLEDAFEAKNQKAFAPKKYIMMTMISVMSEWLFSERFQFGDAQLEK
LFAFDEDILFLNRQGGYYQLLPFLKYFPTKFMKTFAKVQTTIDTFFSSKLNEHCRTYKDDTVRDIT
DGLLCSFYKEQEKNPTKDLGTVDDLRFLLVDVFIGSSDTSSSIVTWFLLYMIKHENIQEKIAEELE
RVVGRDNLPHYADAENLSFLQATICEVMRHSSFAPFSGPHKAIRDSTINGYHIPKDTMVLFSYWR
IHYDEAEWDEPSVFKPERFINENGKFVGWNAFPGFLPFGVGRRACLGQALAKLQVFIITSRLLHR
FRFKAPEGEIPTYDGETSAVRFPKPYNLVAIERV*
>HcCYP
MFVEILISILLIWLVTYFFQTFKKRQNVPPGPFPFPFIGNLPHLGADPPFTMDKLWKKYGDVYLVK
FPVGMSVIVNSCEAVREALVTRKDDFSGRPIHSSYPFNIITEGRGISSSDYGAQLMFRQKMIGSAV
NVFGNEVKQLEEQVNSATEELLNQVGQMDGQTISLKVYVPAVITSQLWEWLSSRKYHFDDHAL
TTLVEFSEKMKFLLRLGGIFQLLPFLKYLPTKFMKTLEEVLVMRGDIFGSVVEEHRRTYTKGVVR
DVTDCLIAACEAKQTKTVEKNGASVDDIKFLLMDMLFTGADTSTTVVLWFILHILLRKDLQVKL
QQELDAAVGRDRLPLWEDIKNLPYLQATVCEVIRYSTPLPLVPHKTIRDTTIQGYHVPKGTPVFIN
FYRVHLDPKEWDDPTLFKPERFLDANGKFIGWTSVSAFMPFGIGRRECPCQNLAKLQVFSVISCL
LHQFTFEQDCQGLYPIIQETSPGFVNQPMDYKVIARKRS*
>RhCYP
MLAEVVLLSIVIWFIWSAVARFIKRREMPPGPFPLPLLGNIHNIGTDPPFSMDNLRKKYGDVFTVS
TPVGEVVVVNSGVLAREALVTRKDDFAGRPQFFPAHELLESKDLLAGDYGPLLQFRRRLLISALH
LFGEGIRAAEGRINKEVKWLEQDIVATNQKPFSPKKYIMMTMISVISEWLFSERYQFGDAELETLF
AFDEDILFLNRQGSYYQVLPFLKYFPTKYMRTFGKVKTTIDNFFSSQLKTHHATYKDDVIRDITD
AVLCSYYKEKVKNPSKDLGTVDDLRFLLVDVFIGSSDTSSSILTWFLLYMIKYEDTQEKAVRELD
NVVGSDNVPCYRDVENLHFLQSTICEVMRHSVFAPFSAPHKAIRNSTISGYRIPKDTMVLFNFWRI

HYDETEWDEPSIFRPERFLDENGKFVGWNTLPGFLPFGSGRRACLGQALAKLQLFTIASRLLHRF
HFKTPDGEPKPSFDGETSAVRFPKFYKLVAIERK*

C. Validation of terpene cyclases in yeast

Small-scale screen. Diterpene production in vivo was checked in a 24-well plate format. YPH 499 strain containing terpene cyclase plasmids on pESC-leu2d with XdCrtE were seeded into SC -leu media (0.5 ml) and grown overnight at 30 °C. YPG induction media (1.5% w/v yeast extract, 1.5% w/v peptone and 2% w/v galactose, 2 ml) was added to the cultures and the plate shaken at 22 °C and 220 rpm for 4 days. Diethyl ether (2 ml) was added to the cultures, briefly shaken, then the ether layer pipetted off and dried under air. Extracts were dissolved in hexane and checked by GCMS using the following parameters: 75 °C hold for 1 min, an increase to 250 °C after 11 min, an increase to 300 °C after 1 min, and a 1 min hold at 300 °C (14 minutes total) with 1 ml/min of helium carrier gas. A 1:50 split was used.

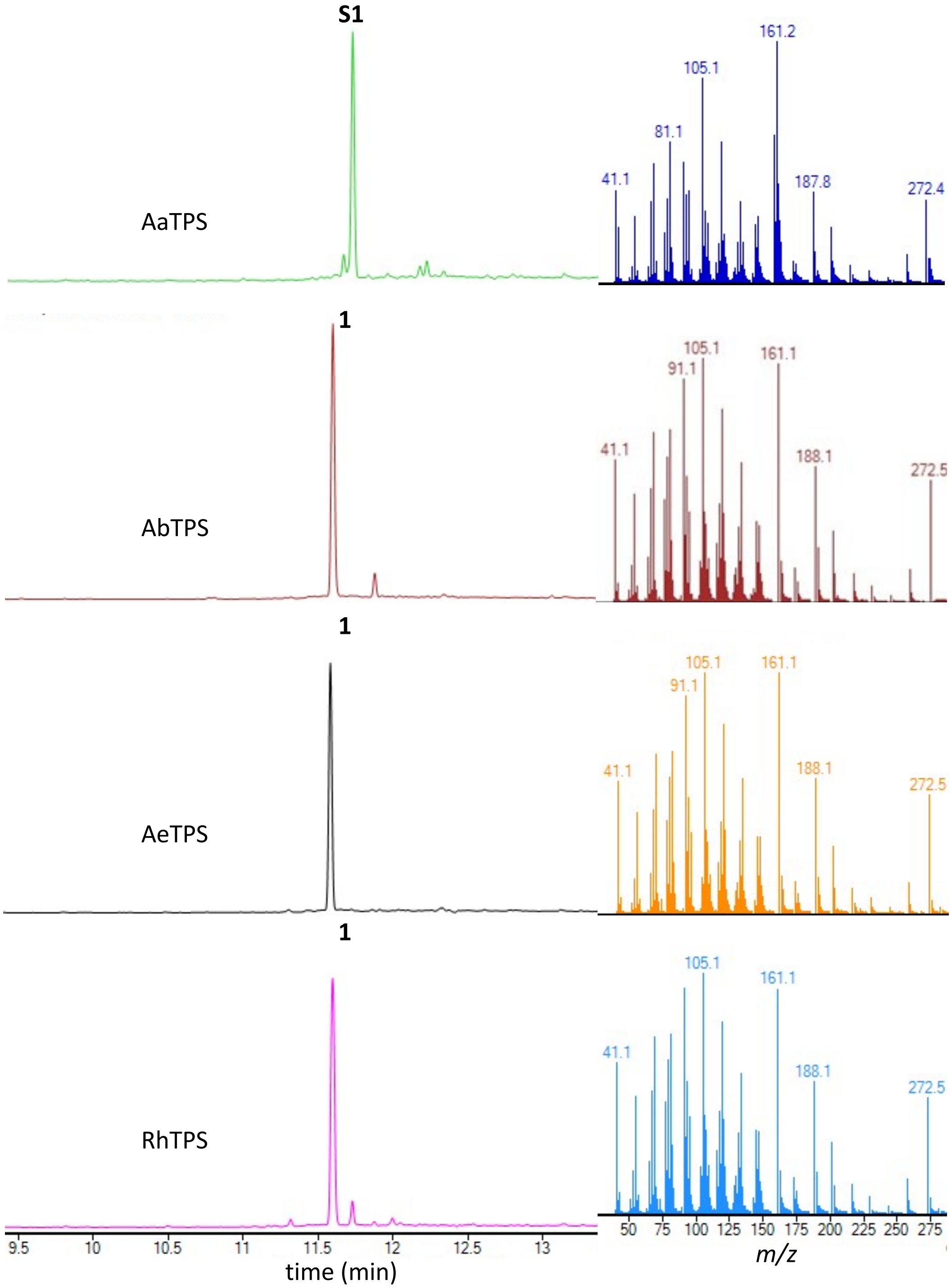

Figure S1. Right) GCMS EIC traces for extracts of yeast harboring terpene cyclases, monitored at 272 *m*/*z*. The peak at 11.6 min is **1** as seen in the trace for AbTPS, AeTPS and RhTPS. In the *A. acerosa* TPS (AaTPS), the peak at 11.7 min is **S1**. Left) EIMS traces for labelled peaks representative of either **1** or **S1**.

Scaled up fermentation to produce diterpenes 1 and S1. YPH 499 strain containing terpene cyclase plasmids on pESC-leu2d with XdCrtE were seeded into SC -leu media (10 ml) and grown overnight at 30 °C. Cells were added to YPG induction media (200 ml) with ethanol sterilized HP20 resin (4 ml) and shaken at 22 °C and 220 rpm for 5 days. The resin was filtered off using cheese cloth, then eluted with acetone (50 ml). The acetone was evaporated under reduced pressure then the residue suspended in water (10 ml) extracted with hexane (10 ml). The hexane layer was evaporated under reduced pressure and the residue purified on a column of silica gel using hexane as mobile phase. RhTPS produced **1** (2.8 mg) and AaTPS produced **S1** (3.1 mg).

Elisabethatriene (**1**): $[\alpha]_D^{20} + 14.2$ (*c* 0.0775, MeOH); $[\alpha]_D^{20} + 65.0$ (*c* 0.325, $CHCl_3$); ^1H NMR (500 MHz, $CDCl_3$) δ 6.03 (s, 1H), 5.08 (t, *J* = 7.0, 1H), 4.71 (s, 1H), 4.66 (s, 1H), 2.39 (m, 2H), 2.20 (m, 1H), 2.02 (m, 2H), 1.83 (m, 4H), 1.74 (m, 3H), 1.68 (s, 3H), 1.61 (s, 3H), 1.37 (m, 4H), 0.94 (d, *J* = 7.0 Hz, 3H), 0.92 (d, *J* = 7.0 Hz, 3H); ^{13}C NMR (125 MHz, CDCl3) δ 144.4, 144.2, 131.0, 126.7, 125.1, 122.0, 108.0, 49.7, 36.9, 35.2, 29.4, 29.3, 27.2, 26.4, 25.7, 25.5, 22.9, 17.7, 17.5, 15.1; HRESIMS *m/z* 273.2592 (calcd for $C_{20}H_{33}^+$, 273.2577). For previous data, see reference SI-2.

Isoelisabethatriene C (S**1**): $[\alpha]_D^{20} - 142.7$ (*c* 0.310, $CHCl_3$); NMR see Table S3.; HRESIMS *m/z* 273.2578 (calcd for $C_{20}H_{33}^+$, 273.2577).

D. Biochemical screen of CYP genes by co-expression

Diploid *S. cerevisiae* strains containing TPS and CYP genes were screened by co-expression in small scale cultures. Yeast strains containing RhTPS in pESC-leu2d and either pESC-URA with AaCYP, AbCYP, AeCYP, ChCYP, HcCYP, RhCYP or pESC-URA with only HcCPR vector (all with XdCrtE and HcCPR) were seeded into SC -leu/-ura (5 ml) with 2% dextrose and grown overnight at 30 °C with shaking at 180 rpm. The seed cultures were used to inoculate SC -leu/-ura medium containing 4 % galactose in a baffled flask. HP20 (2 ml) was added to each culture, then the cells were grown at 20 °C at 220 rpm for 4 days. The suspension was poured into a syringe barrel with a frit on the bottom to collect the resin. The resin was washed with water, then eluted with acetonitrile (3x5 ml). Aliquots of each sample were analyzed by LCMS using the following method: A linear gradient of acetonitrile in water with 0.1 % formic acid was started at 50% acetonitrile and held for 1 min then increased to 100 % acetonitrile after 8 min, then held for 2.5 min at a flow rate of 0.6 ml/min. An Acquity CSH C_{18} column was used (1.7 µm, 2.1x150 mm).

E. Preparative scale fermentation and compound purification

A seed culture (400 ml) of yeast harboring AeCYP and HcCPR on pESC-URA as well as AbTPS and XdCrtE on pESC-leu2d plasmids was grown in SC -leu/-ura with 2% dextrose overnight at 30 °C. This culture was used to inoculate 10 L of SC -leu/-ura medium containing 2% ethanol, 2% glycerol and 0.1 % dextrose in a New Brunswick fermenter set to 500 rpm overhead stirring with aeration. This culture was grown at 20 °C overnight then galactose added to a final concentration of 4%. Amberlite XAD-4 resin (100 ml) was added and the culture was grown for 5 days. The resin was collected and eluted with acetone (2x300 ml) and the solvent evaporated. The residue was partitioned between water and diethyl ether (100 ml each) and the ether layer evaporated. The crude extracted was purified by silica gel flash chromatography using

[SI-2] Kohl, A. C.; Kerr, R. G. Identification and Characterization of the Pseudopterosin Diterpene Cyclase, Elisabethatriene Synthase, from the Marine Gorgonian, Pseudopterogorgia elisabethae. *Archives of Biochemistry and Biophysics* **2004**, *424* (1), 97–104. https://doi.org/10.1016/j.abb.2004.01.019

a gradient of isopropanol in hexane from 0 to 20 %. Fractions were pooled based on TLC to afford elisabethatriene (3.1 mg) and a mixture of other metabolites. The latter was fractionated further by preparative HPLC using a phenylhexyl column and a gradient of 75% acetonitrile in water increasing to 100 % acetonitrile over 25 minutes at 8 ml/min to afford a mixture of elisabethatrienol and *epi*-elisabethatrienol along with pure 7,8-dihydroxyerogorgiaene (26.0 mg). The mixture of epimers was finally purified by semi-preparative HPLC on a C_8 column using a gradient of acetonitrile and water to afford elisabethatrienol (**2a**, 17.2 mg) and *epi*-elisabethatrienol (**2b**, 3.0 mg).

Elisabethatrienol (**2a**): $[\alpha]_D^{20} - 41.7$ (*c* 0.115, MeOH); ^{1}H NMR (500 MHz, $CDCl_3$) δ 5.93 (s, 1H), 5.07 (s, 1H), 5.03 (t, *J* = 7.0 Hz, 1H), 4.80 (s, 1H), 4.20 (ddt, *J* = 12.7, 4.5, 2.2 Hz, 1H), 2.56 (dt, *J* = 11.6, 6.3 Hz, 1H), 2.35 (br s, 1H), 2.03 (ddt, *J* = 14.7, 10.8, 7.3 Hz, 1H), 1.95 (ddt, J = 14.7, 10.8, 7.3 Hz, 1H), 1.91 (dt, *J* = 10.2, 5.3 Hz, 1H), 1.87 – 1.73 (m, 4H), 1.67 (s, 3H), 1.63 (m, 2H), 1.58 (s, 3H), 1.52 (td, *J* = 12.4, 10.8 Hz, 1H), 1.35 (m, 3H), 0.91 (d, *J* = 6.5 Hz, 3H), 0.84 (d, *J* = 7.0 Hz, 3H); ^{13}C NMR (125 MHz, $CDCl_3$) δ 147.5, 143.8, 131.1, 125.6, 124.9, 105.1, 69.6, 47.8, 38.7, 36.3, 34.6, 32.6, 32.4, 28.5, 25.7, 25.5, 21.8, 17.6, 17.5, 14.6; HRESIMS *m/z* 289.2539 (calcd for $C_{20}H_{33}O^+$, 289.2526). For previous data, see reference SI-3.

Epi-elisabethatrienol (**2b**): $[\alpha]_D^{20} + 23.8$ (*c* 0.080, $CHCl_3$); IR (polyethylene film) ν_{max} 3334, 2975, 2917, 2850, 1714 (weak), 1647 (weak), 1462, 1378, 1066 cm^{-1}; NMR see Table S3.; HRESIMS *m/z* 289.2540 (calcd for $C_{20}H_{33}O^+$, 289.2526).

7,8-dihydroxyerogorgiaene (**3**): $[\alpha]_D^{20} - 15.7$ (*c* 0.058, MeOH); UV (MeOH) λ_{max} (log ε) 229 (4.0), 271 (3.4), 332 (2.6); IR (polyethylene film) ν_{max} 2924, 2850, 1710, 1462, 1377, 1288, 1042 cm^{-1}; For NMR data see Table S3; HRESIMS *m/z* 303.2315 (calcd for $C_{20}H_{31}O_2^+$, 303.2319). For previous data, see reference SI-4.

F. Oxidation of 3 for purification and characterization of 5

A solution of **3** (10 mg, 0.033 mmol, 1 eq) in methanol (1 ml) was treated with silver (II) oxide (10 mg, 0.081 mmol, 2.5 eq). The reaction mixture was stirred for 15 min, then filtered through a PTFE syringe filter, evaporated, dissolved in chloroform and filtered through a short bed of silica gel. The solvent was evaporated to provide **5** in quantitative yield.

Quinone **5**: UV (MeOH) λ_{max} (log ε) 222 (3.8), 280 (3.2), 432 (2.3); IR (polyethylene film) ν_{max} 2919, 2850, 1680, 1662, 1647, 1462, 1377, 1259, 1042 cm^{-1}; ^{1}H NMR (500 MHz, $CDCl_3$) δ 6.65 (s, 1H), 5.14 (t, *J* = 7.2 Hz, 1H), 2.87 (m, 1H), 2.29 (m, 1H), 2.04 (m, 2H), 1.95 (s, 3H), 1.73 (s, 3H), 1.69 (m, 3H), 1.65 (s, 3H), 1.51 – 1.38 (m, 2H), 1.08 (d, *J* = 7.0 Hz, 3H), 0.99 – 0.85 (m, 2H), 0.83 (d, *J* = 7.0 Hz, 3H); ^{13}C NMR (125 MHz, $CDCl_3$) δ 184.2, 182.4, 152.9, 143.6, 142.2, 138.4, 134.6, 126.8, 43.8, 38.1, 37.1,

---

[SI-3] Duque, C.; Puyana, M.; Castellanos, L.; Arias, A.; Correa, H.; Osorno, O.; Asai, T.; Hara, N.; Fujimoto, Y. Further Studies on the Constituents of the Gorgonian Octocoral *Pseudopterogorgia elisabethae* Collected in San Andrés and Providencia Islands, Colombian Caribbean: Isolation of a Putative Biosynthetic Intermediate Leading to Erogorgiaene. *Tetrahedron* **2006**, *62* (17), 4205–4213. https://doi.org/10.1016/j.tet.2006.02.032.
[SI-4] Look, S. A.; Fenical, W. The Seco-Pseudopteosins, New Anti-Inflammaytory Diterpene-Glycosides from a Caribbean Gorgonian Octocoral of the Genus *Pseudopterogorgia*. *Tetrahedron* **1987**, *43* (15), 3363–3370. https://doi.org/10.1016/S0040-4020(01)81627-6.

32.4, 29.7, 29.1, 28.7, 28.4, 23.1, 20.5, 19.2, 18.0; HRESIMS *m/z* 323.1995 (calcd for $C_{20}H_{28}O_2Na^+$, 323.1982).

*G. Structure elucidation of epi-elisabethatrienol (**2b**)*

HRESIMS analysis demonstrated an ion at *m/z* 289.2222, indicating an $[M+H]^+$ ion with formula $C_{20}H_{33}O^+$. The overall NMR data was similar to that of **2a**. The ^1H NMR and ^1H-^{13}C HSQC of *epi*-elisabethatrienol (**2b**) showed signals for two aliphatic methyls (δ_H 0.90 and 0.87) and two olefinic methyls (δ_H 1.65 and 1.57). Two olefinic methines (δ_H 5.93 and 5.02), two vinylic methylene protons (δ_H 5.01 and 4.83; δ_C 108.4) and an oxygenated methine (δ_H 4.39; δ_C 68.5) were also observed. The ^{13}C and ^1H-^{13}C HSQC data indicated three double bonds [δ_C 146.5 (C), 144.2 (C), 131.1 (C), 125.0 (CH), 124.0 (CH) and 108.4 (CH_2)], thus necessitating two rings in accordance with the formula. A COSY correlation between H-7 (δ_H 4.39) and an exchangeable proton (δ_H 1.36, EXSY peak with water in the NOESY spectrum) confirmed the presence of an alcohol. The position of this alcohol was inferred by a COSY correlation with H_2-8 (δ_H 1.76) which in turn correlated with H-9 (δ_H 2.55). HMBC correlations from both C-19 protons (δ_H 5.01 and 4.83) to C-5 (δ_C 124.0) and C-7 (δ_C 68.5) along with correlations from H-9 to C-5, C-7 and C-10 (δ_C 144.2) and from H-5 (δ_H 5.93) to C-6 (δ_C 146.5) and C-7 confirmed the presence of the methylenecyclohexenol fragment. The second ring was determined by HMBC correlations, including those from H_3-20 (δ_H 0.87) to C-1 (δ_C 35.0), C-2 (δ_C 29.0) and C-9 and COSY correlations from H-9 to H-1 (δ_H 1.99), H-1 to H-$2_{a/b}$ (δ_H 1.83 and 1.32) and H-2_b to H-3 (δ_H 1.66). These data along with an HMBC correlation from H-5 to C-4 (δ_C 49.4) indicated a fused bicyclic system. Evidence for the C-3 to C-4 connectivity from the COSY spectrum was scarce, as H-2_a and H-4 possessed nearly identical chemical shifts, making the strong COSY cross-peak between δ_H 1.66 and δ_H 1.83 ambiguous. Fortunately, an HMBC correlation from H-3 to C-4 confirmed the presence of the second ring. Once again, signal overlap made COSY analysis ambiguous as H_3-18 and H-12_b showed nearly identical chemical shifts. As such, correlations in the HMBC from H_3-18 (δ_H 0.90) to C-4, C-11 (δ_C 31.6) and C-12 (δ_C 34.7) indicated the branching side-chain position. The final isoprene unit was assigned on the basis of HMBC correlations from H_3-16 and H_3-17 (δ_H 1.65 and 1.57, respectively) to C-14 (δ_C 125.0) and C-15 (δ_C 131.1) along with COSY correlations from H-14 (δ_H 5.02) to H-$13_{a/b}$ (δ_H 2.03 and 1.83) and from H-$13_{a/b}$ to H-$12_{a/b}$ (δ_H 1.34 and 0.91), completing the planar structure of **2b**. The relative configuration was determined by NOESY analysis, as little useful information regarding coupling constants could be found in the highly overlapped ^1H NMR spectrum. Olefinic proton positions were assigned based on NOESY correlations including those between H-14 and H_3-16 and between H-5 and H-19_a as well as H-7 and H-19_b. A NOESY correlation between H-4 and H-5 indicated the *pseudo*-equatorial position of H-4. A NOESY correlation between H-9 and H-11 indicated the *pseudo*-axial orientation of the side chain and the *anti* relationship between H-4 and H-11 in the staggered conformation. While a NOESY correlation was observed between H_2-3 and δ_H 0.91, the similarity between H-12_b and H_3-18 chemical shifts prevents the determination of the orientation of C-12 and C-18 relative to C-3 and thus the C-11 configuration. Based on the fact that **1**, **2a** and **2b** were all produced by the same terpene cyclase during fermentation and C-11 epimerization is unlikely, we propose that the C-11 configuration is the same in all of these molecules. This is supported by the strong similarity in ^1H and ^{13}C NMR data between **2a** and **2b** at the C-4, C-11, C-12 and C-18 positions, supporting the assertion that these two molecules have the same relative configuration at these positions. Finally, a NOESY correlation between H-7 and H_3-20 were both in axial orientations on each ring in a *syn* relative configuration. Based on this data, the relative configuration of **2b** was determined to be 1*S*, 4*R*, 7*R*, 9*S*, 11*S*.

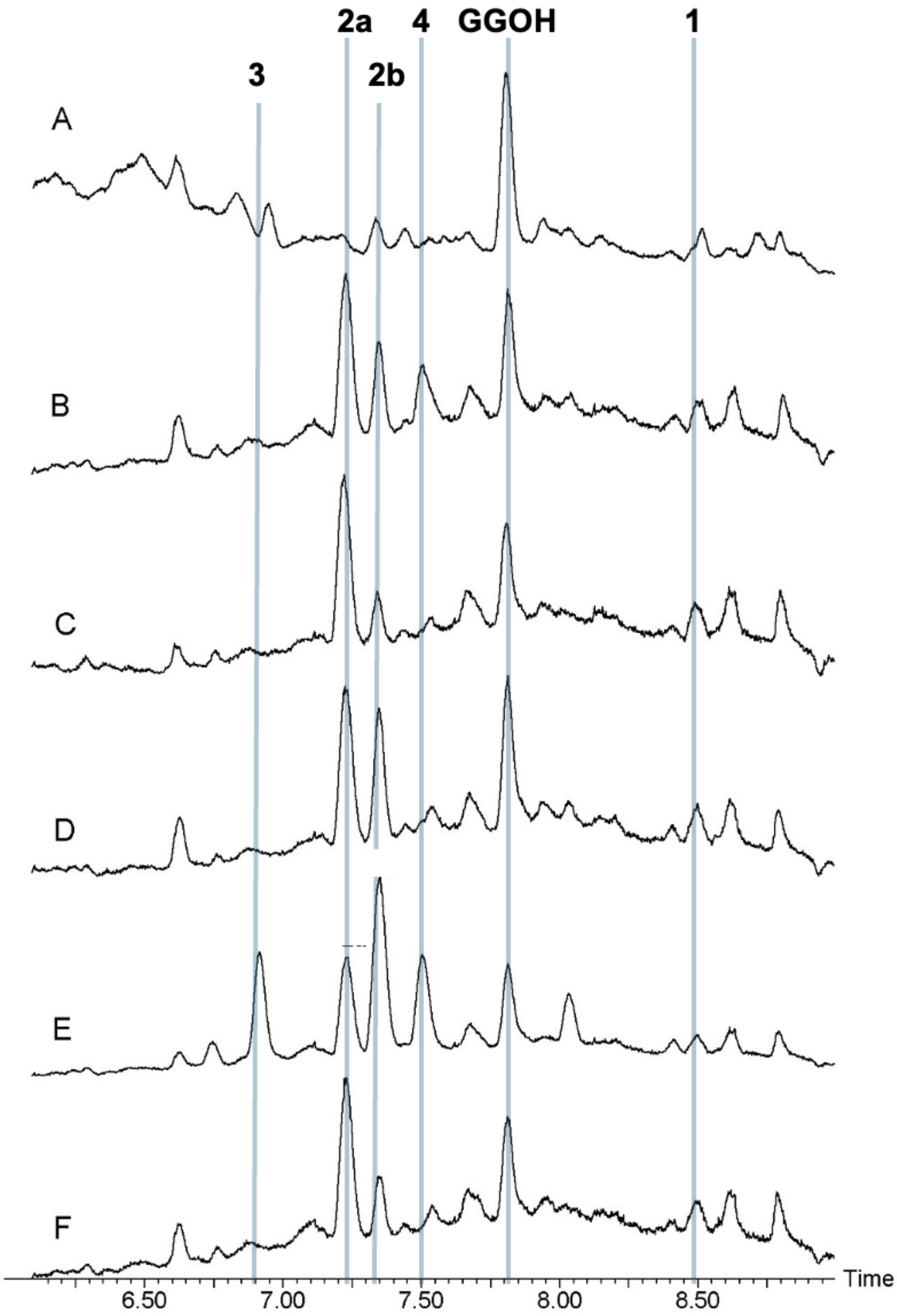

Figure S2. Positive mode LCMS TIC trace of crude extracts from *S. cerevisiae* showing the presence of compounds **2-4** when an elisabethatriene synthase is co-expressed with a P450. A) TPS + pESC-URA with only HcCPR (negative control). B) TPS + RhCYP +HcCPR C) TPS + HcCYP +HcCPR D) TPS + ChCYP +HcCPR E) TPS + AeCYP +HcCPR F) TPS + AbCYP +HcCPR.

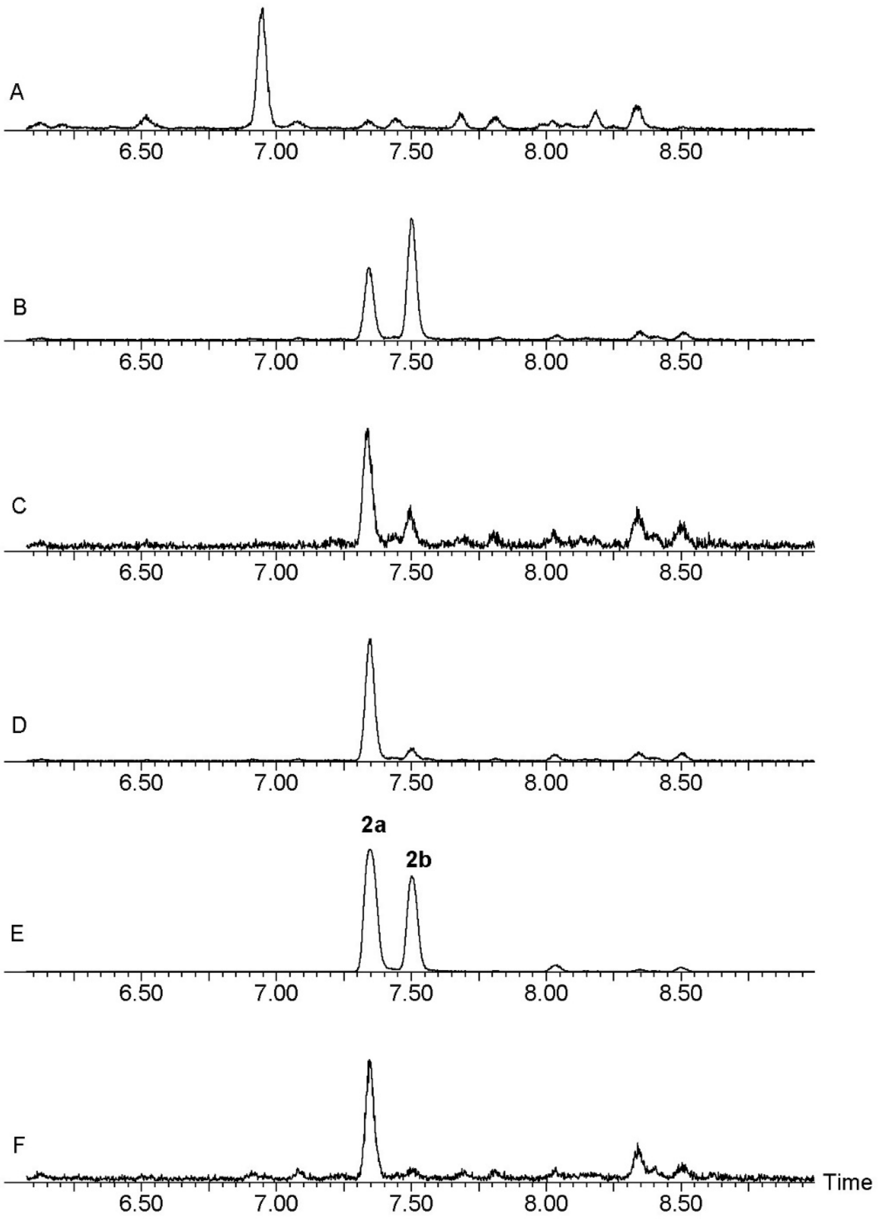

Figure S3. Positive mode LCMS EIC trace at 289.2 *m/z* trace of *S. cerevisiae* extracts showing the presence of compounds **2a** and **2b** when an elisabethatriene synthase is co-expressed with a P450. A) TPS + pESC-URA with only HcCPR (negative control). B) TPS + RhCYP +HcCPR C) TPS + HcCYP +HcCPR D) TPS + ChCYP +HcCPR E) TPS + AeCYP +HcCPR F) TPS + AbCYP +HcCPR.

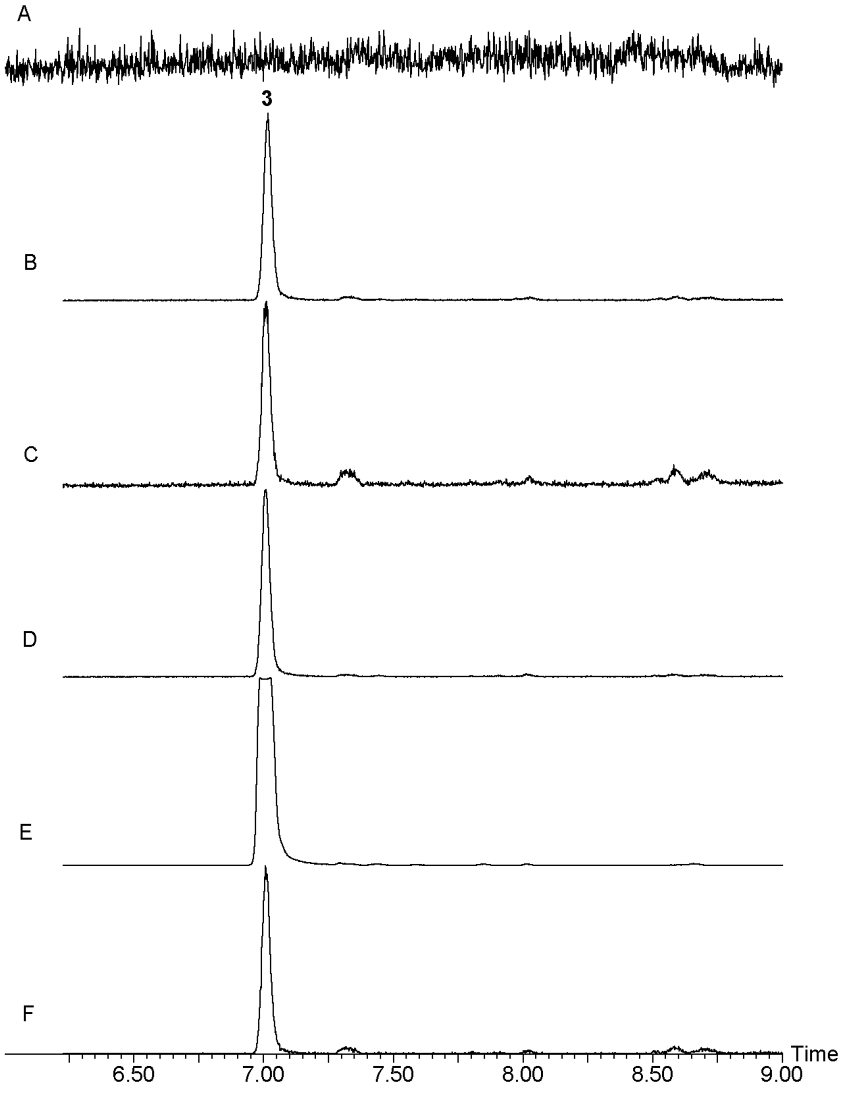

Figure S4. Negative mode LCMS EIC trace at 301.2 *m/z* trace of crude extracts from *S. cerevisiae* showing the presence of compound **3** when an elisabethatriene synthase is co-expressed with a P450. A) TPS + pESC-URA with only HcCPR (negative control). B) TPS + RhCYP +HcCPR C) TPS + HcCYP +HcCPR D) TPS + ChCYP +HcCPR E) TPS + AeCYP +HcCPR F) TPS + AbCYP +HcCPR.

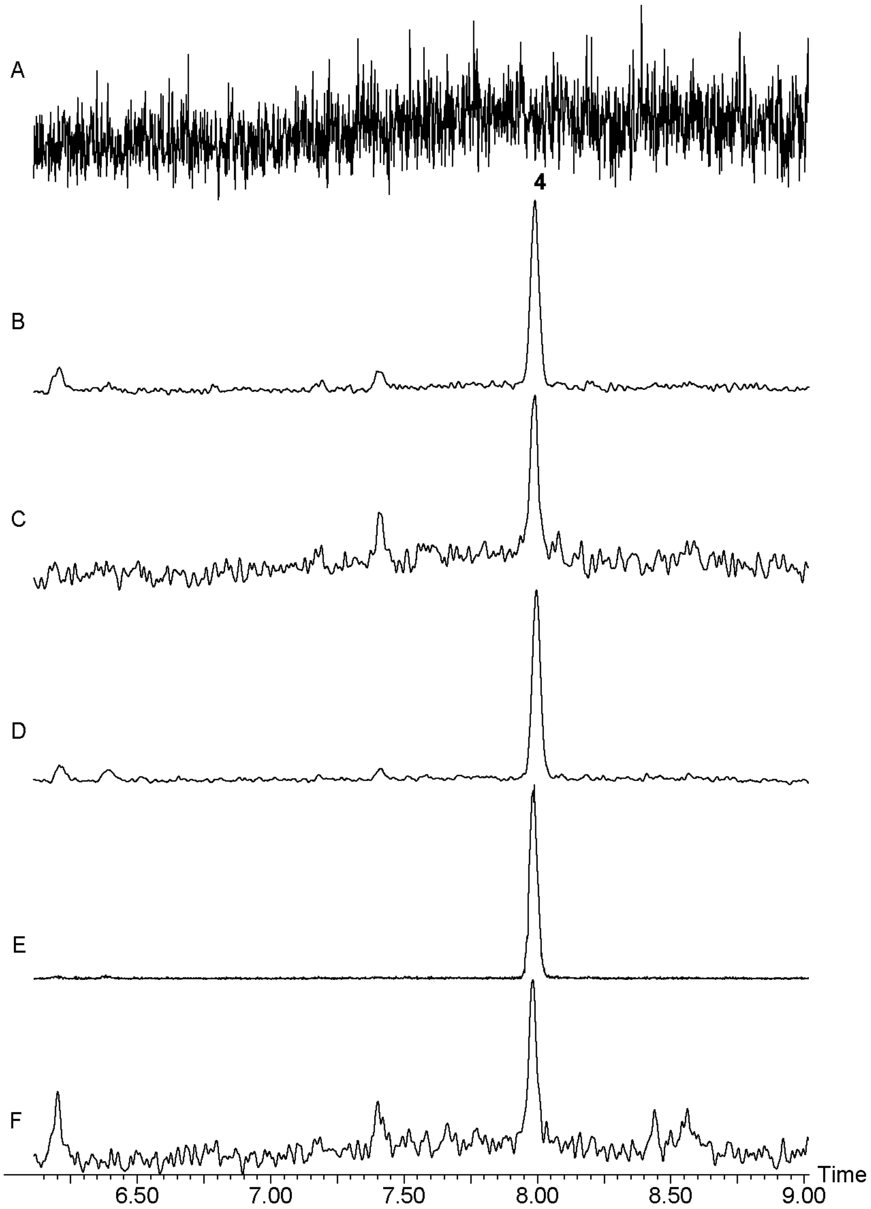

Figure S5. Positive mode LCMS EIC trace at 287.2 *m/z* trace of crude extracts from *S. cerevisiae* showing the presence of compound **4** when an elisabethatriene synthase is co-expressed with a P450. A) TPS + pESC-URA with only HcCPR (negative control). B) TPS + RhCYP +HcCPR C) TPS + HcCYP +HcCPR D) TPS + ChCYP +HcCPR E) TPS + AeCYP +HcCPR F) TPS + AbCYP +HcCPR..

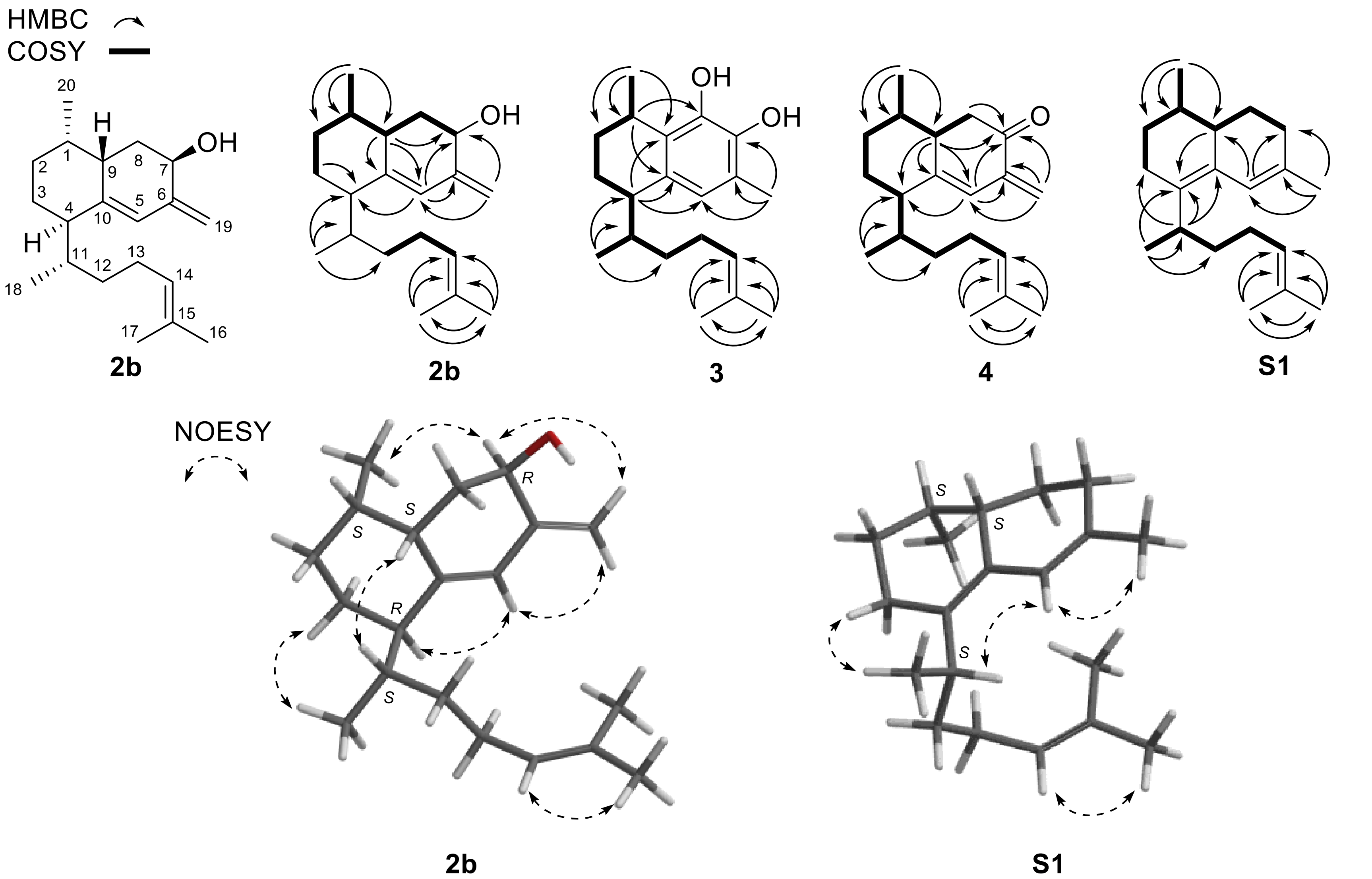

Figure S6. Selected NMR correlations for **2b**, **3**, **4** and **S1**.

Table S3. NMR data for **2b**, **3**, **4** and **S1** in $CDCl_3$. *J*-values are in Hertz.

	**2b**		**3**		**4**		**S1**	
position	δ_H[a]	δ_C[b]	δ_H[a]	δ_C[b]	δ_H[a]	δ_C[b]	δ_H[a]	δ_C[b]
1	1.99 (m) [c]	35.0 (CH)	3.08 (pd, 6.5, 2,6)	27.0 (CH)	2.06 (m) [c]	37.8 (CH)	1.21 (m) [c]	43.3 (CH)
2_a	1.83 (m) [c]	29.0 (CH_2)	1.95 (m) [c]	28.0 (CH_2)	1.86 (m) [c]	28.3 (CH_2)	1.73 (m) [c]	31.8 (CH_2)
2_b	1.32 (m) [c]	-	1.51 (m)	-	1.32 (m)	-	1.21 (m) [c]	-
3_a	1.66 (m) [c]	22.9 (CH_2)	1.82 (tdd, 13.9, 6.4, 3.8)	18.7 (CH_2)	1.78 (m)	24.5 (CH_2)	2.02 (m) [c]	24.0 (CH_2)
3_b	-	-	1.68 (m)	-	1.66 (m) [c]	-		
4	1.83 (m) [c]	49.4 (CH)	2.65(p, 3.9)	39.6 (CH)	2.00 (m)	51.0 (CH)	-	134.1 (C)
5	5.93 (s)	124.0 (CH)	-	122.0 (C)	6.29 (s)	124.3 (CH)	6.28 (s)	121.0 (CH)
6	-	146.5 (C)	-	120.8 (C)	-	139.2 (C)	-	135.1 (C)
7_a	4.39 (br t, 6,7)	68.5 (CH)	-	139.1 (C)	-	200.1 (C)	2.15 (m) [c]	31.2 (CH_2)
7_b	-	-	-	-	-	-	2.04 (m) [c]	
8_a	1.76 (m) [c]	35.6 (CH_2)	-	140.9 (C)	2.69 (dd, 16.8, 8.8)	43.3 (CH_2)	2.12 (m) [c]	28.2 (CH_2)
8_b	-	-	-	-	2.45 (d, 16.8)	-	1.15 (qd, 12.2, 5.2)	
9	2.55 (br q, 5.9)	35.8 (CH)	-	127.5 (C)	2.92 (dd, 8.8, 4.3)	39.3 (CH)	1.64 (br q, 5.9)	43.3 (CH)
10	-	144.2 (C)	-	132.2 (C)	-	145.3 (C)	-	129.2 (C)
11	1.71 (m) [c]	31.6 (CH)	1.93 (m) [c]	38.7 (CH)	1.84 (m)	30.0 (CH)	2.92 (sxt, 7.0) [c]	32.9 (CH)
12_a	1.34 (m) [c]	34.7 (CH_2)	1.46 (m)	35.8 (CH_2)	1.38 (tdd, 13.4, 10.0, 3.0)	35.0 (CH_2)	1.38 (spt, 7.4) [c]	35.6 (CH_2)
12_b	0.91 (m) [c]	-	1.35 (m)	-	1.00 (tdd, 13.4, 10.0, 5.0)	-	-	
13_a	2.03 (m) [c]	25.4 (CH_2)	2.09 (m)	26.3 (CH_2)	2.07 (m) [c]	25.3 (CH_2)	1.92 (br q, 7.1)	26.2 (CH_2)
13_b	1.83 (m) [c]	-	2.00 (m)	-	1.90(m) [c]	-	-	-
14	5.02 (t, 7.0)	125.0 (CH)	5.14 (t, 7.)	124.8 (CH)	5.07 (t, 7.0)	124.8 (CH)	5.14 (t, 7.0)	125.2 (CH)
15	-	131.1 (C)	-	131.2 (C)	-	131.5 (C)	-	131.3 (C)
16	1.65 (s)	25.7 (CH_3)	1.73 (s)	25.8 (CH_3)	1.68 (s)	25.9 (CH_3)	1.71 (s)	25.9 (CH_3)
17	1.57 (s)	17.7 (CH_3)	1.64 (s)	17.6 (CH_3)	1.60 (s)	17.2 (CH_3)	1.60 (s)	17.9 (CH_3)
18	0.90 (d, 7.0 Hz)	17.4 (CH_3)	0.73 (d, 6.8)	18.8 (CH_3)	0.96 (d, 6.6)	17.3 (CH_3)	0.98 (d, 6.7)	18.8 (CH_3)
19_a	5.01 (s)	108.4 (CH_2)	2.24 (s)	15.7 (CH_2)	5.81 (s)	116.3 (CH_2)	1.79 (s)	24.1 (CH_3)
19_b	4.83 (s)	-	-	-	5.05 (s)	-	-	-
20	0.87 (d, 7.0 Hz)	15.1 (CH_3)	1.22 (d, 7.1)	21.0 (CH_3)	0.75 (d, 7.0)	12.7 (CH_3)	1.03 (d, 5.4)	20.6 (CH_3)
-OH	1.36 (m) [c]	-	5.10 (br s) [d]	-		-	-	-
-OH			4.77 (br s) [d]					

[a] Measured at 600 MHz. [b] Measured at 125 MHz. [c] Overlapping resonances. [d] Very broad resonance.

III. Semisynthesis of proposed biosynthetic intermediates and feeding studies in yeast

A. Semisynthesis of proposed biosynthetic intermediates

Synthesis of ketone 4 by Swern oxidation. Oxalyl chloride (18 µl, 0.20 mmol, 2 eq.) was added to a solution of dimethyl sulfoxide (15 µl, 0.20 mmol, 2 eq.) in dichloromethane (2 ml) with stirring at -80 °C. After 20 min, a mixture of elisabethatrienol (**2a**) and *epi*-elisabethatrienol (**2b**) (30 mg, 0.10 mmol, 1 eq.) was added as a solution in dichloromethane (1 ml). After another 20 min, triethylamine (70 µl, 0.50 mmol, 5 eq.) was added then the reaction mixture removed from the bath and allowed to warm to room temperature for 30 min. The reaction was quenched by addition of aqueous sodium bicarbonate then the dichloromethane layer collected and dried over anhydrous sodium sulfate and evaporated. The residue was partitioned between hexane and water to remove polar byproducts, and the hexane layer was evaporated. The hexane soluble residue was purified by preparative HPLC using a phenylhexyl column and a gradient of 75% acetonitrile in water increasing to 100 % acetonitrile over 25 min at 8 ml/min flow. A peak at Rt 21.7 min was collected and evaporated to yield ketone **4** (10.5 mg, 0.037 mmol, 37% yield).

Ketone **4**: $[\alpha]_D^{20} + 6.5$ (*c* 0.263, $CHCl_3$); IR (polyethylene film) ν_{max} 2975, 2921, 2848, 1729, 1684, 1494, 1462, 1377, 1330, 1268, 1081,1045 cm^{-1}; NMR see Table S3; HRESIMS *m/z* 287.2409 (calcd for $C_{20}H_{31}O^+$, 287.2370).

Synthesis of erogorgiaene (6) by acid-catalyzed dehydration. A mixture of elisabethatrienol (**2a**) and *epi*-elisabethatrienol (**2b**) (7 mg, 0.0242 mmol) was dissolved in dichloromethane (1 ml), treated with *para*-toluene sulfonic acid (1 mg), and stirred for 3 h. The reaction was quenched with aqueous sodium bicarbonate then the dichloromethane layer evaporated. The residue was dissolved in hexane, loaded onto a column of silica gel then eluted with hexane followed by1:1 hexane/diethyl ether to afford erogorgiaene (**6**) (5.8 mg, 0.0214 mmol, 88% yield).

Erogorgiaene (**6**): $[\alpha]_D^{20} + 21.0$ (*c* 0.105, $CHCl_3$); ^{1}H NMR (500 MHz, $CDCl_3$) δ 7.15 (d, *J* = 7.8 Hz, 1H), 7.04 (s, 1H), 6.96 (d, *J* = 7.8 Hz, 1H), 5.19 (t, *J* = 7.3 Hz, 1H), 2.88 (d, *J* = 10.2 Hz, 1H), 2.74 (s, 1H), 2.32 (s, 3H), 2.15 (dq, *J* = 13.9, 7.1 Hz, 1H), 2.12 – 1.98 (m, 2H), 1.94 (dd, *J* = 12.0, 5.2 Hz, 1H), 1.83 (ddd, *J* = 13.4, 6.6, 3.4 Hz, 1H), 1.74 (s, 3H), 1.66 (s, 3H), 1.62 – 1.49 (m, 2H), 1.47 (m, 1H), 1.36 (m, 1H), 1.30 (d, J = 6.8 Hz, 3H), 0.66 (d, *J* = 6.8 Hz, 3H); ^{13}C NMR (125 MHz, $CDCl_3$) δ 140.6, 140.1, 134.8, 131.4, 128.3, 126.6, 126.1, 125.0, 41.6, 37.1, 35.4, 33.0, 31.9, 26.5, 25.9, 22.1, 21.7, 21.3, 17.7, 14.7.; HRESIMS *m/z* 271.2445 (calcd for $C_{20}H_{31}^+$, 271.2421). For previous data, see reference SI-5.

Synthesis of 7-hydroxyerogorgiaene (7) by oxidation with PCC. A mixture of elisabethatrienol (**2a**) and *epi*-elisabethatrienol (**2b**) (15 mg, 0.0520 mmol, 1 eq.) was dissolved in 1:1 acetone/dichloromethane (3 ml) in a vial with stirring. Pyridinium chlorochromate (30 mg, 1.5 eq.) was added and the reaction monitored by TLC. After 3 h, the reaction was quenched by addition of isopropanol, then the solvent evaporated under vacuum. The residue was partitioned between dichloromethane and aqueous ethylenediaminetetraacetic acid. The dichloromethane layer was filter through silica gel in a pipette, then

[SI-5] Rodríguez, A. D.; Ramírez, C. Serrulatane Diterpenes with Antimycobacterial Activity Isolated from the West Indian Sea Whip *Pseudopterogorgia elisabethae*. *J. Nat. Prod.* **2001**, *64* (1), 100–102. https://doi.org/10.1021/np000196g.

treated with *para*-toluene sulfonic acid (1 mg) and stirred overnight. The reaction was checked by TLC then loaded directly onto a column of silica gel and eluted with 1:1 hexane/diethyl ether to afford 7-hydroxyerogorgiaene (**7**) (8.6 mg, 0.0300 mmol, 58% yield).

7-hydroxyerogorgiaene (**7**): $[\alpha]_D^{20} + 26.2$ (*c* 0.017, $CHCl_3$); ^1H NMR (500 MHz, $CDCl_3$) δ 6.96 (s, 1H), 6.68 (s, 1H), 5.18 (t, *J* = 7.0 Hz, 1H), 4.54 (s, 1H), 2.83 (m, 1H), 2.70 (m 1H), 2.23 (s, 3H), 2.11 (m, 1H), 2.04 (m, 1H), 1.92 (m, 1H), 1.80 (m, 1H), 1.73 (s, 3H), 1.65 (s, 3H), 1.60 (m, 1H), 1.44 (m, 1H), 1.30 (m, 2H), 1.26 (d, *J* = 7.0 Hz, 3H), 0.66 (d, *J* = 7.0 Hz, 3H); ^{13}C NMR (125 MHz, $CDCl_3$) δ 151.3, 142.4, 132.2, 131.2, 129.9, 124.9, 120.8, 112.7, 40.9, 36.9, 35.2, 31.9, 31.8, 26.3, 25.8, 21.8, 21.7, 17.7, 15.5, 14.4; HRESIMS *m/z* 287.2448 (calcd for $C_{20}H_{31}O^+$, 287.2370). For previous data, see reference SI-4.

B. Feeding studies in yeast

Yeast strains containing either AeCYP and HcCPR on pESC-URA or pESC-URA with only HcCPR were seeded into SC -ura (5 ml) with 2% dextrose and grown overnight at 30 °C with shaking at 180 rpm. This was used to inoculate SC -ura (25 ml) containing 4% galactose and the cultures were grown overnight at 20 °C. To the induced culture was added filter sterilized bovine serum albumin in media (0.5 mg/ml). Aliquots (2 ml) of the cultures were distributed into sterile test tubes. Approximately 100 µg of each hypothetical intermediate were added as solution in ethanol (50 µl). The cultures were shaken at 20 °C and 220 rpm for 3 days, then extracted by vortexing with diethyl ether (2 ml). The suspension was centrifuged and the ether layer pipetted off and dried briefly under a stream of air. The residue was dissolved in methanol and analyzed by LCMS using the method described above.

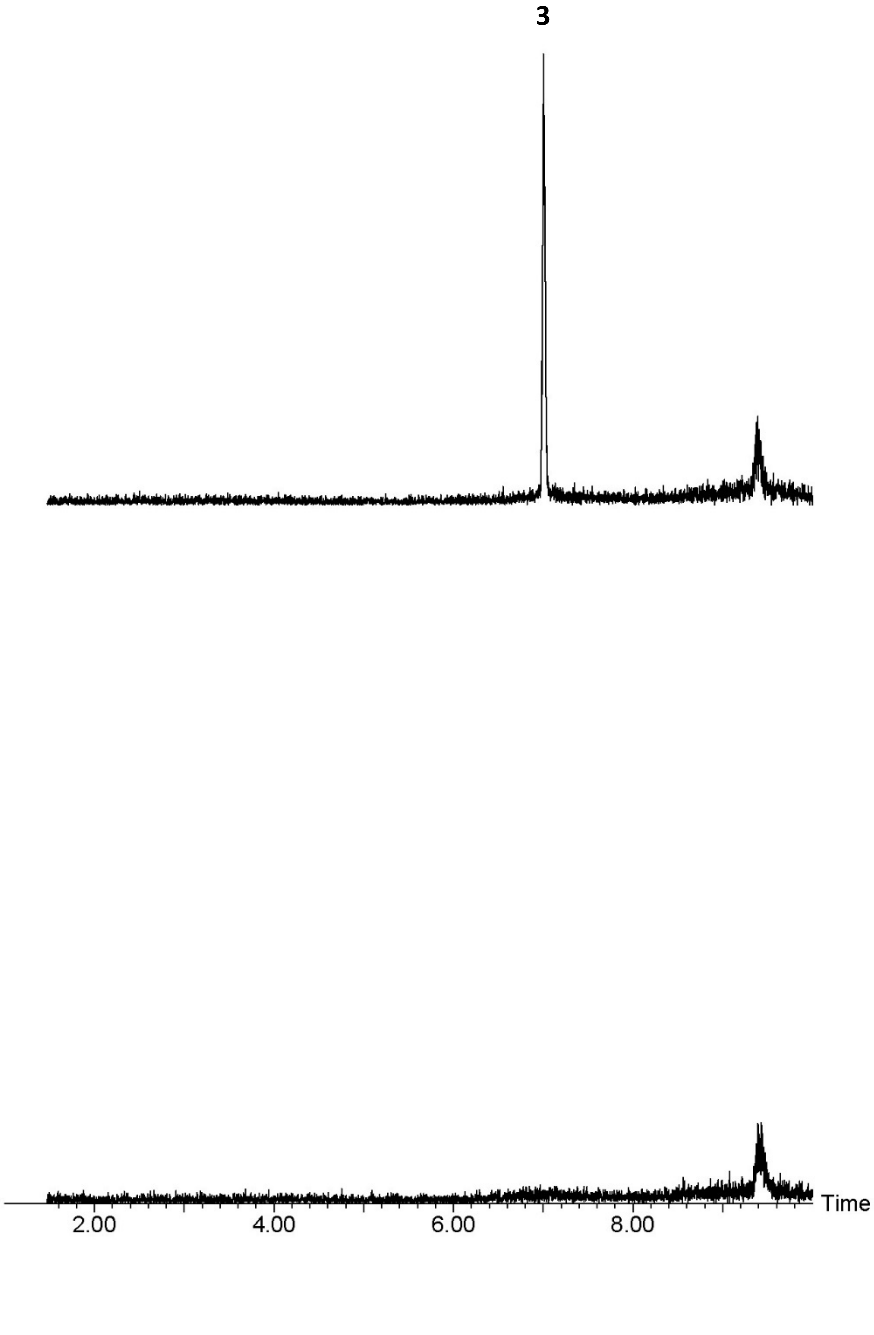

Figure S7. Compound **3** is produced from **1**. Negative mode LCMS EIC trace at 301.2 *m/z* of product of **1** incubated with *S. cerevisiae* harboring AeCYP or plasmid without AeCYP. Top) Strain harboring AeCYP. Bottom) Strain without AeCYP. Both chromatograms are represented in the same scale.

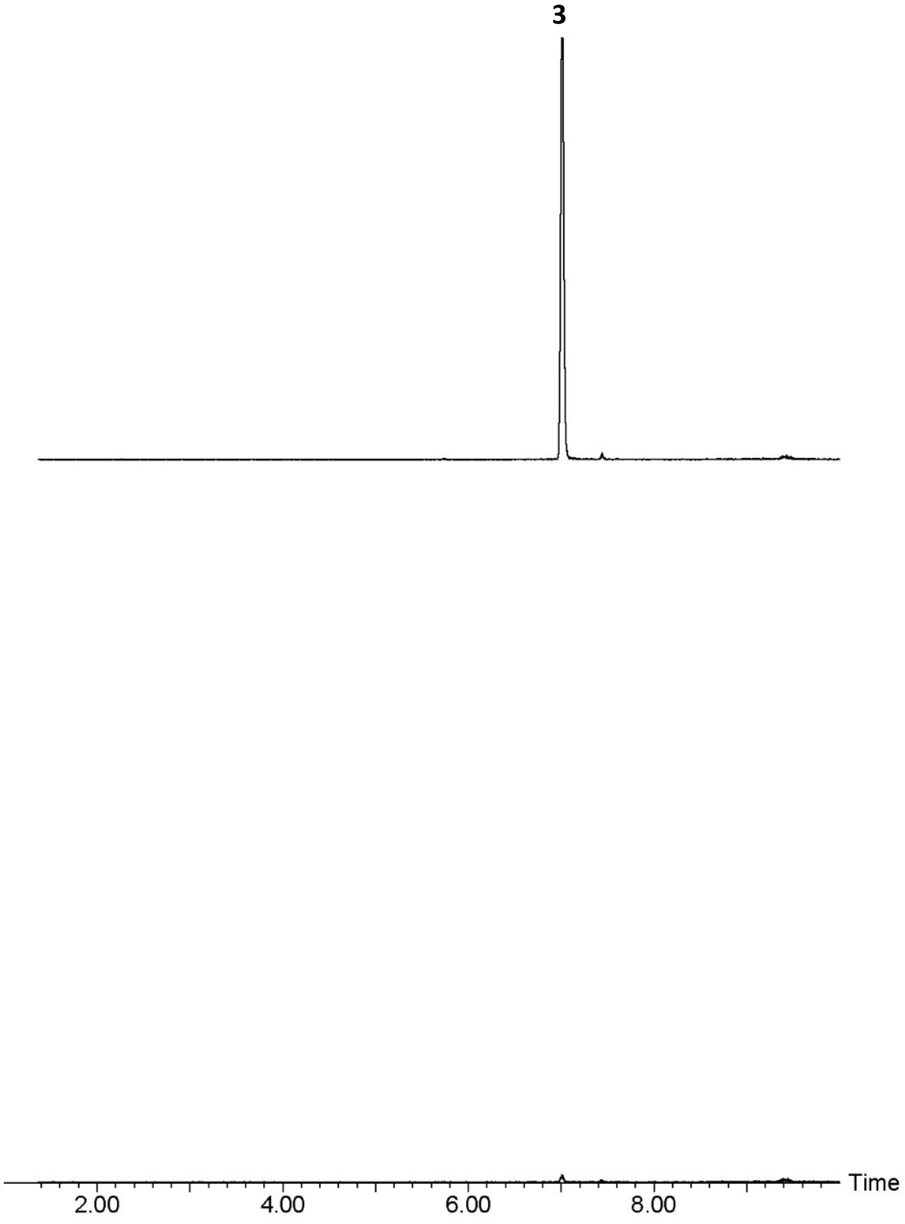

Figure S8. Compound **3** is produced from **2a**. Negative mode LCMS EIC trace at 301.2 *m/z* of product of **2a** incubated with *S. cerevisiae* harboring AeCYP or plasmid without AeCYP. Top) Strain harboring AeCYP. Bottom) Strain without AeCYP. Both chromatograms are represented in the same scale.

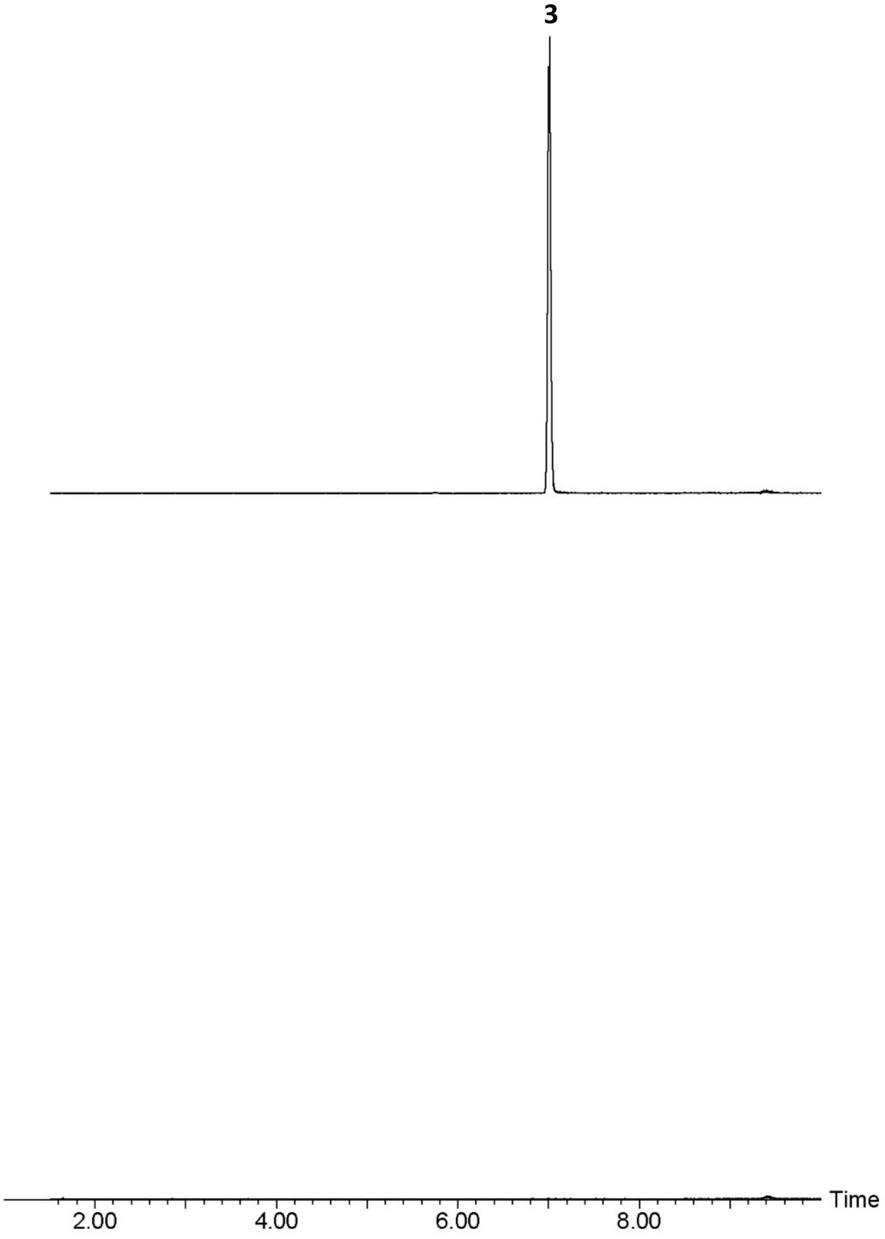

Figure S9. Compound **3** is produced from **2b**. Negative mode LCMS EIC trace at 301.2 *m/z* of product of **2b** incubated with *S. cerevisiae* harboring AeCYP or plasmid without AeCYP. Top) Strain harboring AeCYP. Bottom) Strain without AeCYP. Both chromatograms are represented in the same scale.

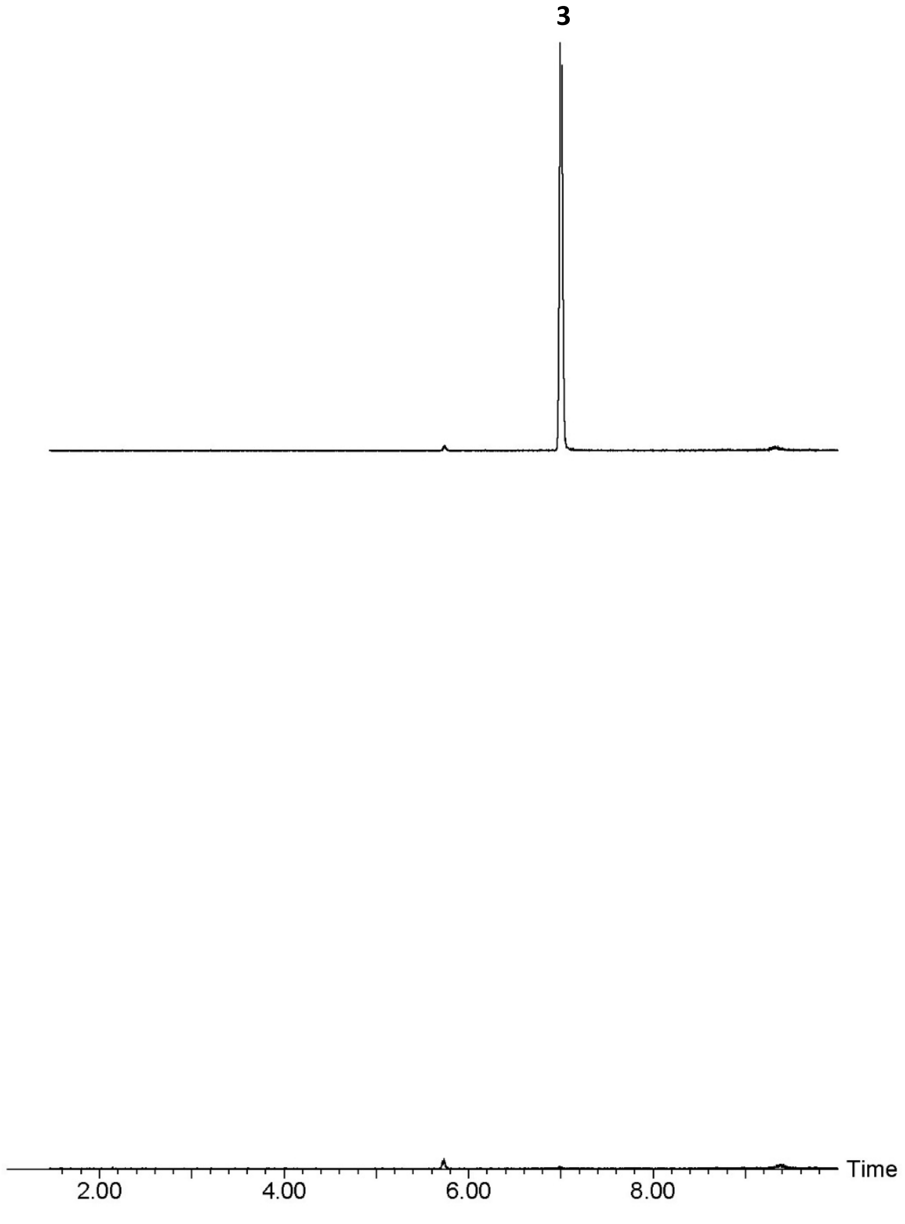

Figure S10. Compound **3** is produced from **4**. Negative mode LCMS EIC trace at 301.2 *m/z* of product of **4** incubated with *S. cerevisiae* harboring AeCYP or plasmid without AeCYP. Top) Strain harboring AeCYP. Bottom) Strain without AeCYP.

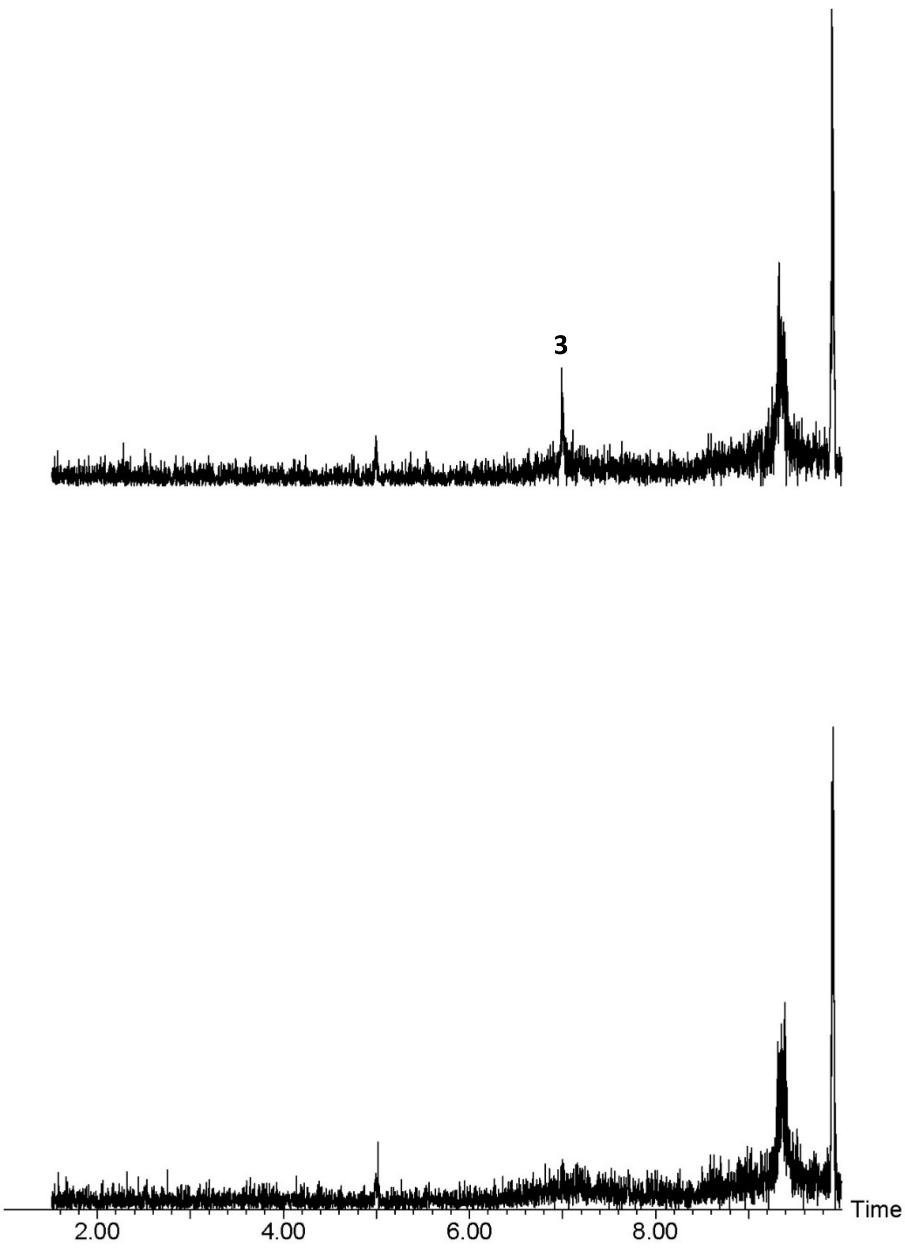

Figure S11. Only trace **3** is produced from **6**. Negative mode LCMS EIC trace at 301.2 *m/z* of product of **6** incubated with *S. cerevisiae* harboring AeCYP or plasmid without AeCYP. Top) Strain harboring AeCYP. Bottom) Strain without AeCYP.

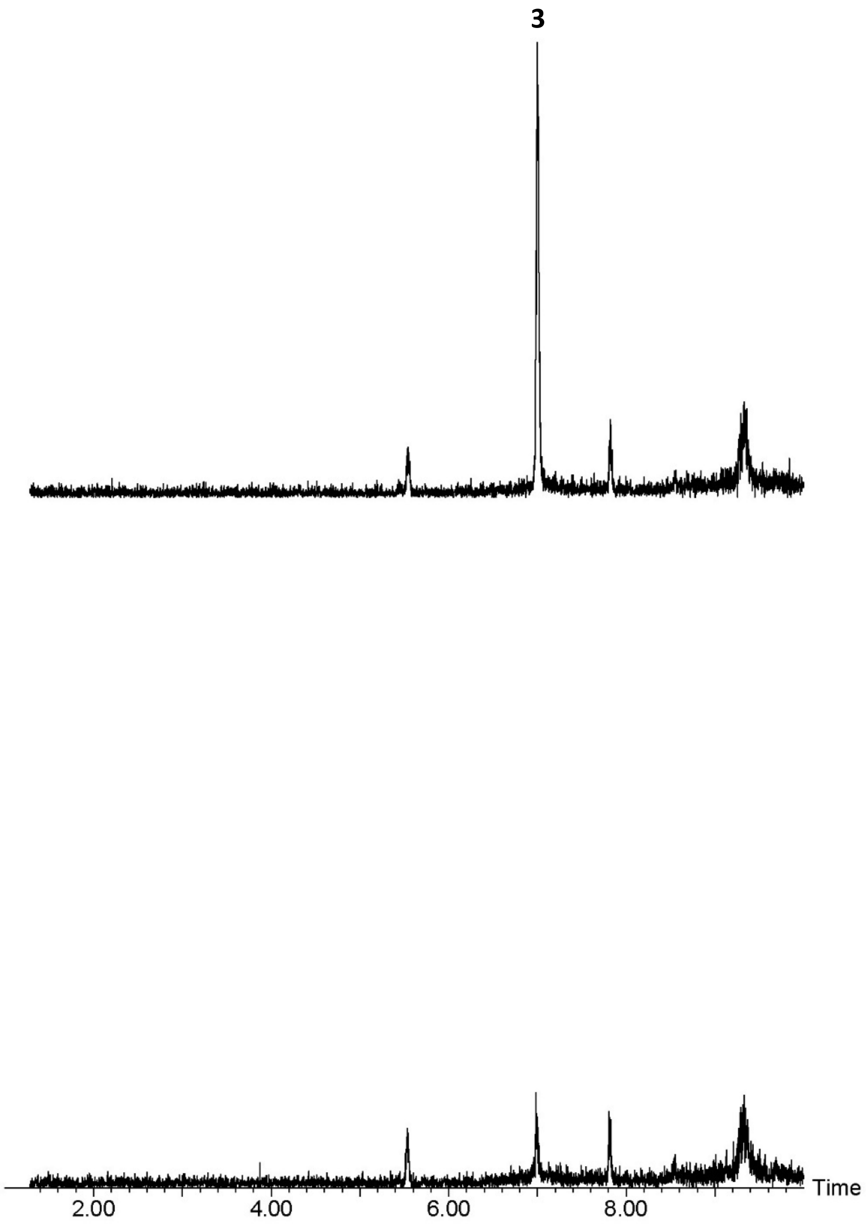

Figure S12. Trace **3** is produced from **7**. Negative mode LCMS EIC trace at 301.2 *m/z* of product of **7** incubated with *S. cerevisiae* harboring AeCYP or plasmid without AeCYP. Top) Strain harboring AeCYP. Bottom) Strain without AeCYP.

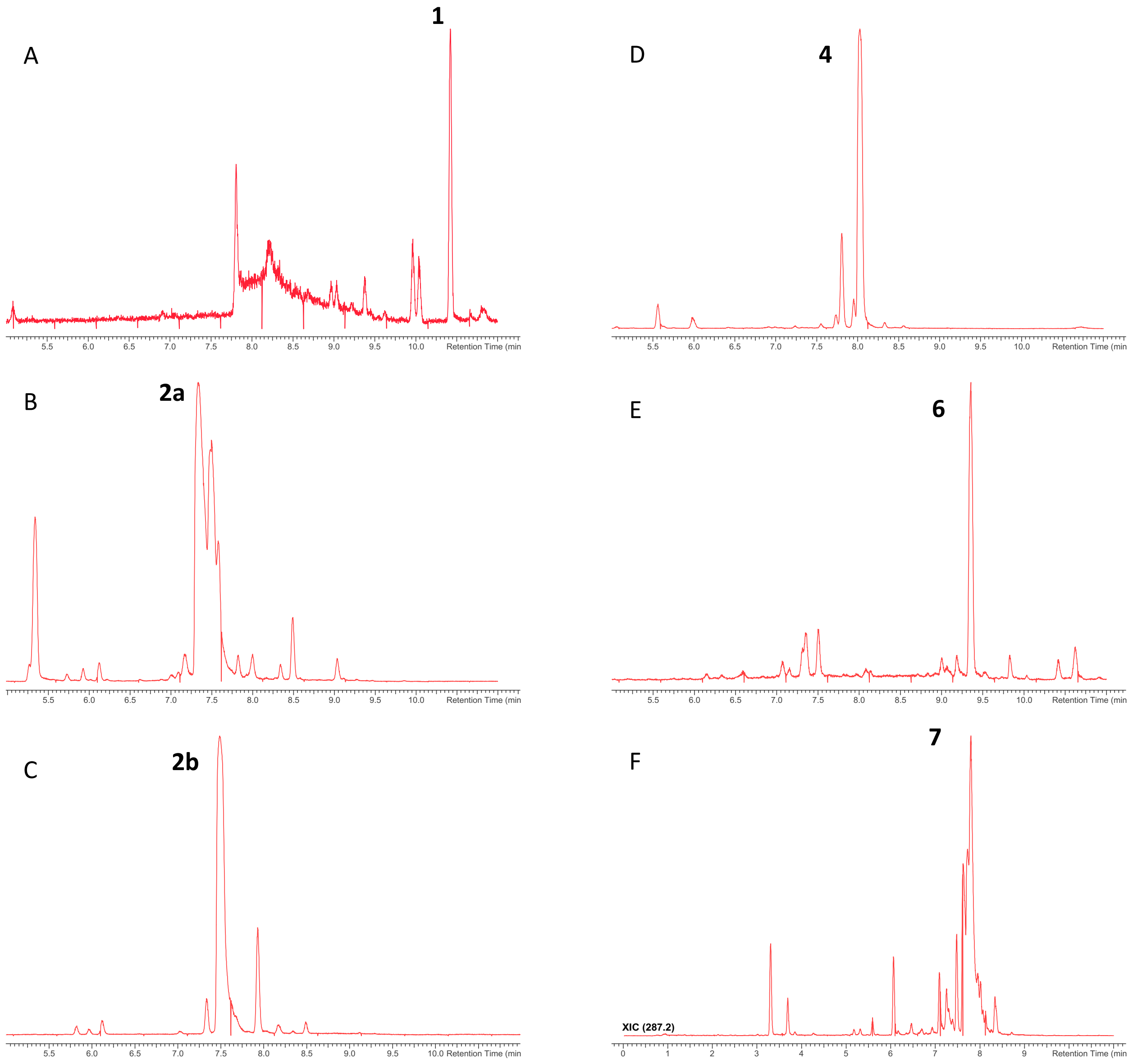

Figure S13. LCMS traces for yeast extracts after AeCYP biotransformation assays showing EIC for the *m/z* of starting substrate. A) Trace for elisabethatriene (**1**) addition at *m/z* 273.24. B) Trace for elisabethatrienol (**2a**) addition at *m/z* 271.24. C) Trace for *epi*-elisabethatrienol (**2b**) addition at *m/z* 271.24. D) Trace for ketone **4** addition at *m/z* 287.24. E) Trace for erogorgiaene (**6**) addition at *m/z* 271.24. F) Trace for 7-hydroxyerogorgiaene (**7**) addition at *m/z* 287.24.

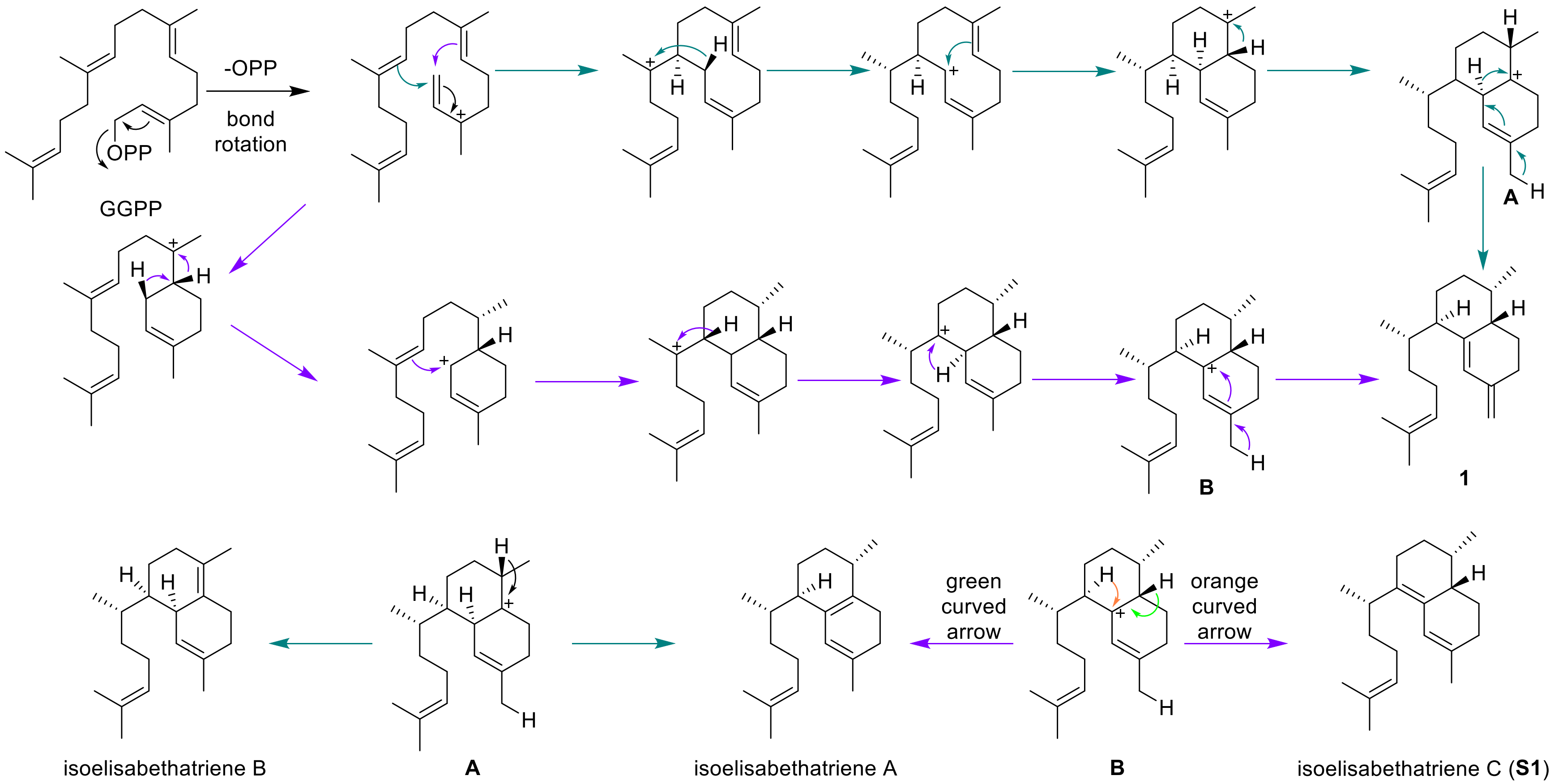

Scheme S1. Proposed cyclization mechanisms for elisabethatriene. The pathway proposed for bacterial diterpene cyclases (green) involves formation of a 10-membered ring intermediate and leads to formation of isoelisabethatriene A and isoelisabethatriene B via carbocation intermediate **A**. The pathway proposed for corals (purple) involves formation of a 6-membered ring intermediate and leads to the formation of elisabethatriene, isoelisabethatriene A and isoelisabethatriene C via carbocation intermediate **B**. This may indicate distinctive cyclization mechanisms which evolved between separate kingdoms of life. In the presence of acidic $CDCl_3$, the conversion of **S1** to **1** was observed by NMR, indicating **1** is a non-Zaitsev thermodynamically favored product. Elisabethatriene synthase might form **S1** through kinetically controlled deprotonation of **B**, which acts as an essential neutral intermediate in the cascade toward **1**.

IV. Selected NMR spectra

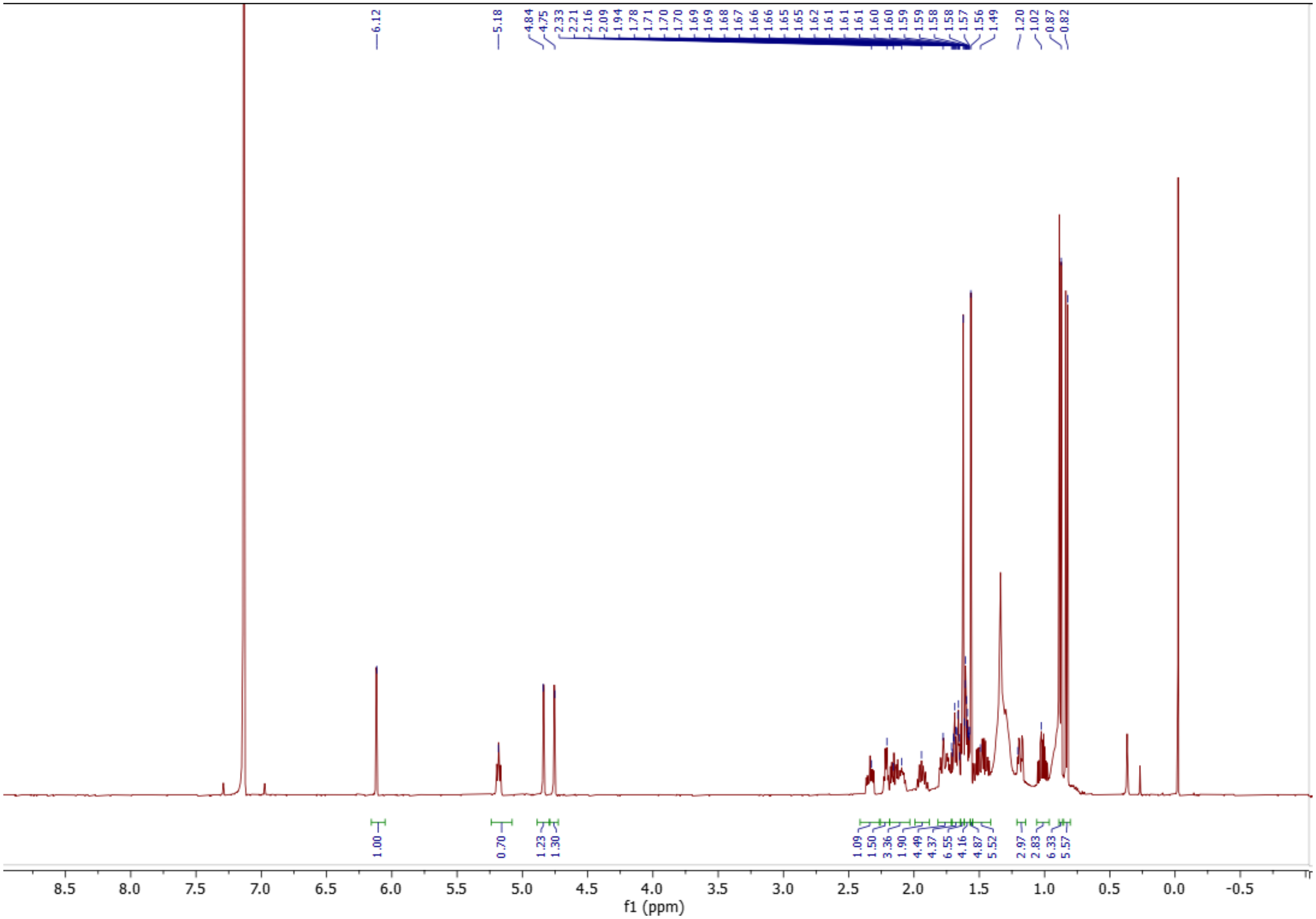

^{1}H-NMR spectrum of elisabethatriene (**1**) measured at 500 MHz in C_6D_6.

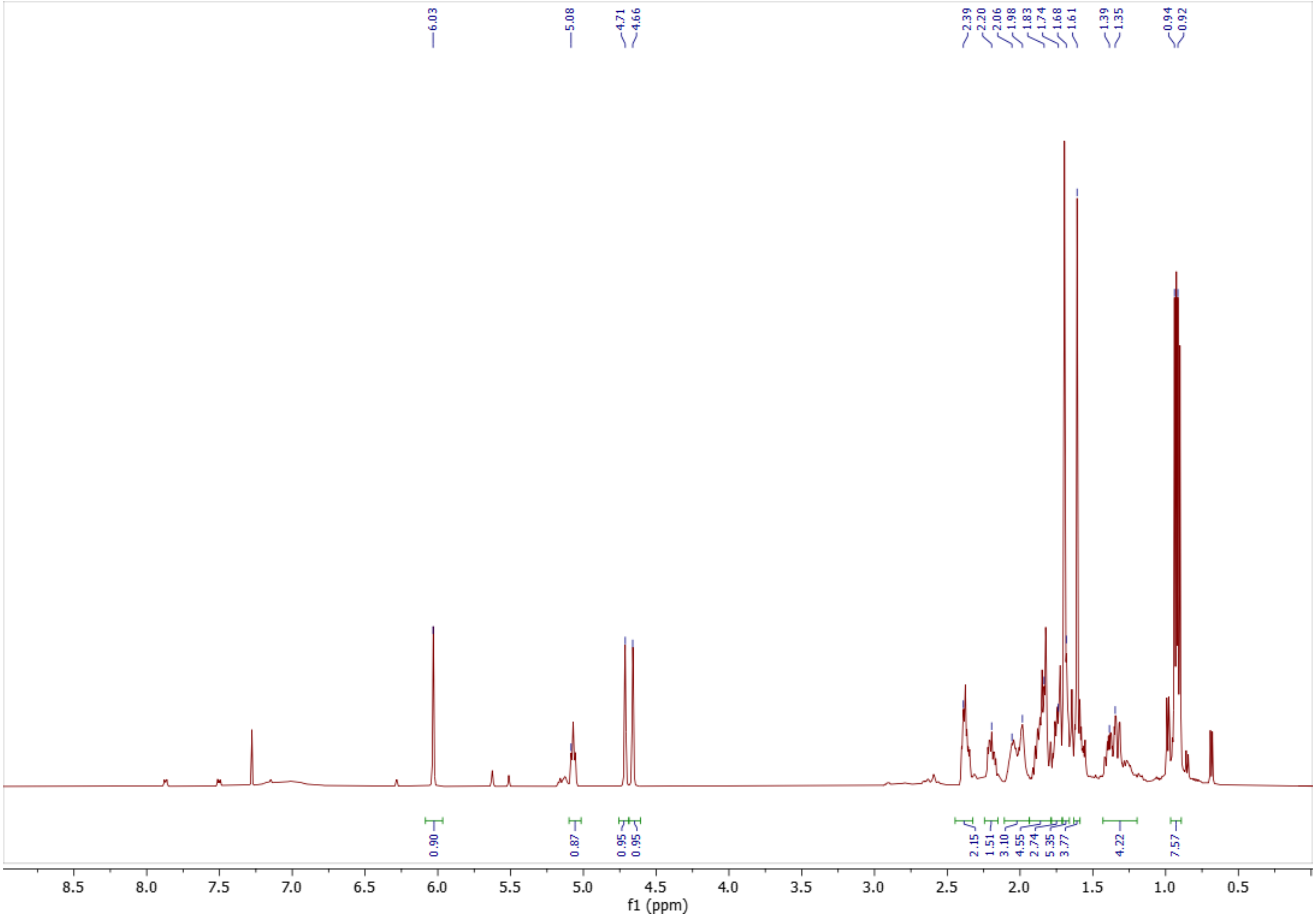

^{1}H-NMR spectrum of elisabethatriene (**1**) measured at 500 MHz in $CDCl_3$.

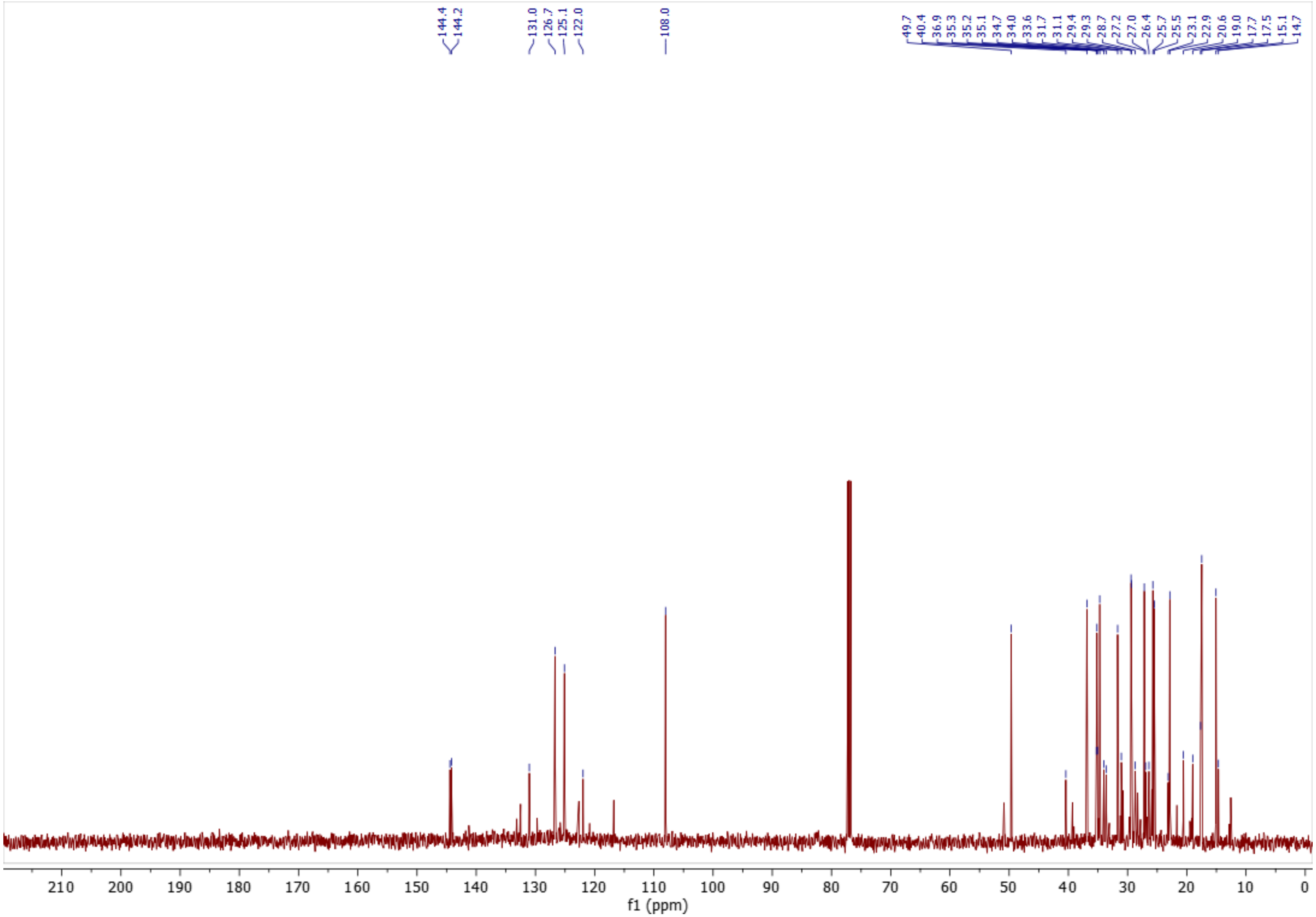

^{13}C-NMR spectrum of elisabethatriene (**1**) measured at 125 MHz in $CDCl_3$.

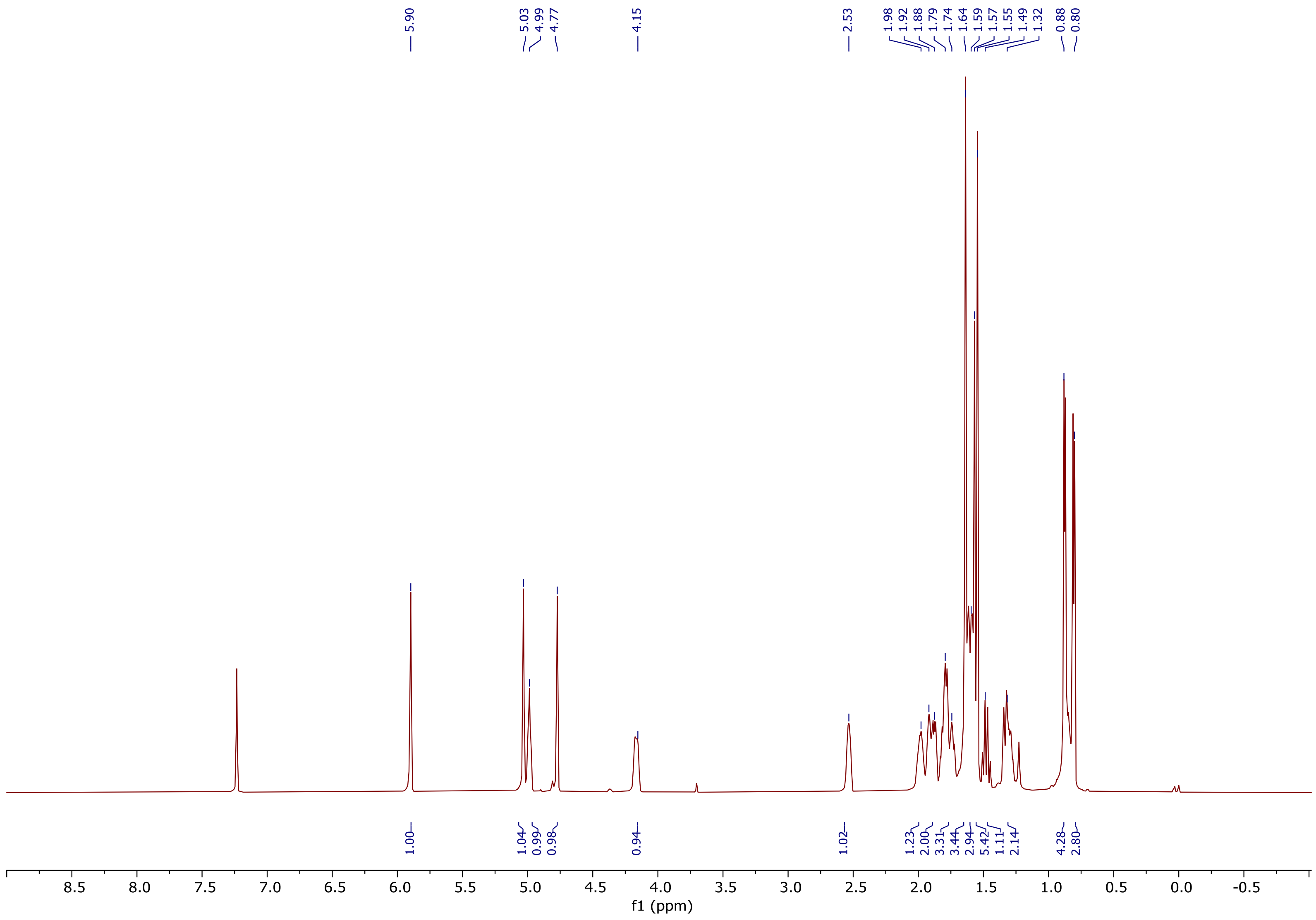

^{1}H-NMR spectrum of elisabethatrienol (**2a**) measured at 500 MHz in $CDCl_3$.

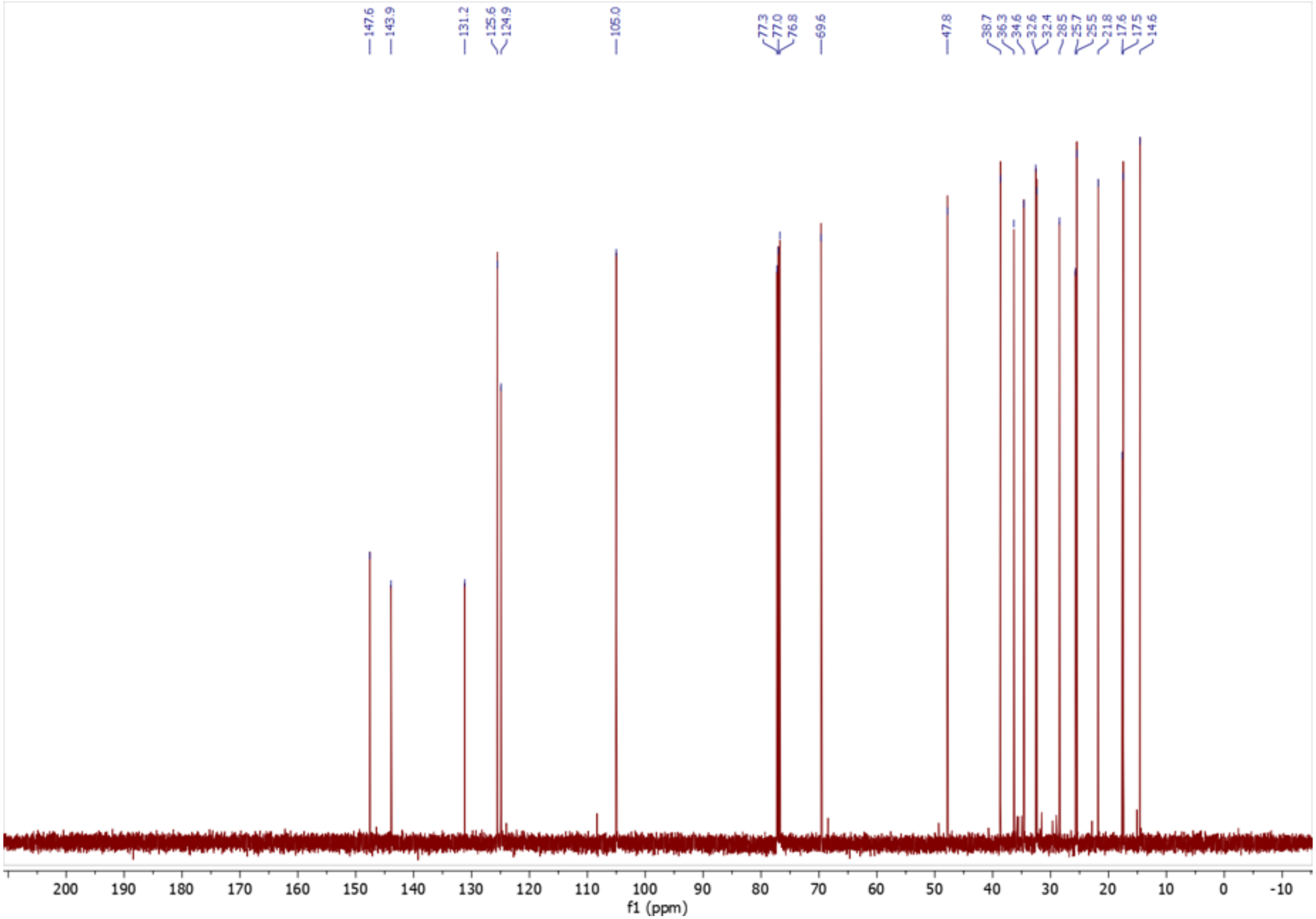

^{13}C-NMR spectrum of elisabethatrienol (**2a**) measured at 125 MHz in $CDCl_3$.

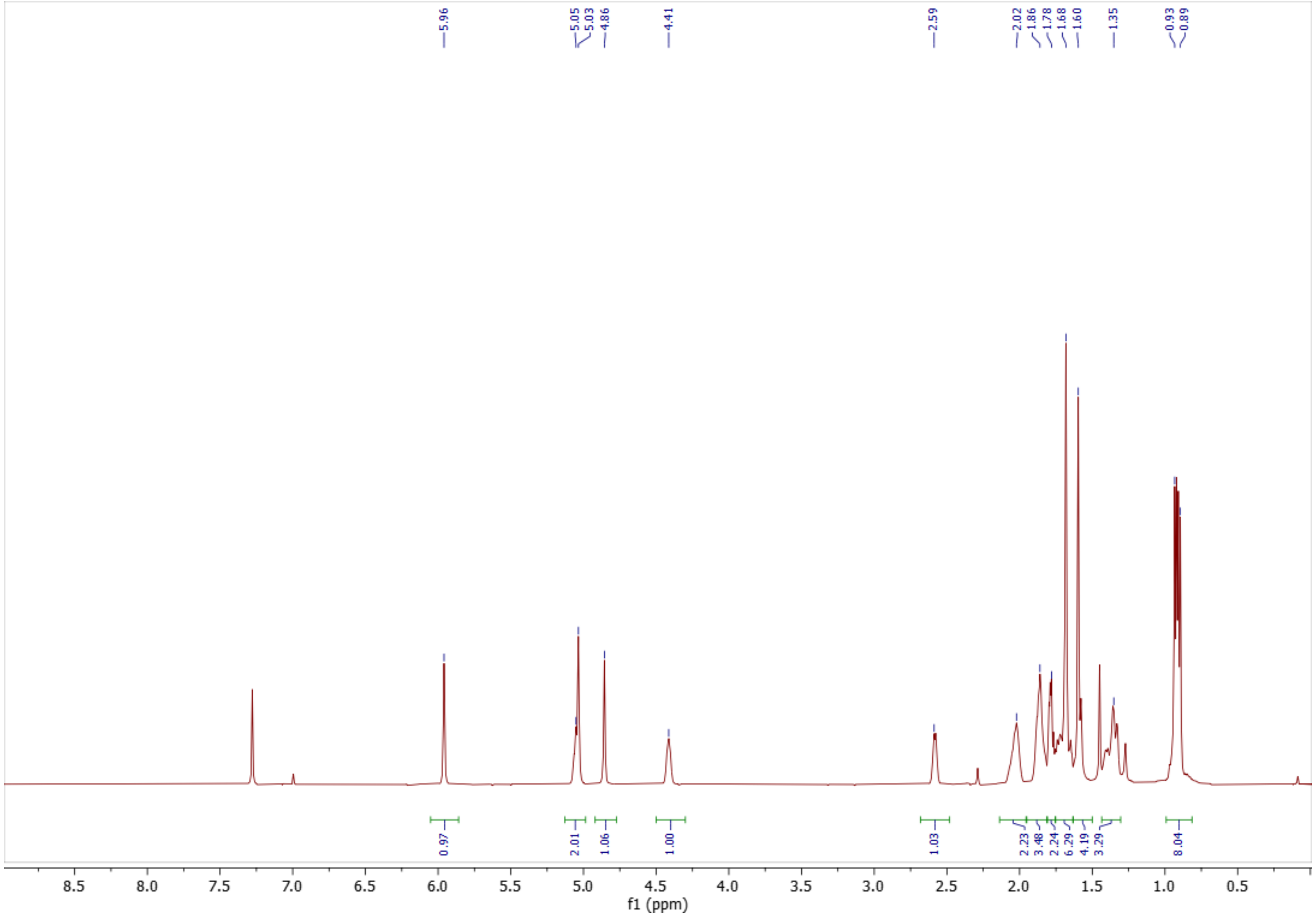

^{1}H-NMR spectrum of *epi*-elisabethatrienol (**2b**) measured at 500 MHz in $CDCl_3$.

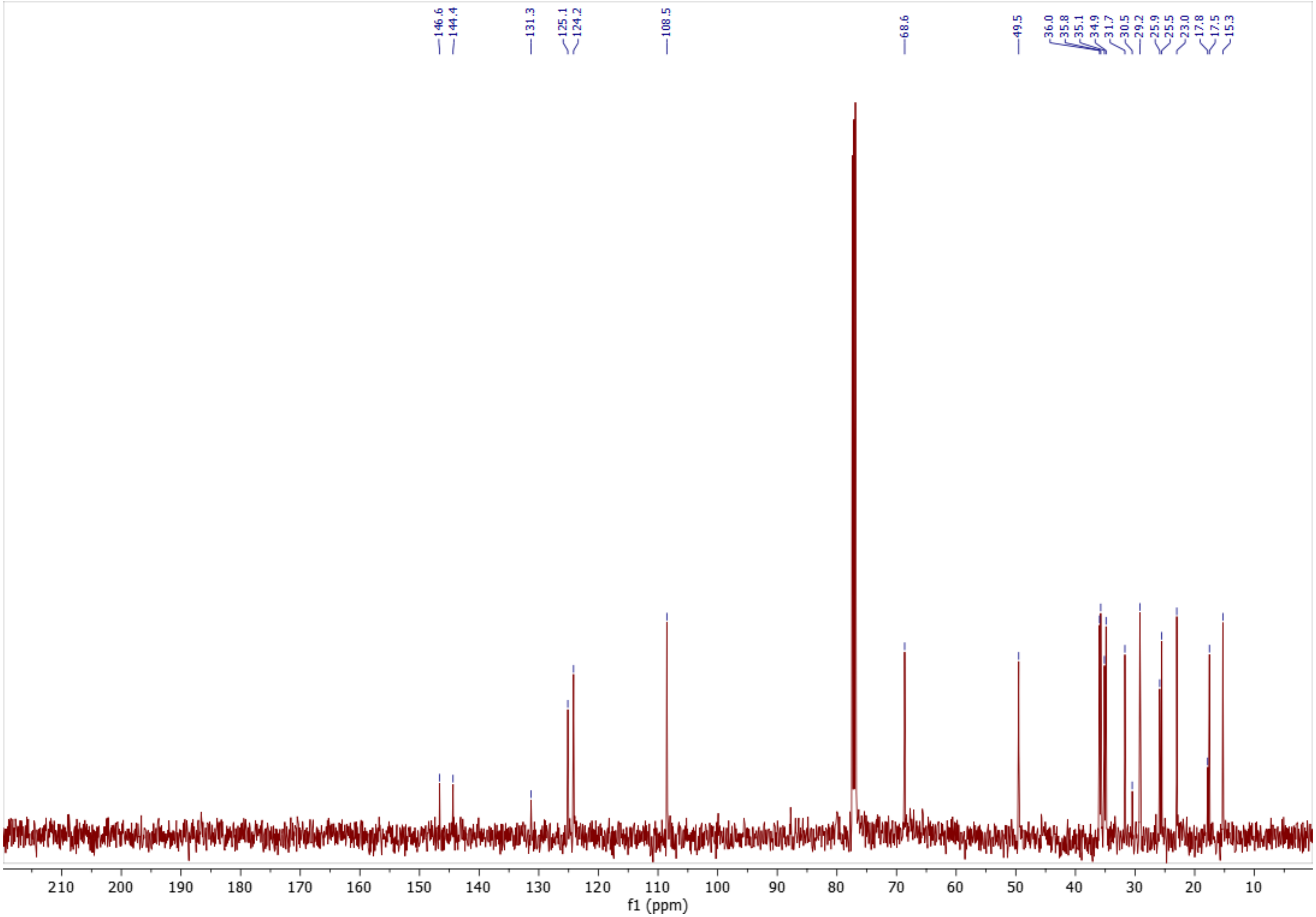

^{13}C-NMR spectrum of *epi*-elisabethatrienol (**2b**) measured at 125 MHz in $CDCl_3$.

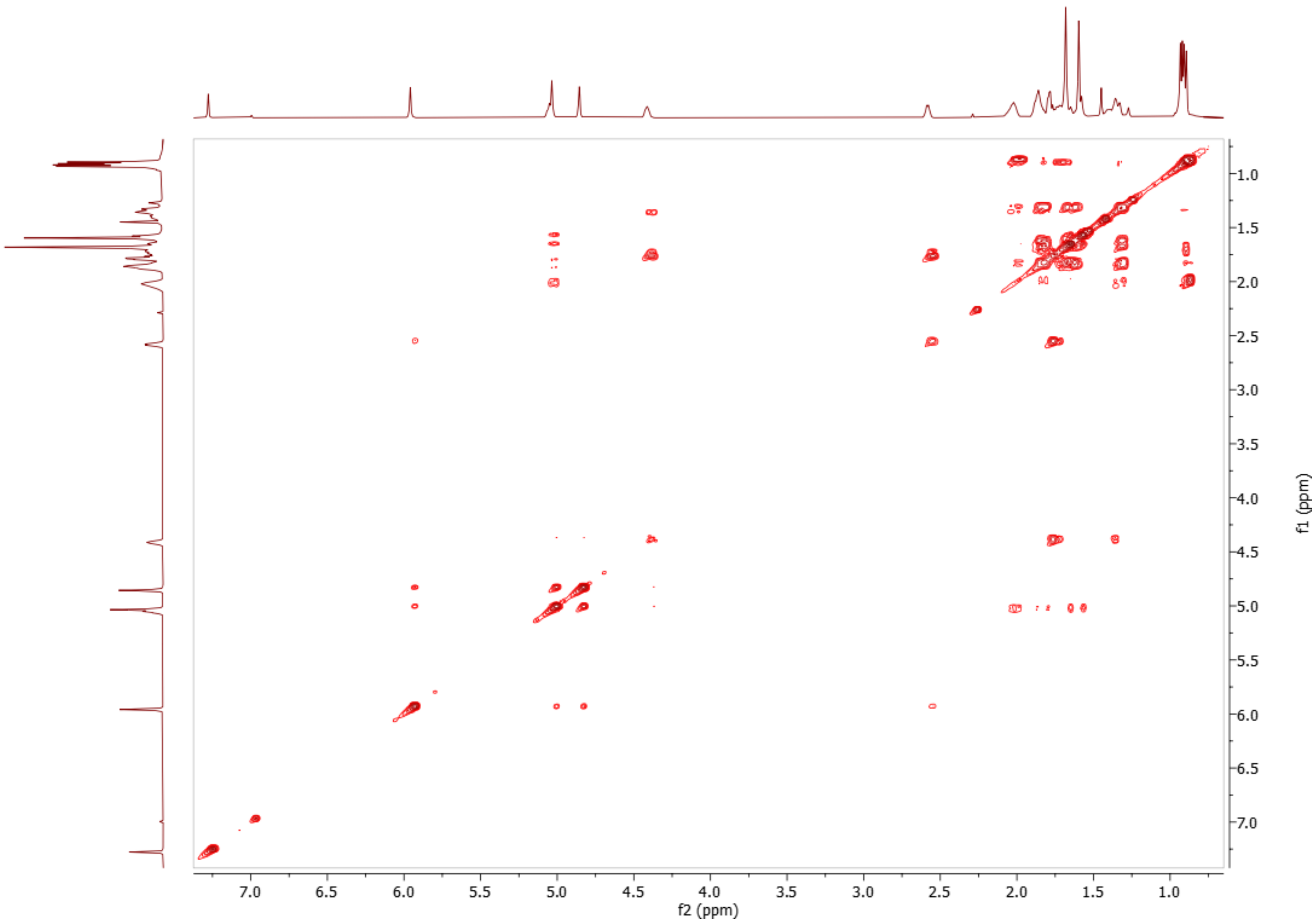

^{1}H-^{1}H COSY spectrum of *epi*-elisabethatrienol (**2b**) measured at 600 MHz in $CDCl_3$.

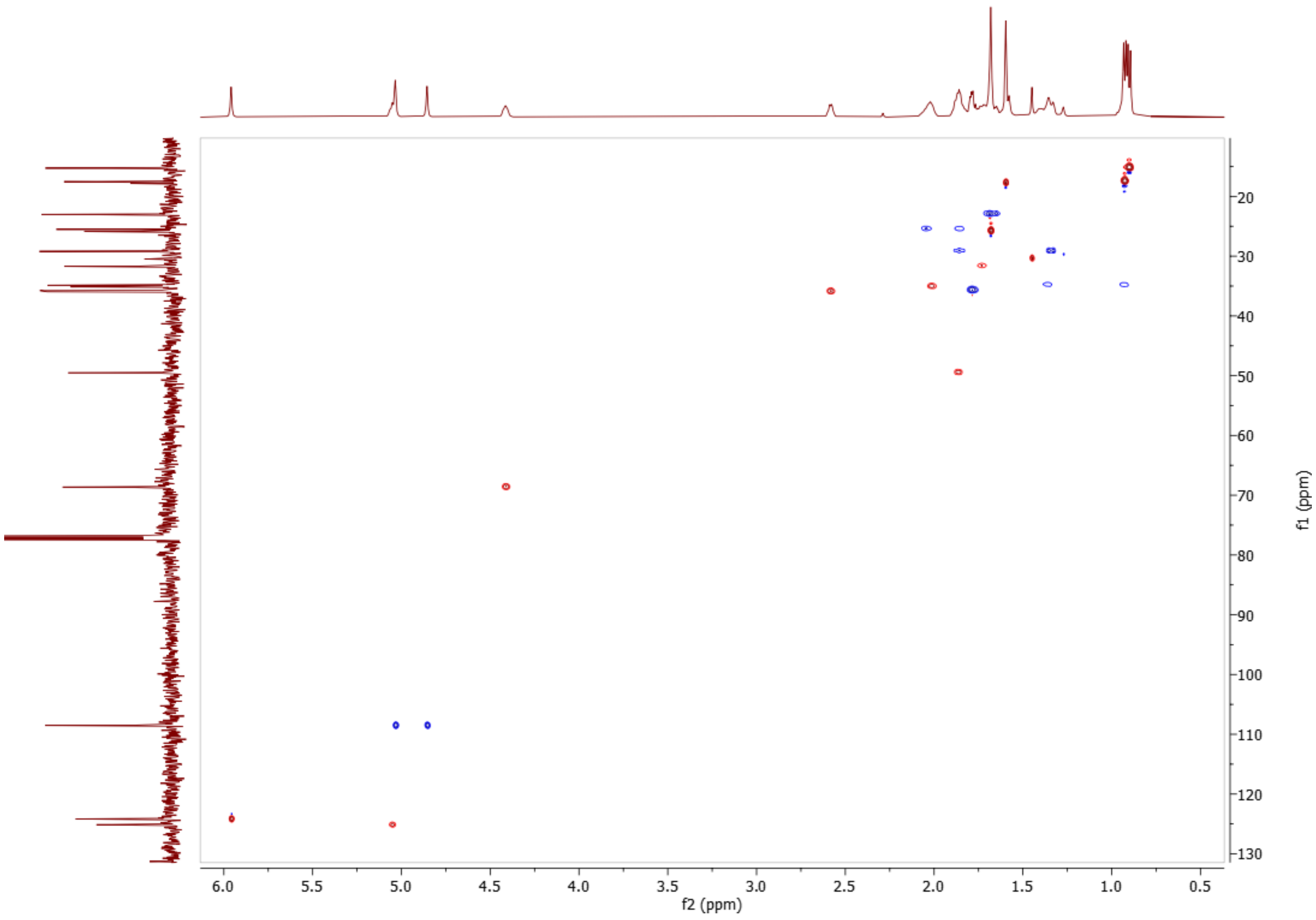

^{1}H-^{13}C HSQC spectrum of *epi*-elisabethatrienol (**2b**) measured at 600 MHz in $CDCl_3$.

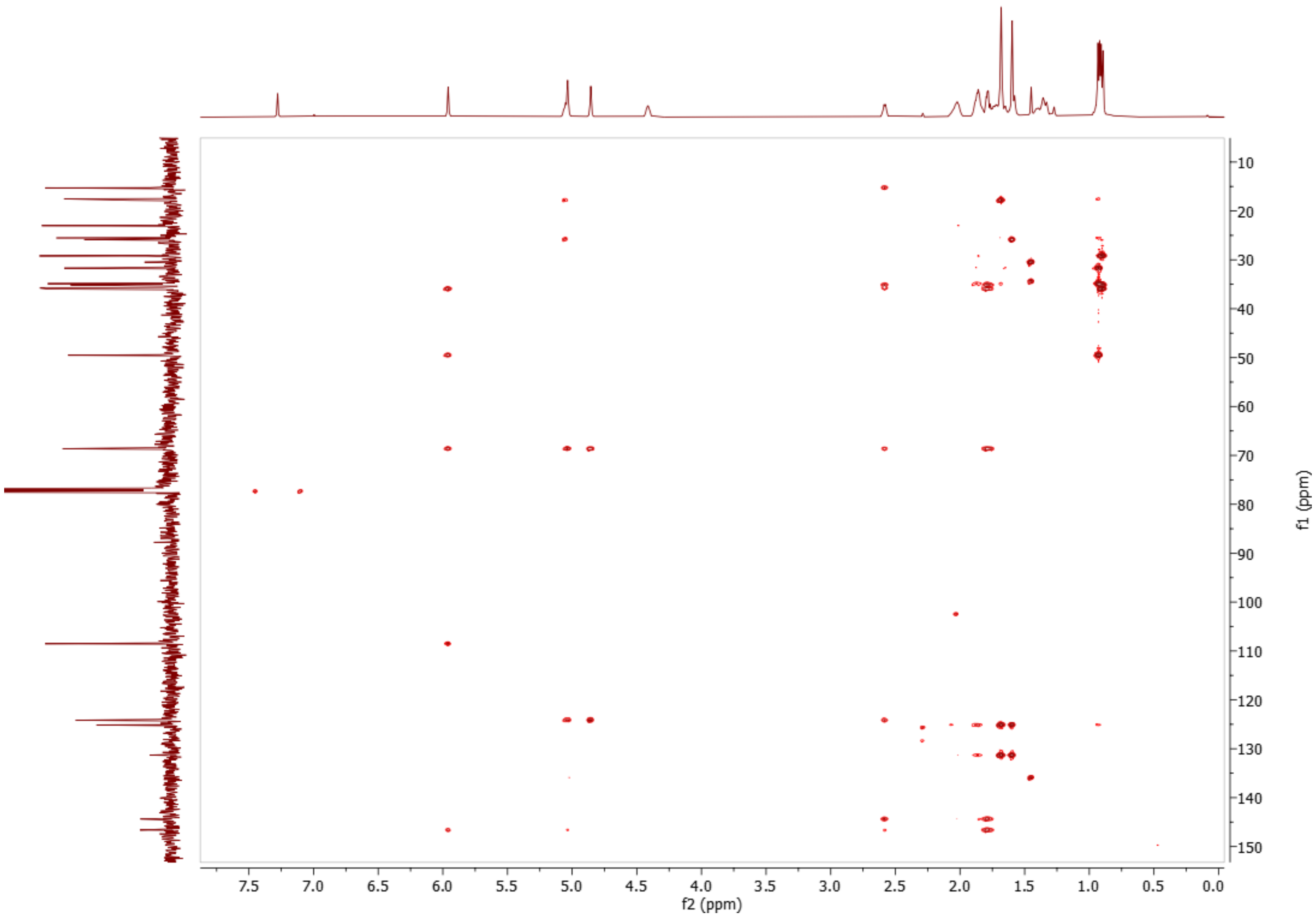

^1H-^{13}C HMBC spectrum of *epi*-elisabethatrienol (**2b**) measured at 600 MHz in $CDCl_3$.

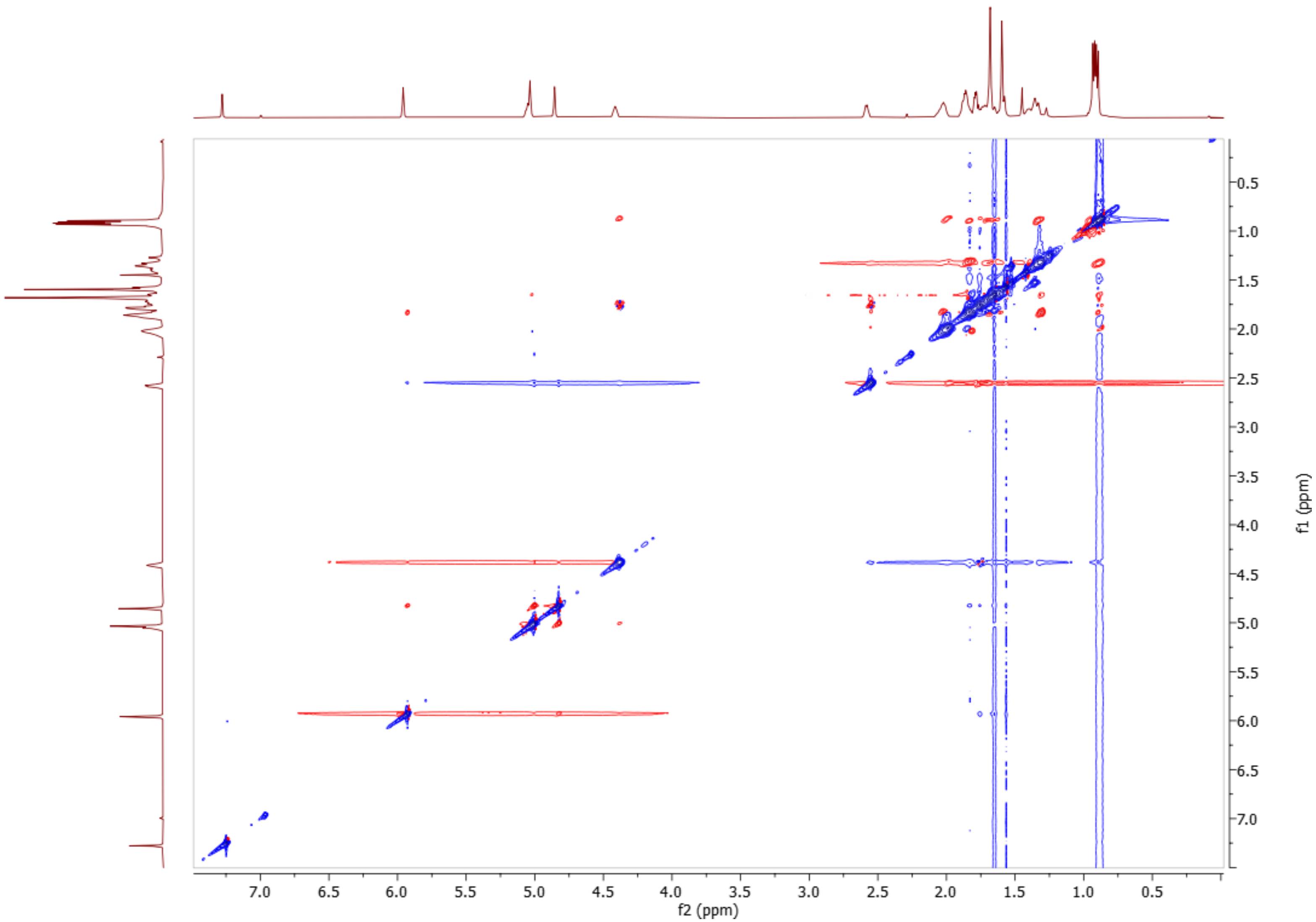

^{1}H-^{1}H NOESY spectrum of *epi*-elisabethatrienol (**2b**) measured at 600 MHz in $CDCl_3$.

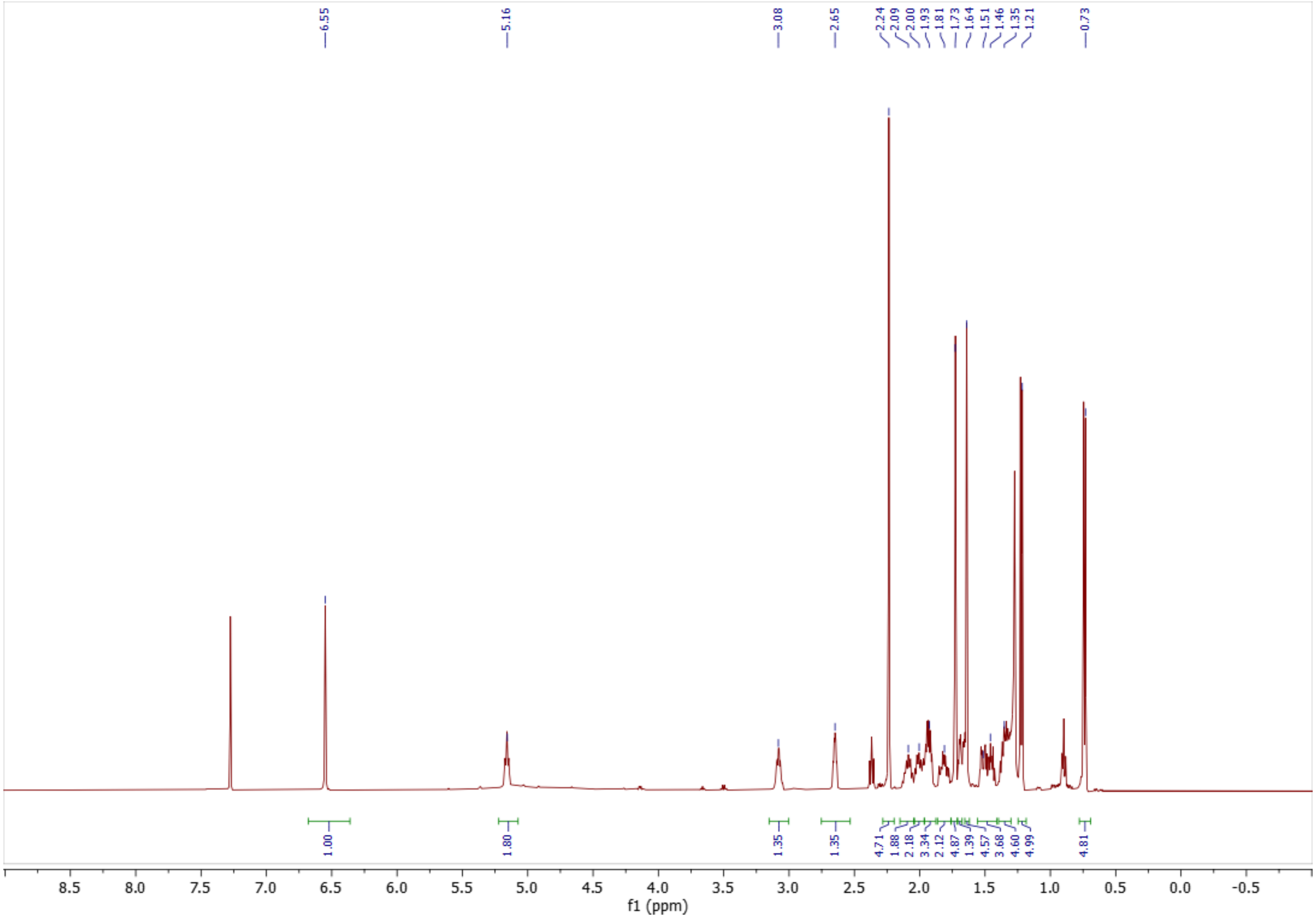

^{1}H-NMR spectrum of 7,8-dihydroxyerogorgiaene (**3**) measured at 500 MHz in $CDCl_3$.

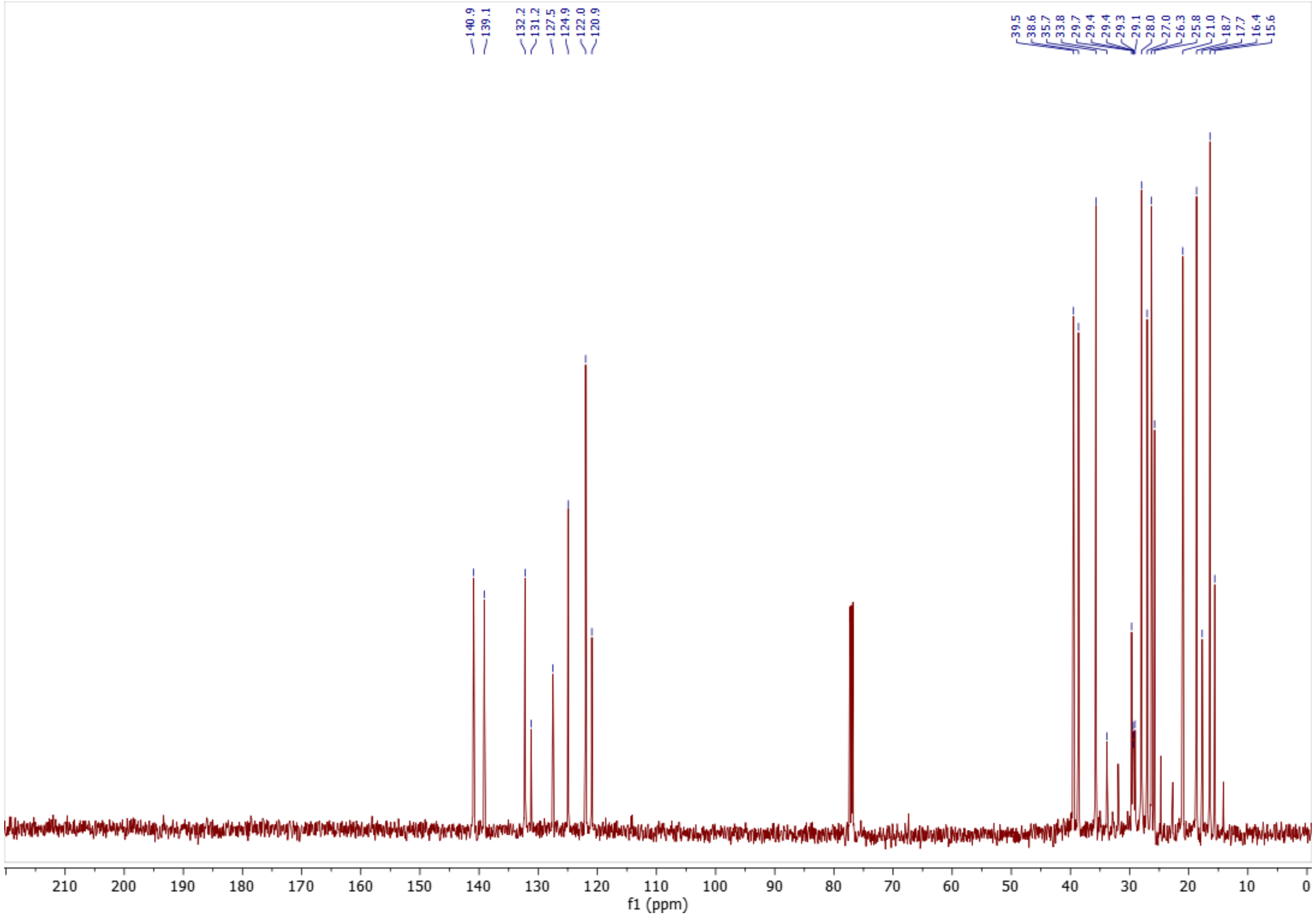

^{13}C-NMR spectrum of 7,8-dihydroxyerogorgiaene (**3**) measured at 125 MHz in $CDCl_3$.

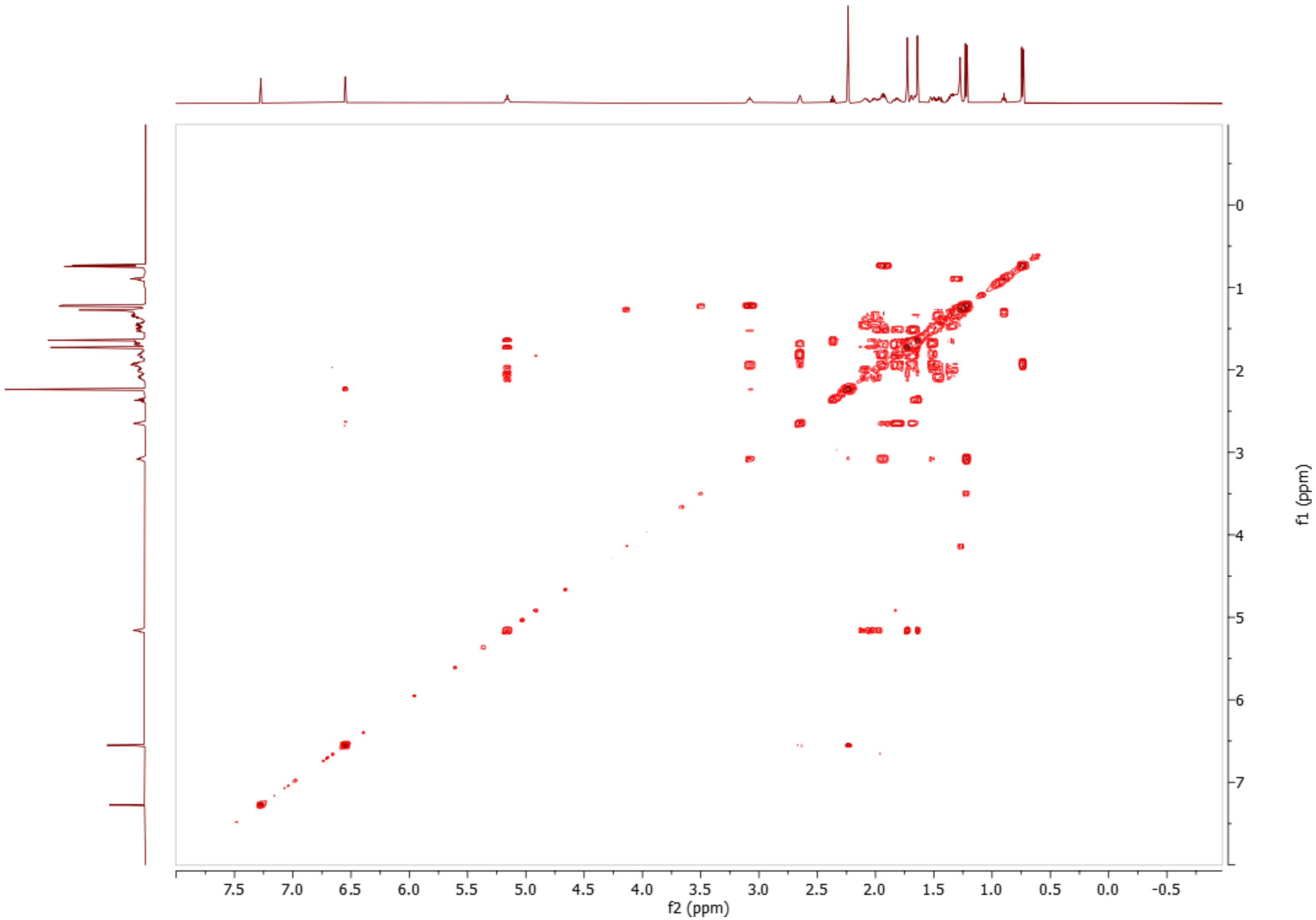

^1H-^1H COSY spectrum of 7,8-dihydroxyerogorgiaene (**3**) measured at 500 MHz in $CDCl_3$.

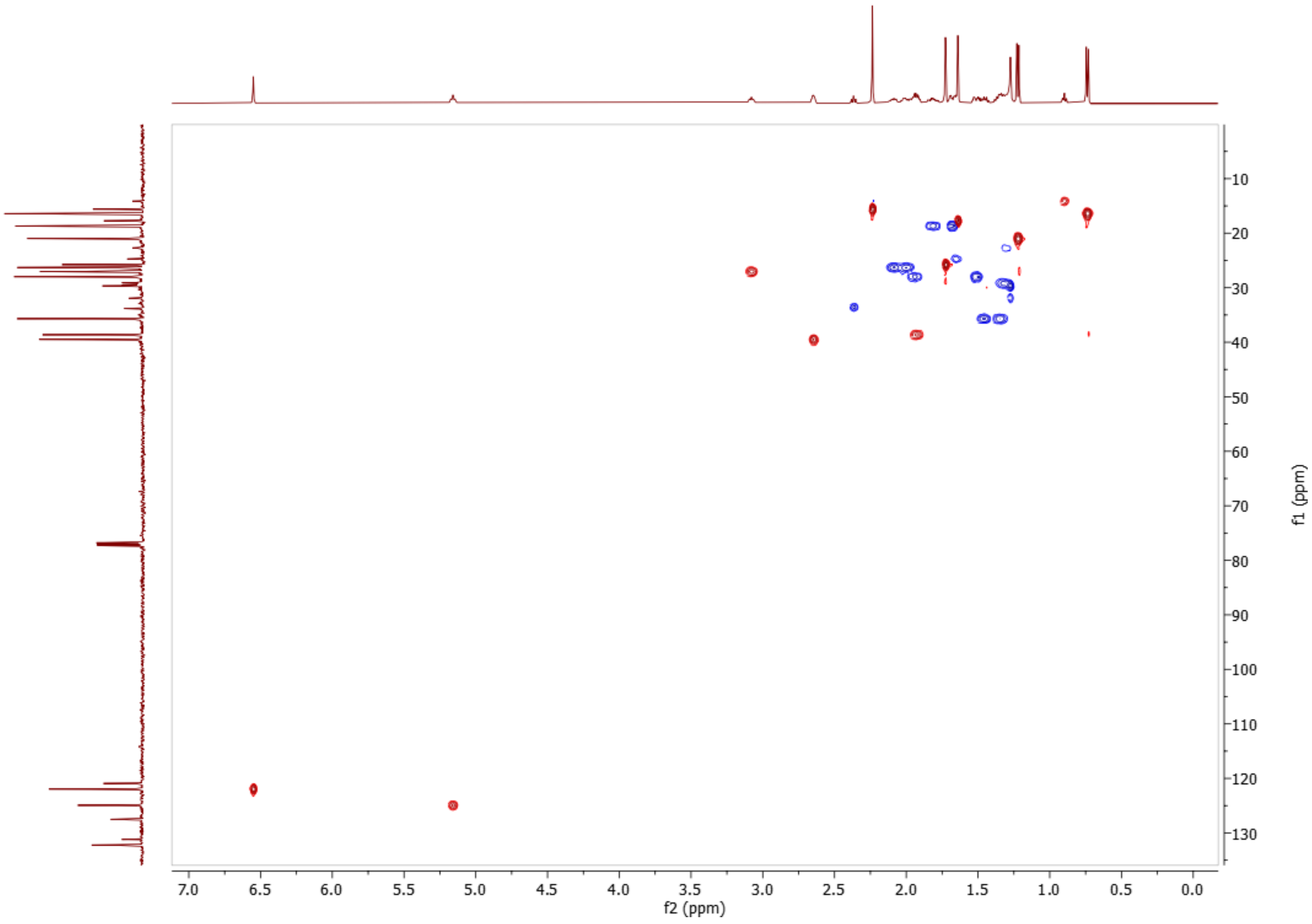

^{1}H-^{13}C HSQC spectrum of 7,8-dihydroxyerogorgiaene (**3**) measured at 500 MHz in $CDCl_3$.

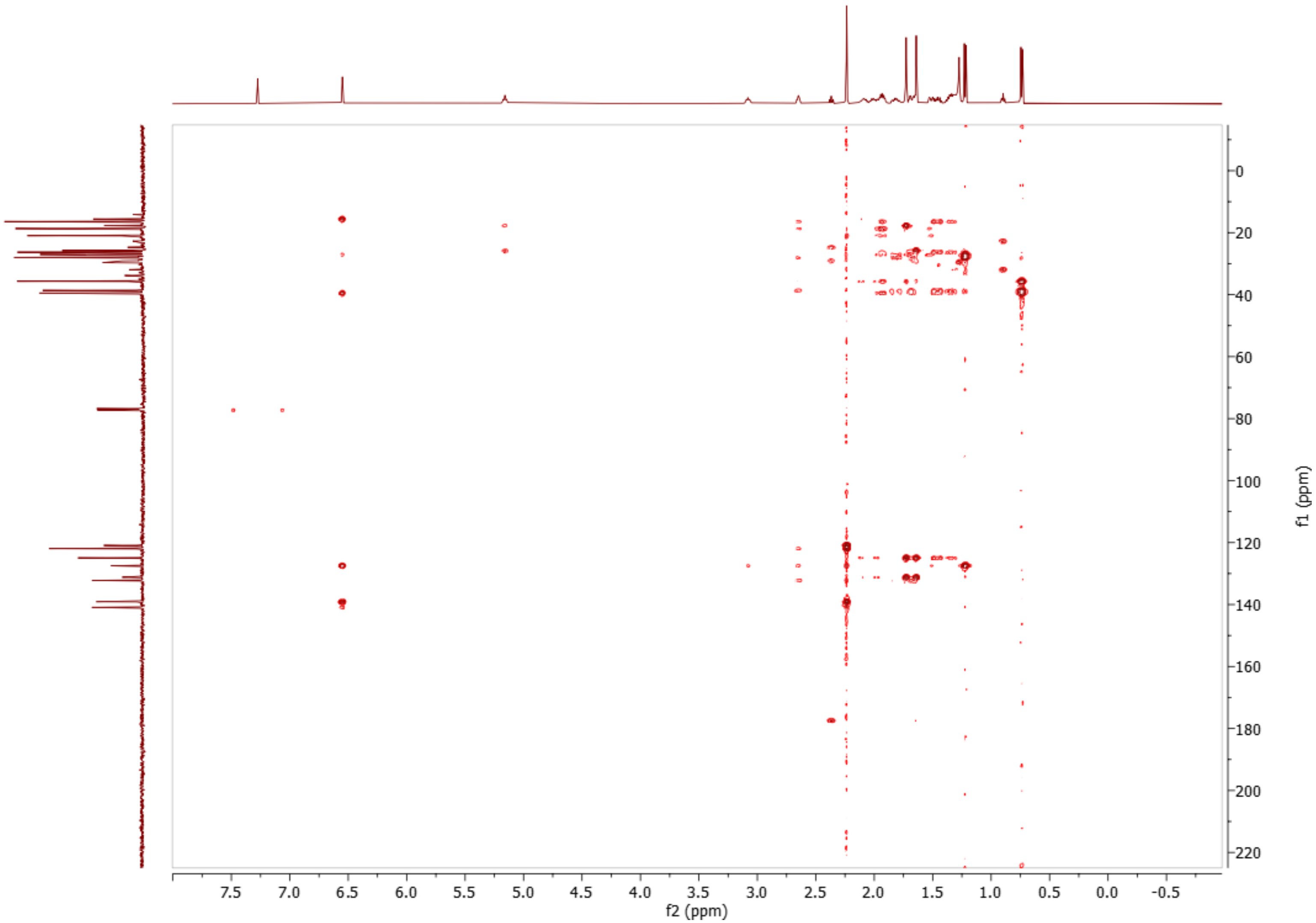

^{1}H-^{13}C HMBC spectrum of 7,8-dihydroxyerogorgiaene (**3**) measured at 500 MHz in $CDCl_3$.

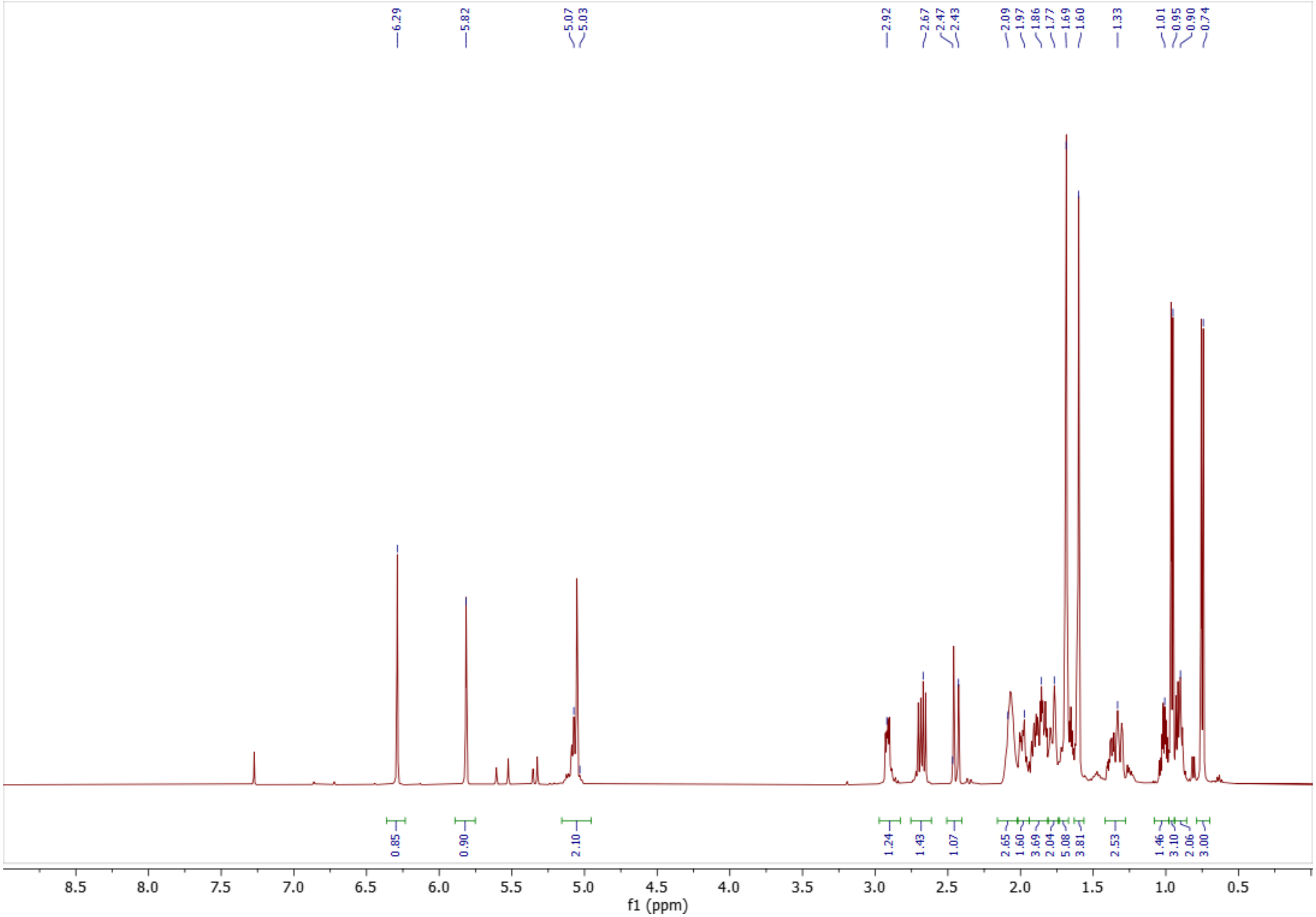

^{1}H-NMR spectrum of ketone intermediate **4** measured at 500 MHz in $CDCl_3$.

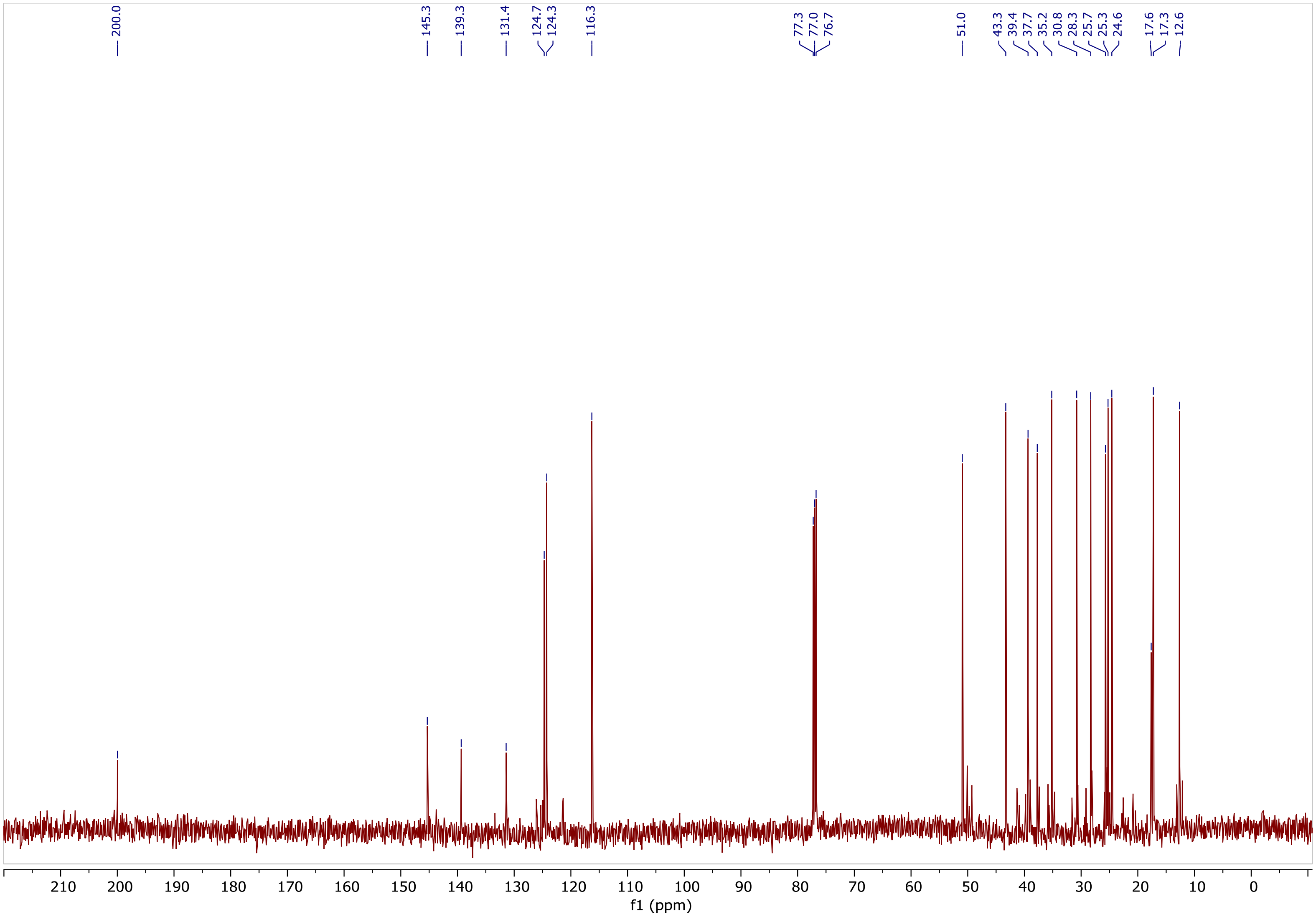

^{13}C-NMR spectrum of ketone intermediate **4** measured at 125 MHz in $CDCl_3$.

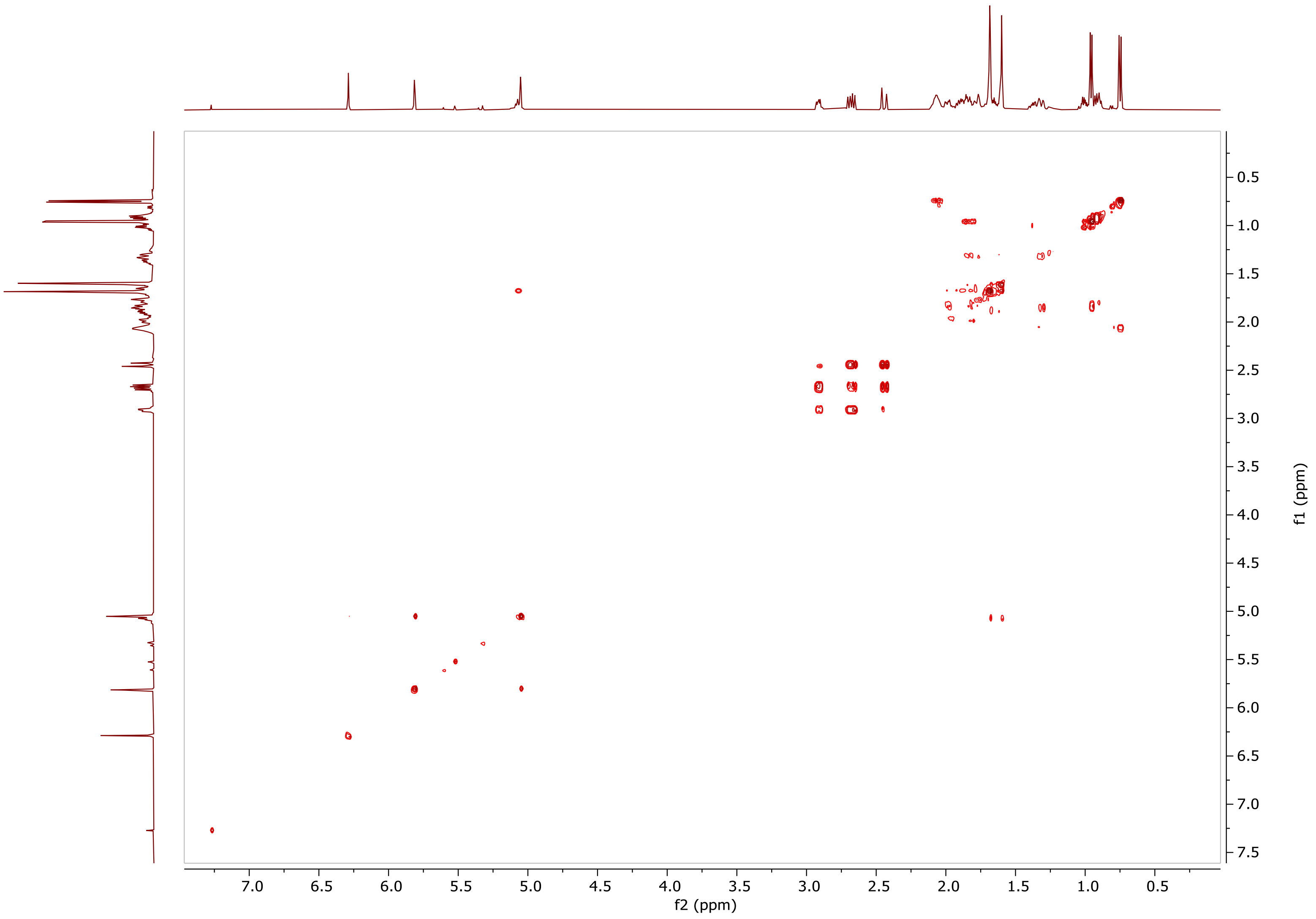

^{1}H-^{1}H COSY NMR spectrum of ketone intermediate **4** measured at 500 MHz in $CDCl_3$.

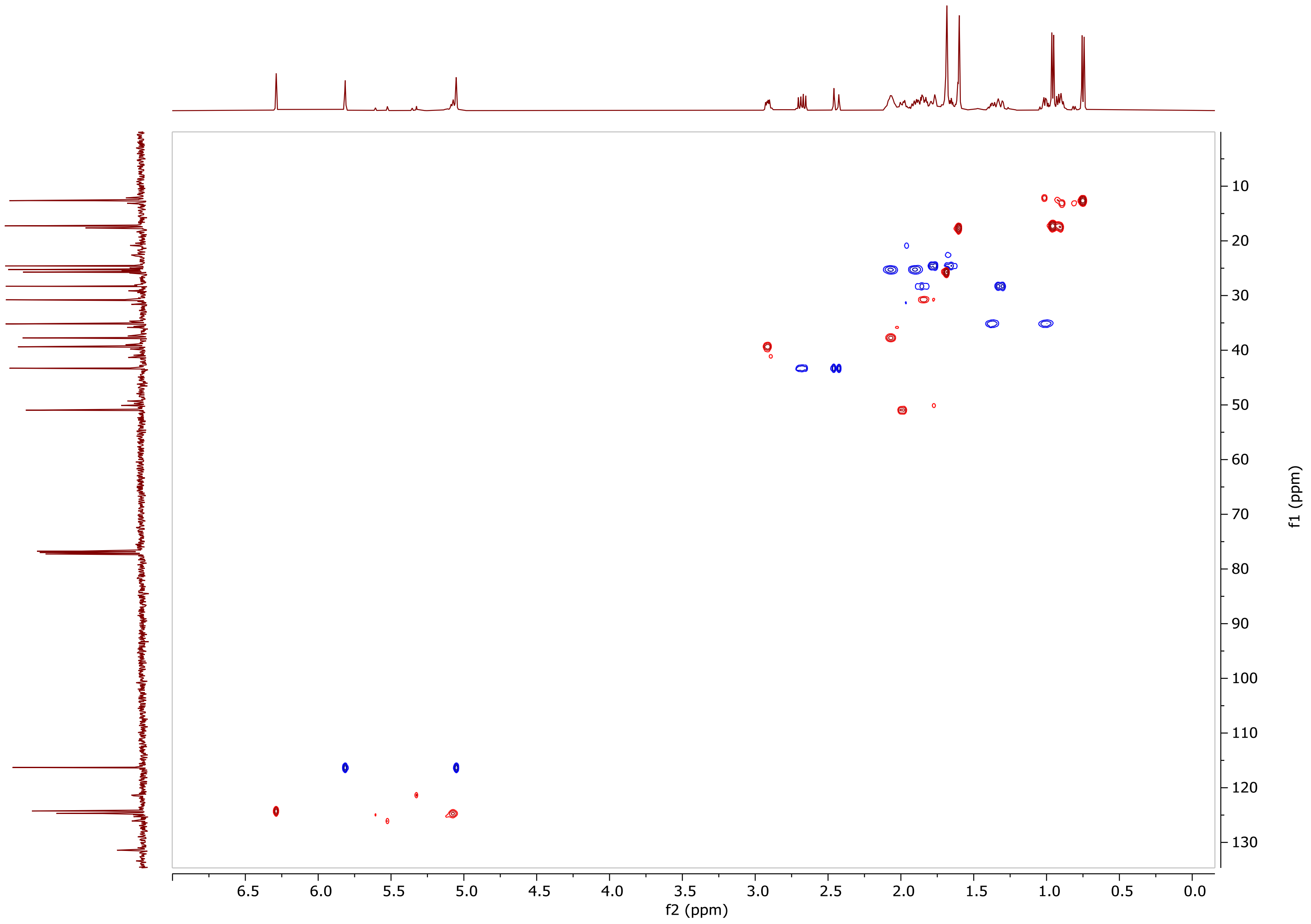

^{1}H-^{13}C HSQC NMR spectrum of ketone intermediate **4** measured at 500 MHz in $CDCl_3$.

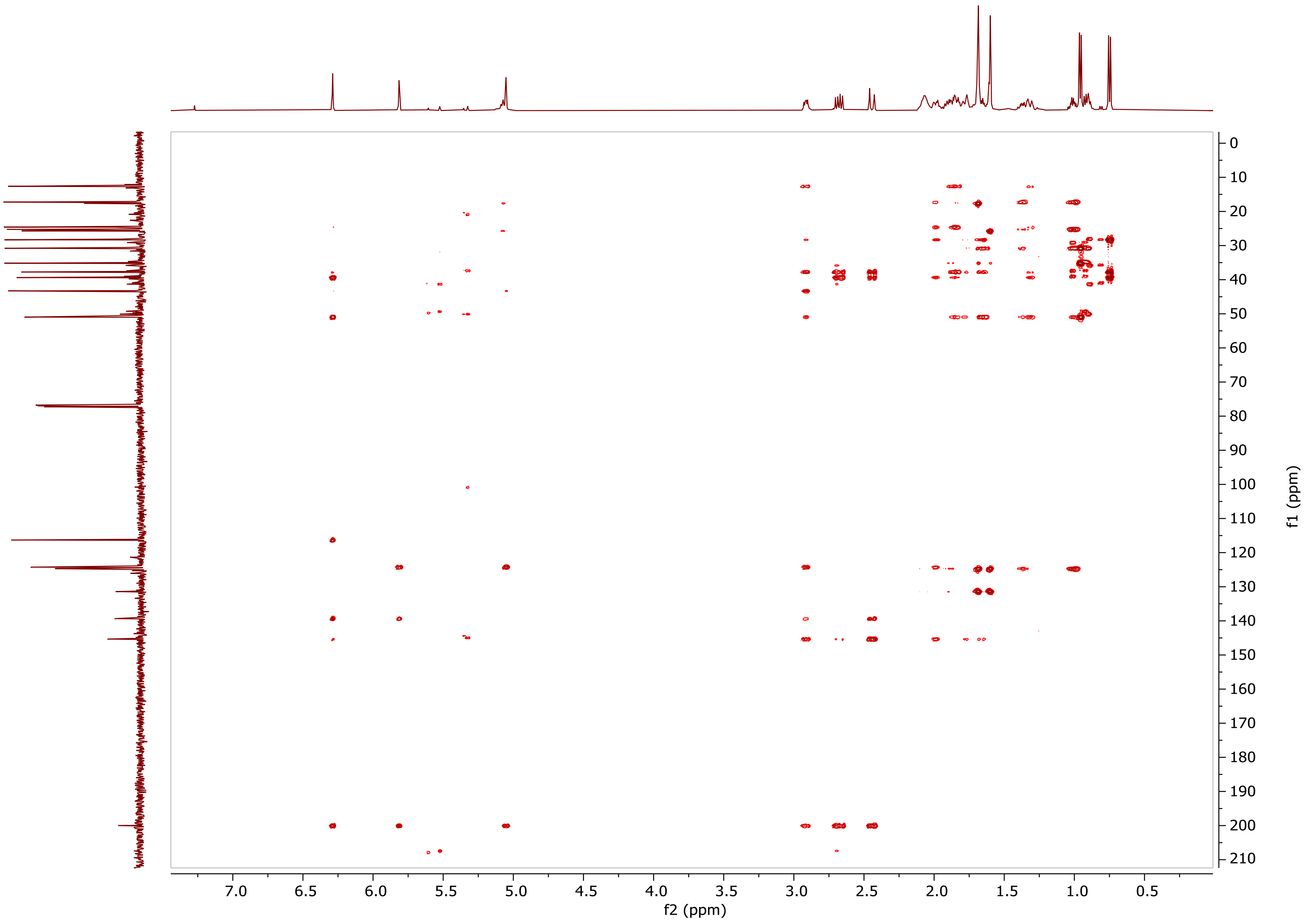

^{1}H-^{13}C HMBC NMR spectrum of ketone intermediate **4** measured at 500 MHz in $CDCl_3$.

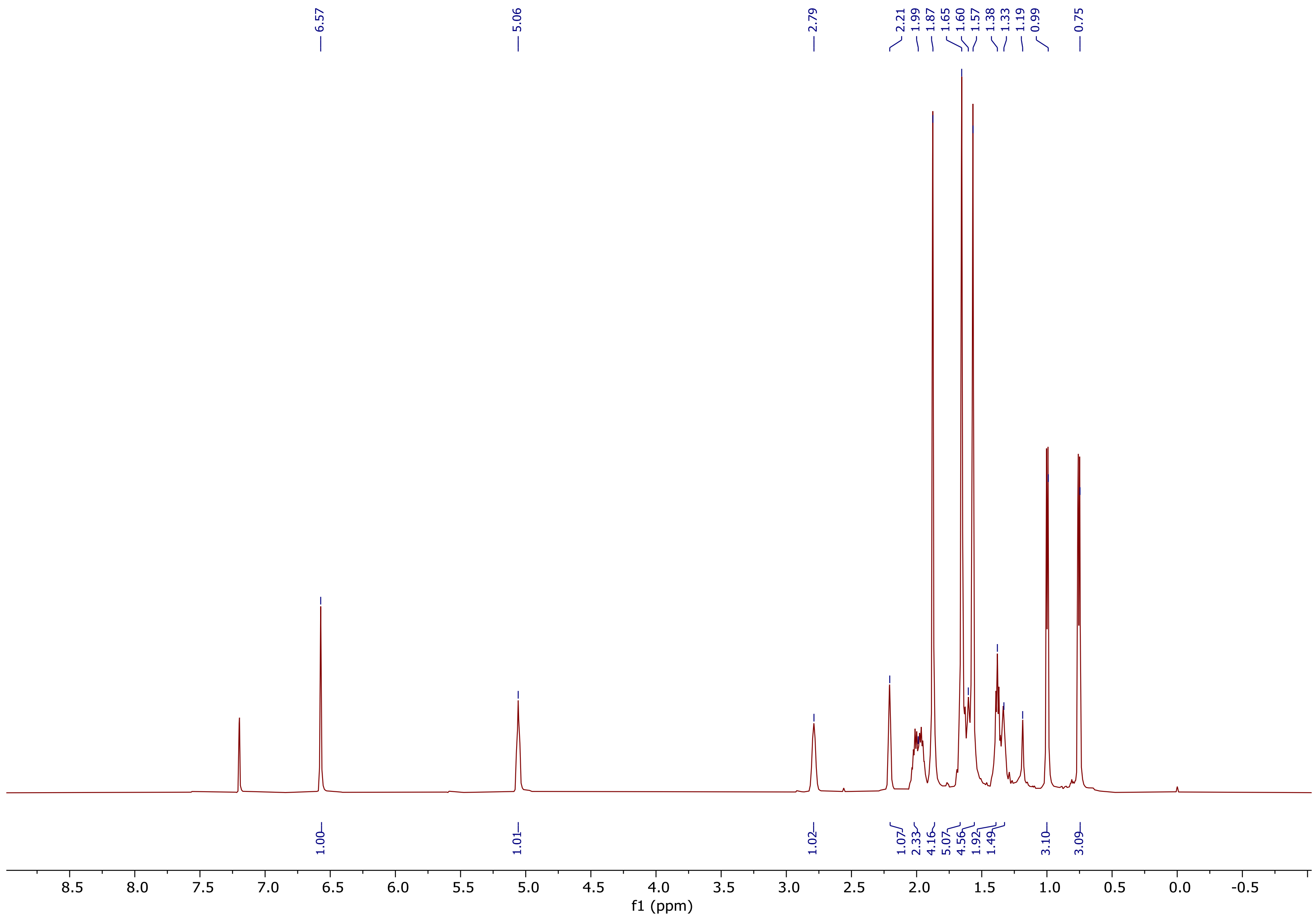

^{1}H-NMR spectrum of quinone **5** measured at 500 MHz in $CDCl_3$.

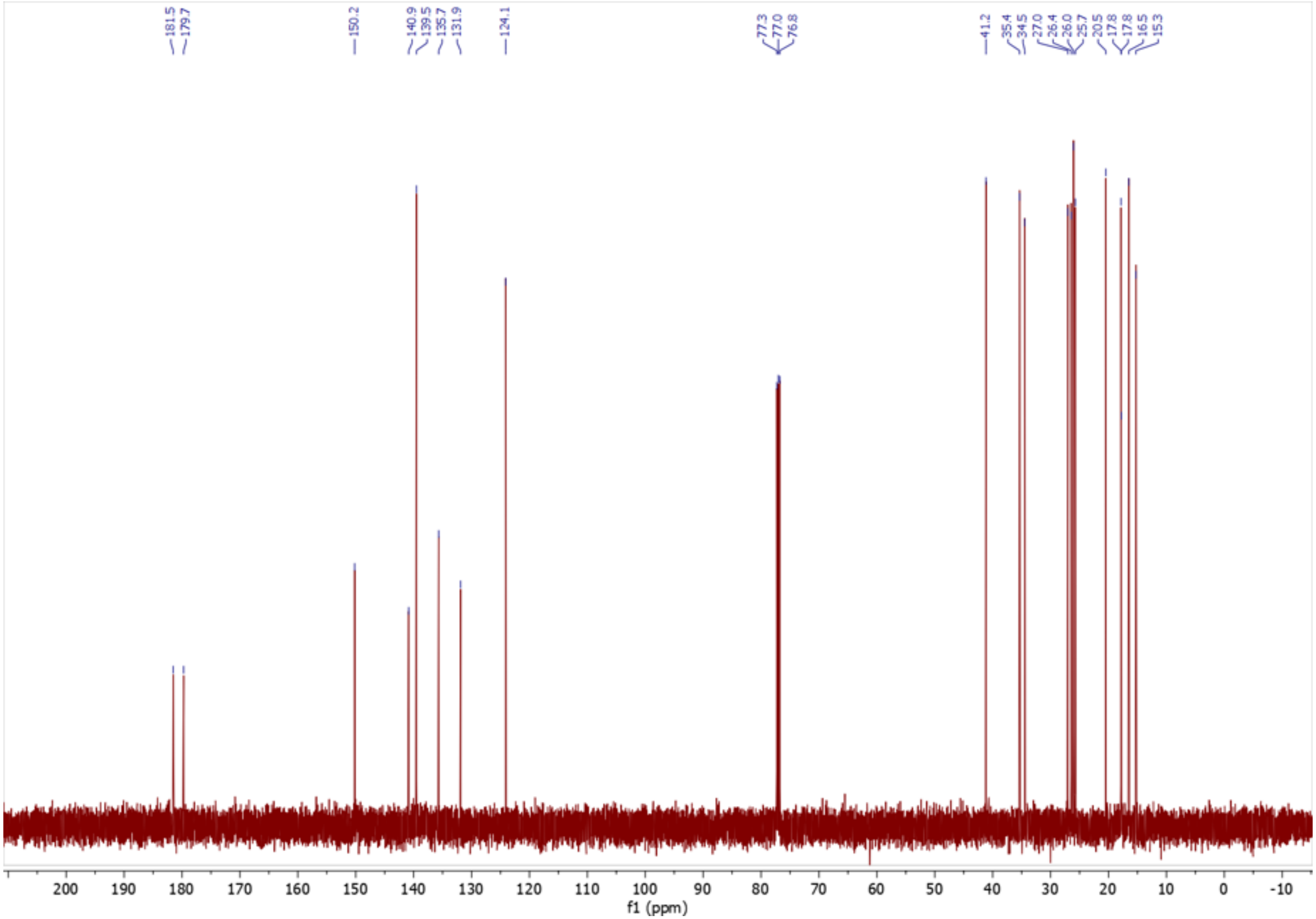

^{13}C-NMR spectrum of quinone **5** measured at 125 MHz in $CDCl_3$.

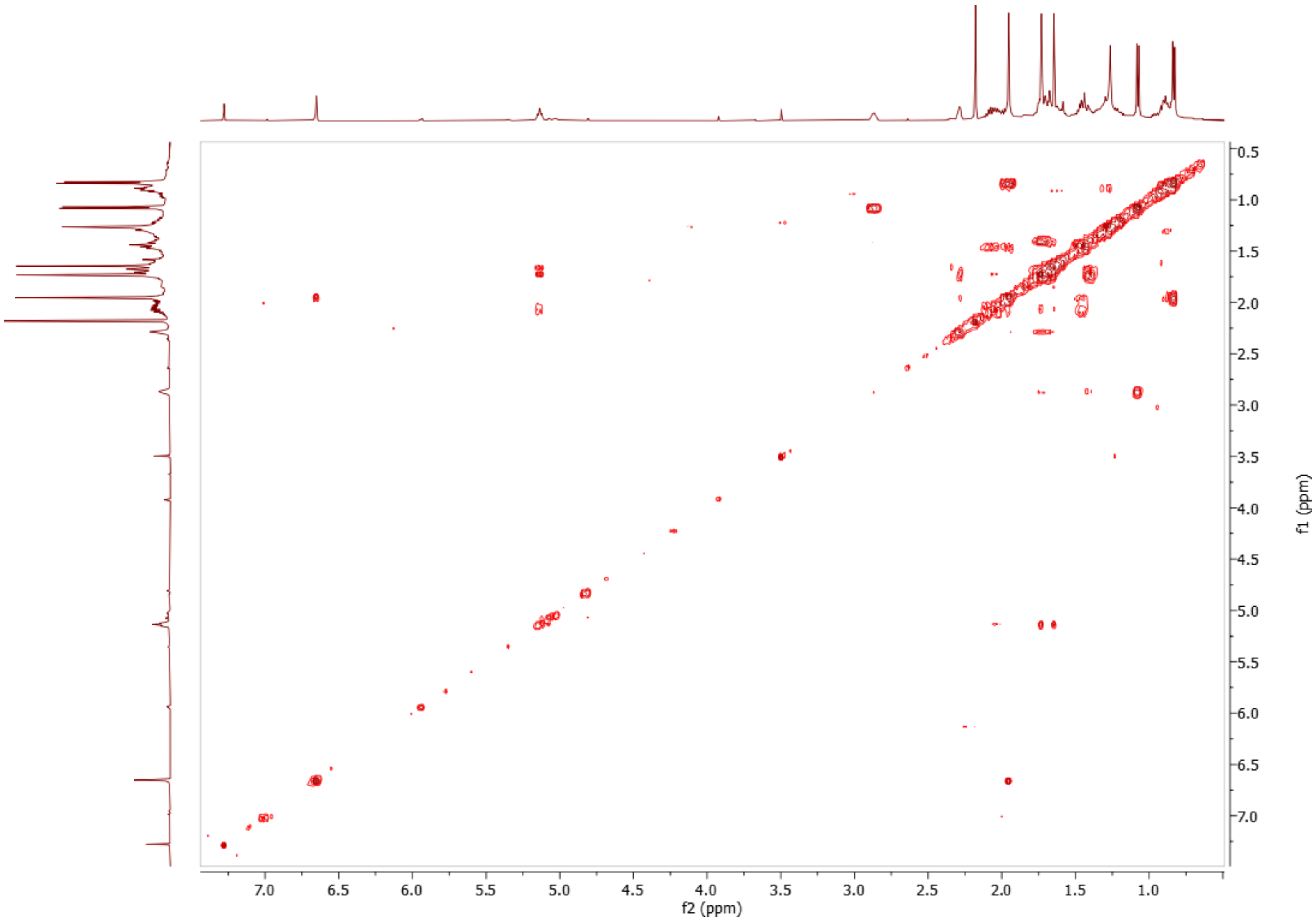

^{1}H-^{1}H COSY spectrum of quinone **5** measured at 500 MHz in $CDCl_3$.

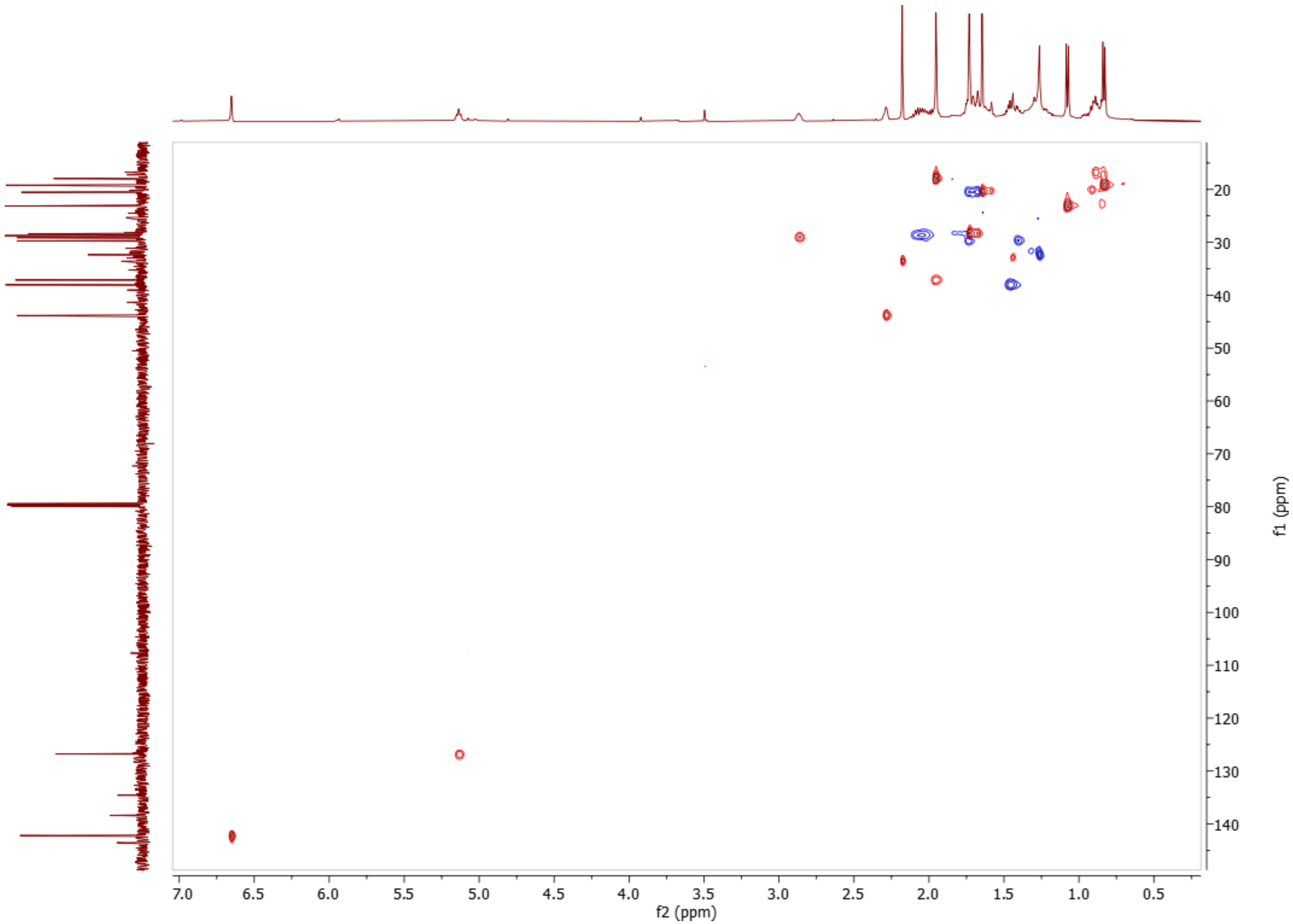

^{1}H-^{13}C HSQC spectrum of quinone **5** measured at 500 MHz in $CDCl_3$.

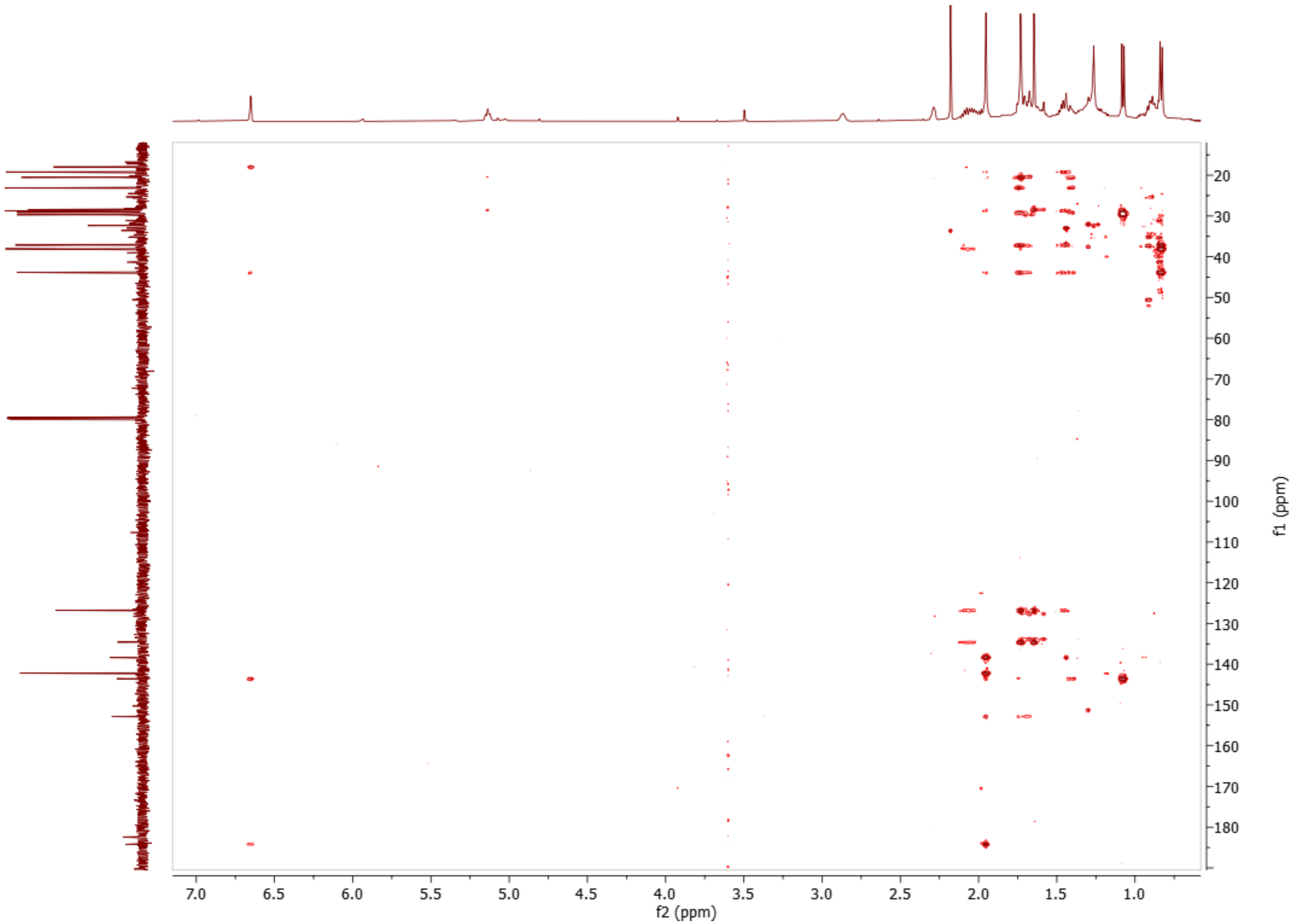

^{1}H-^{13}C HMBC spectrum of quinone **5** measured at 500 MHz in $CDCl_3$.

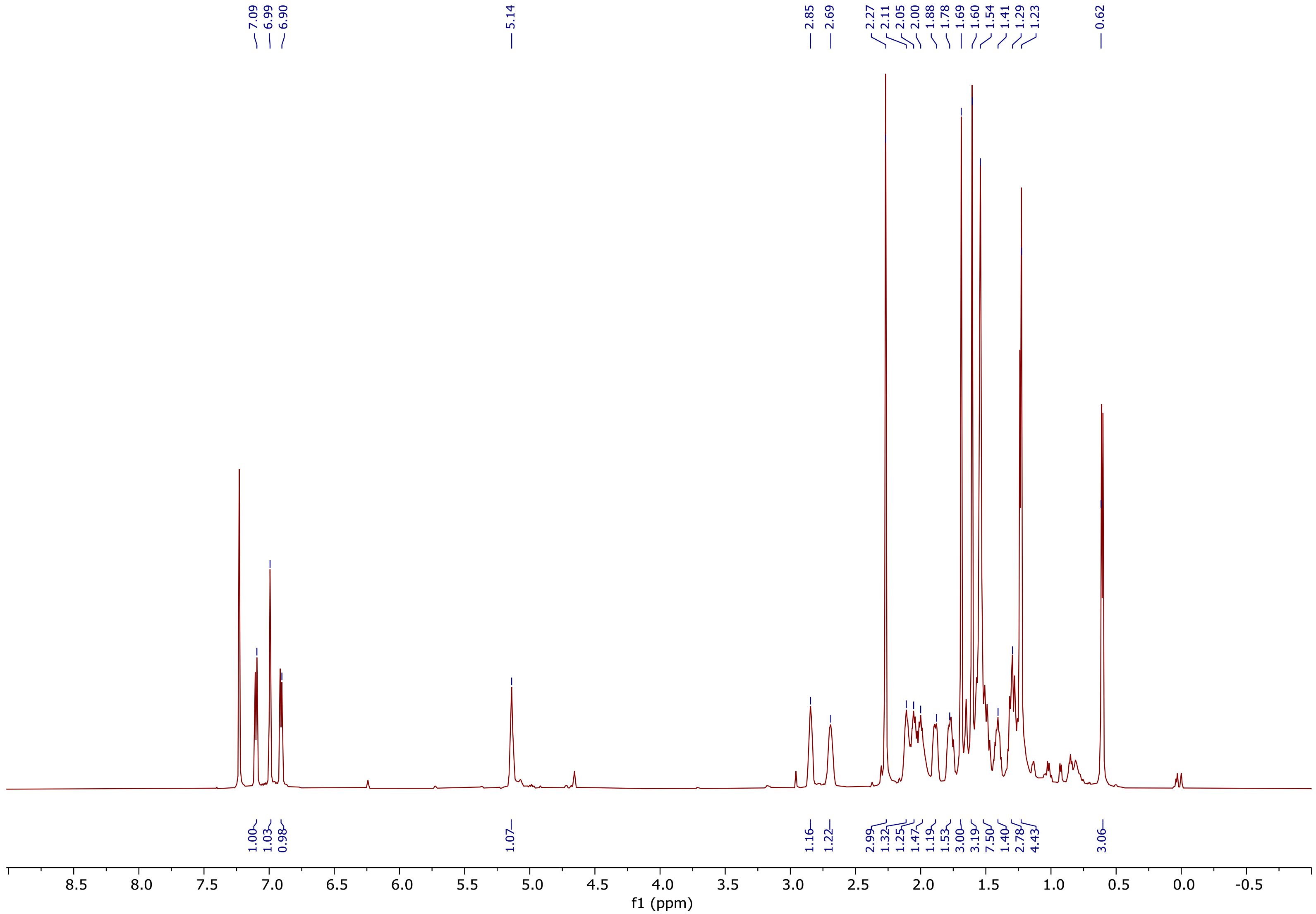

^{1}H-NMR spectrum of erogorgiaene (**6**) measured at 500 MHz in $CDCl_3$.

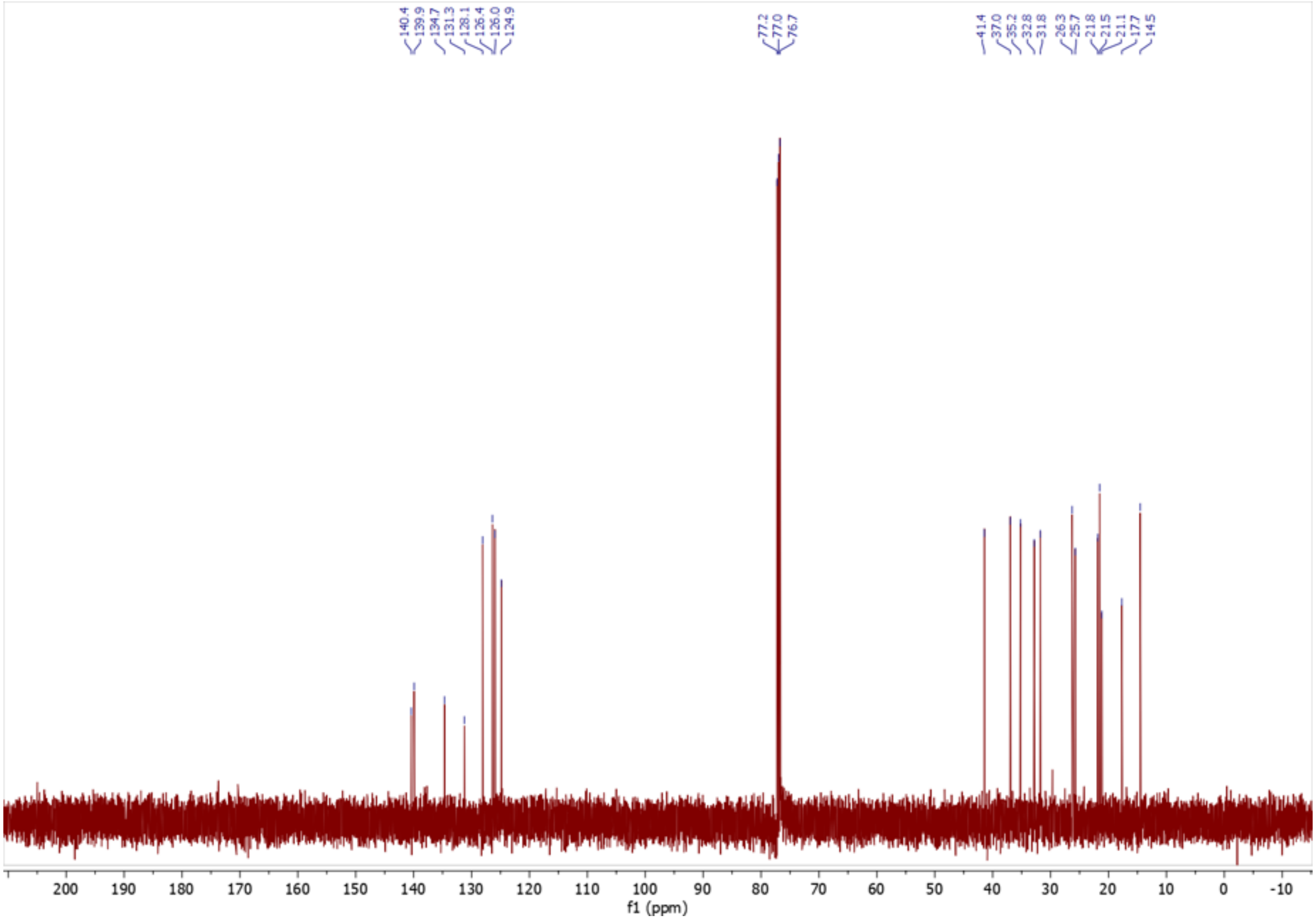

^{13}C-NMR spectrum of erogorgiaene (**6**) measured at 125 MHz in $CDCl_3$.

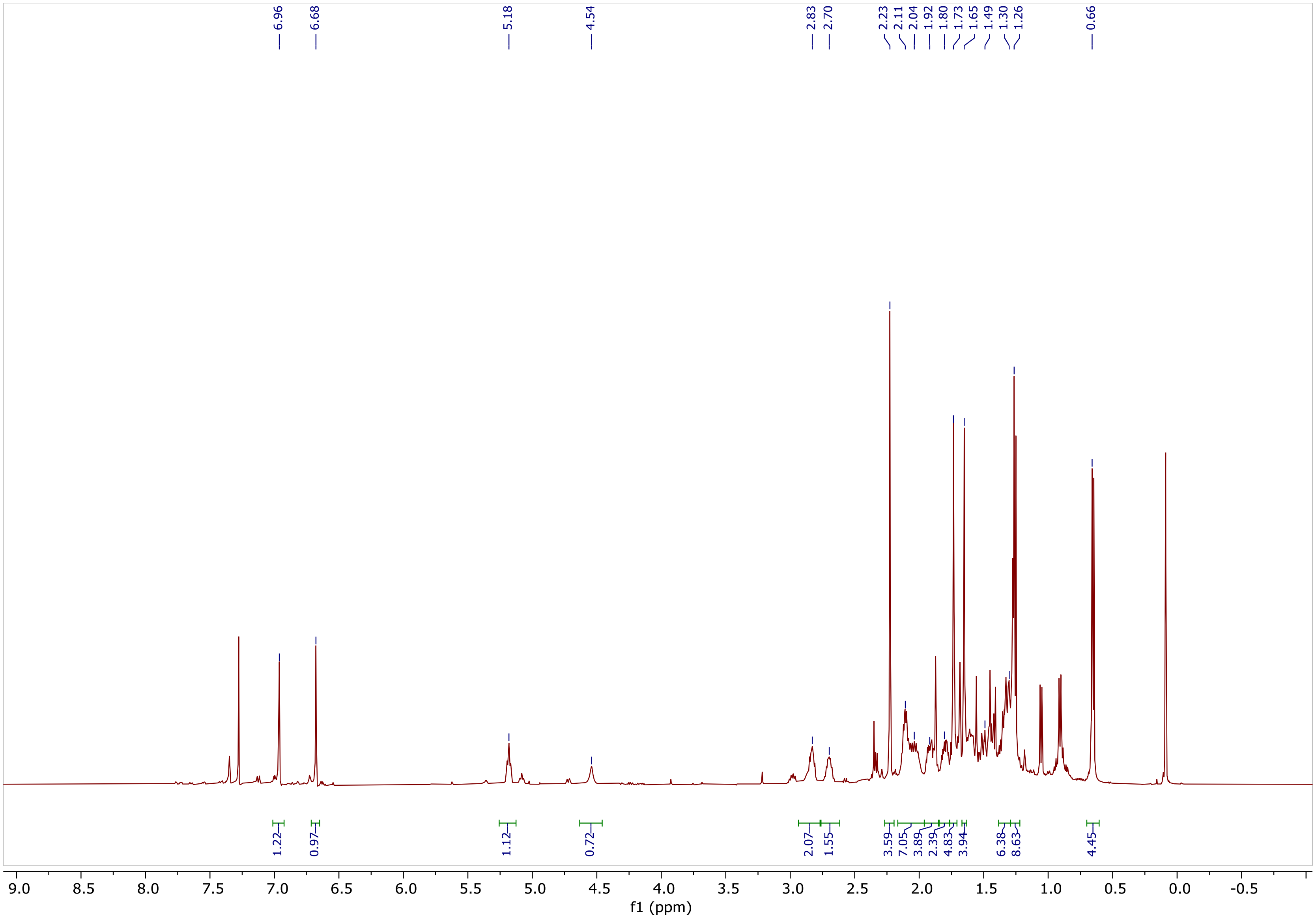

^{1}H NMR spectrum of 7-hydroxyerogorgiaenene (**7**) measured at 500 MHz in $CDCl_3$.

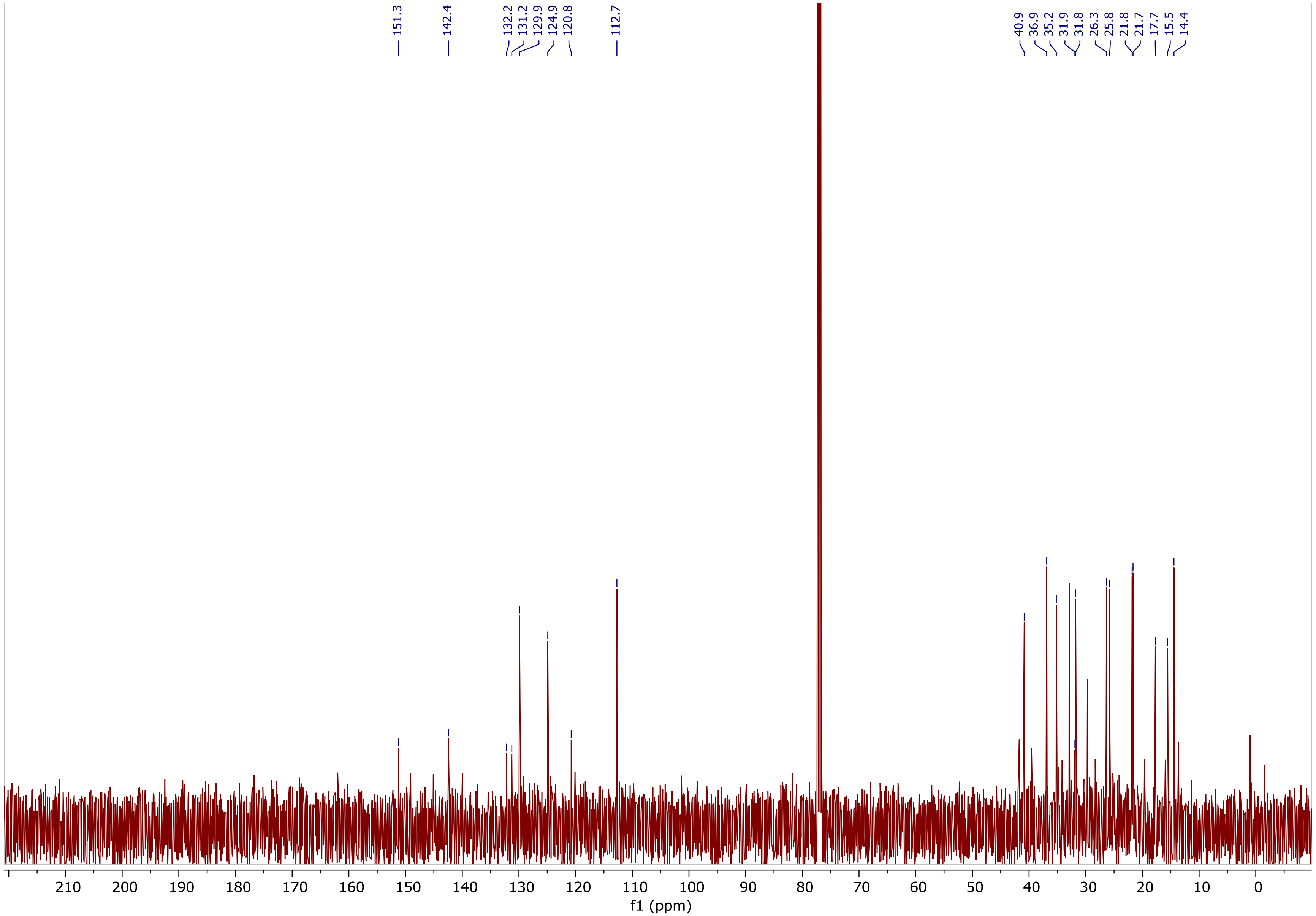

^{13}C NMR spectrum of 7-hydroxyerogorgiaenene (**7**) measured at 125 MHz in $CDCl_3$.

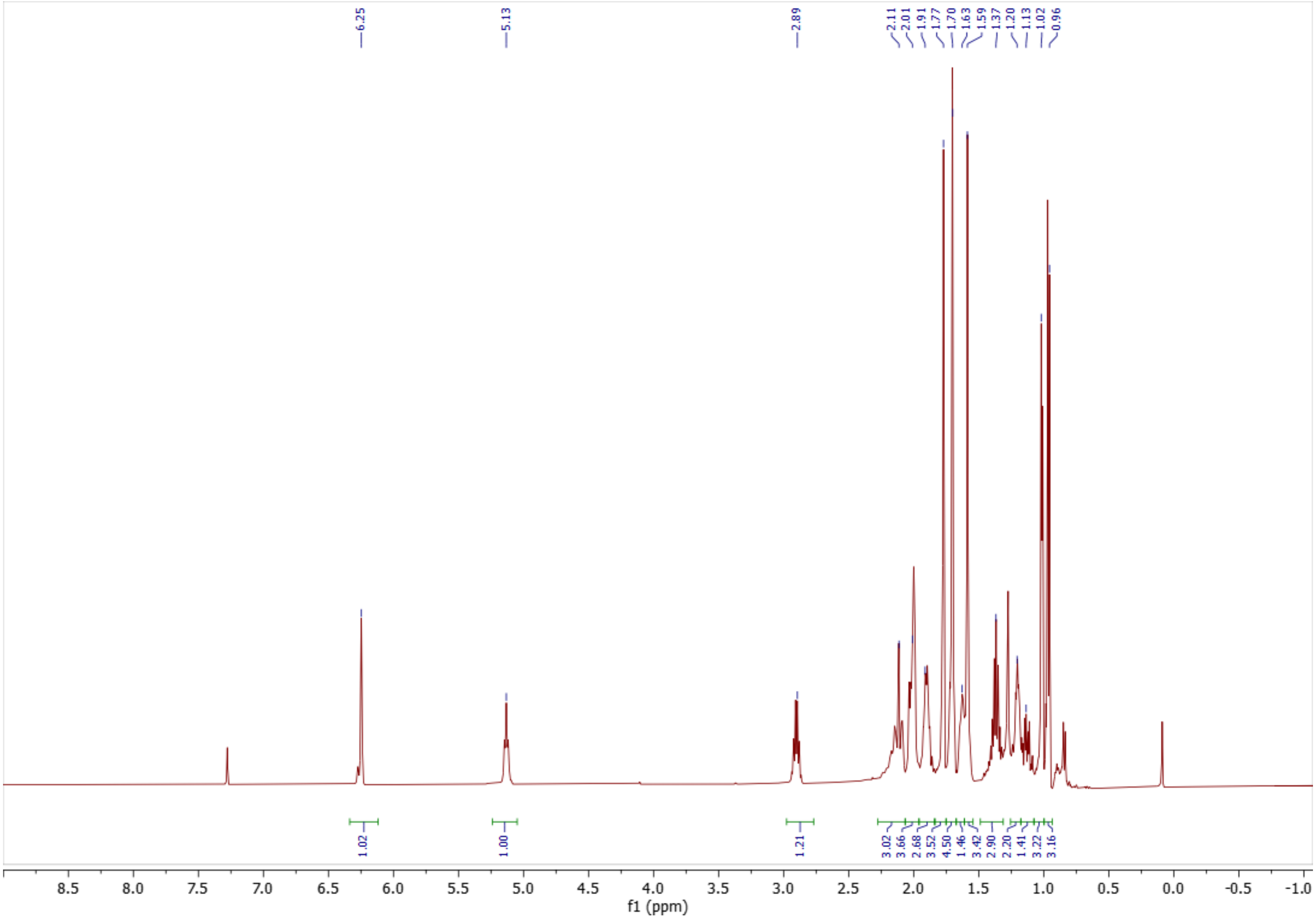

^{1}H NMR spectrum of isoelisabethatriene C (**S1**) measured at 500 MHz in $CDCl_3$.

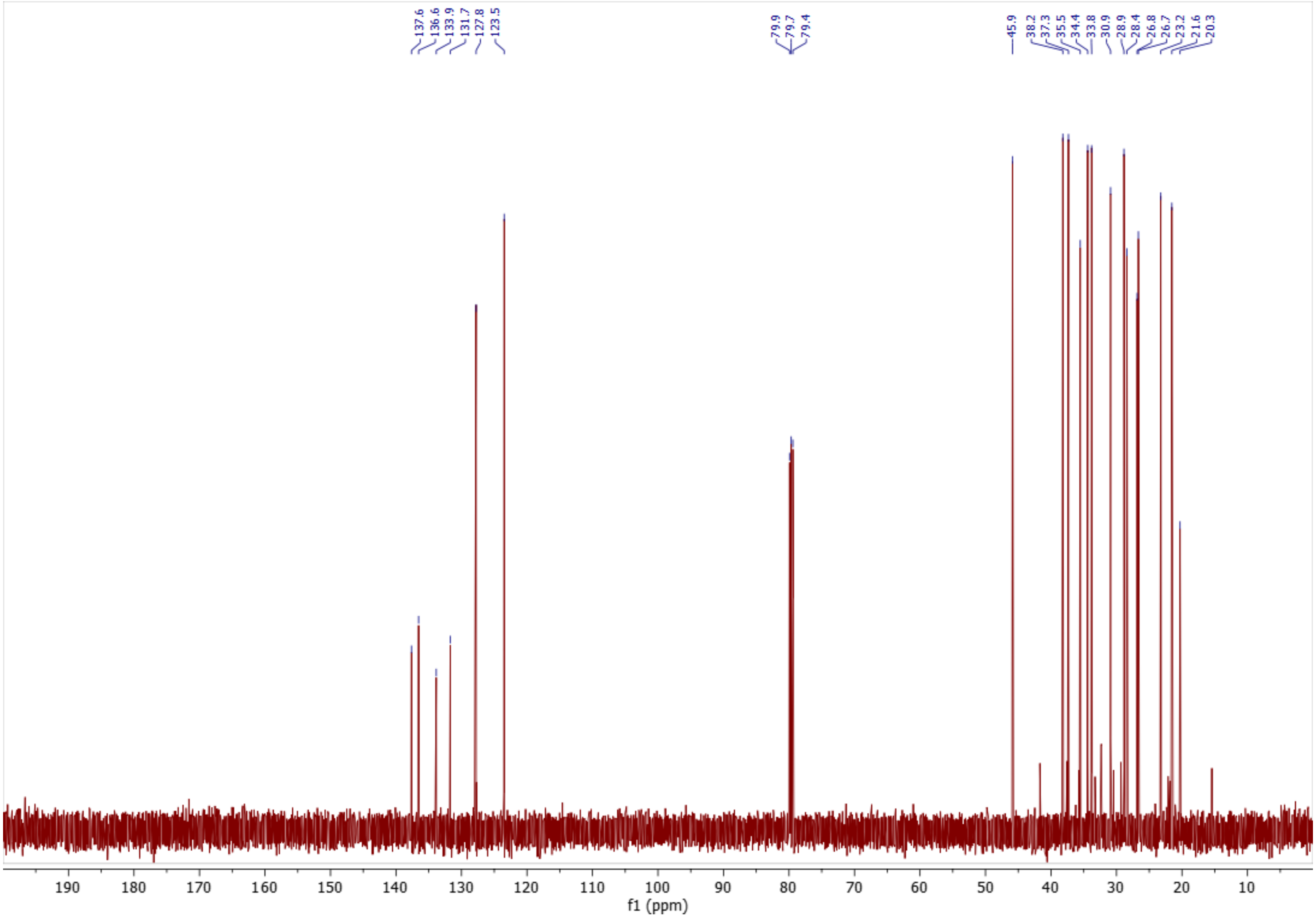

^{13}C NMR spectrum of isoelisabethatriene C (**S1**) measured at 125 MHz in $CDCl_3$.

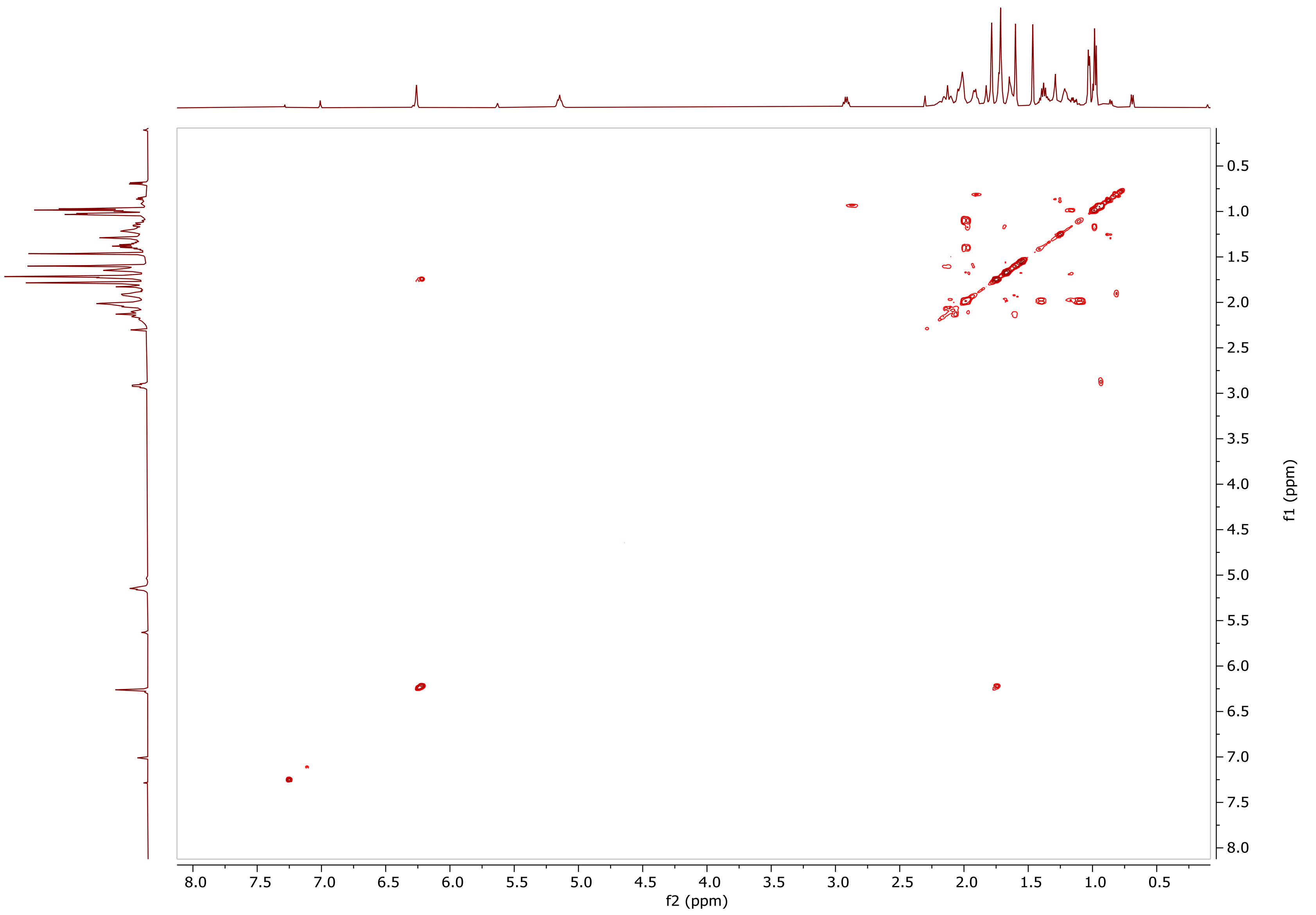

^{1}H-^{1}H COSY NMR spectrum of isoelisabethatriene C (**S1**) measured at 500 MHz in $CDCl_3$.

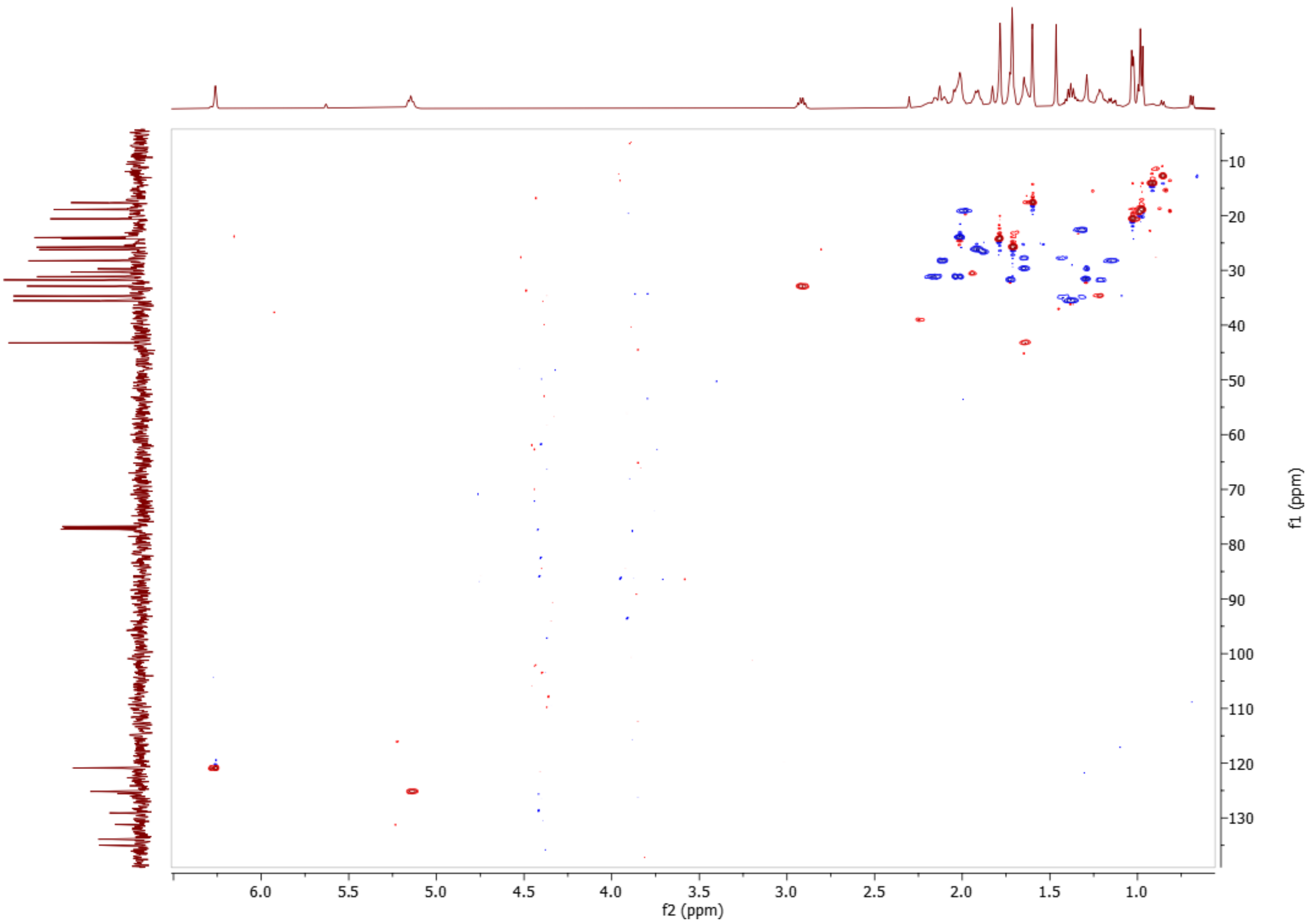

^{1}H-^{13}C HSQC NMR spectrum of isoelisabethatriene C (**S1**) measured at 500 MHz in $CDCl_3$.

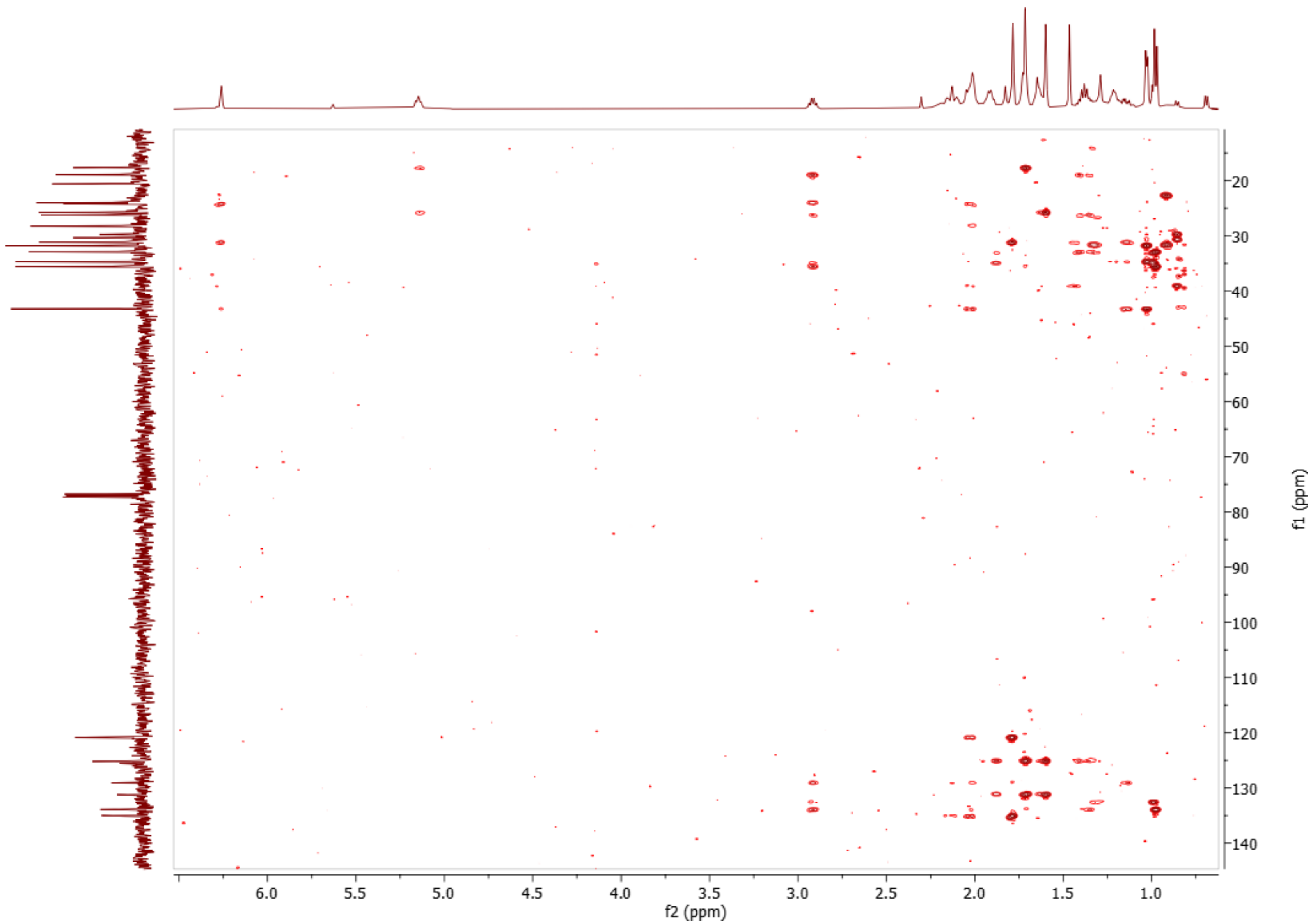

^1H-^{13}C HMBC NMR spectrum of isoelisabethatriene C (**S1**) measured at 500 MHz in $CDCl_3$.

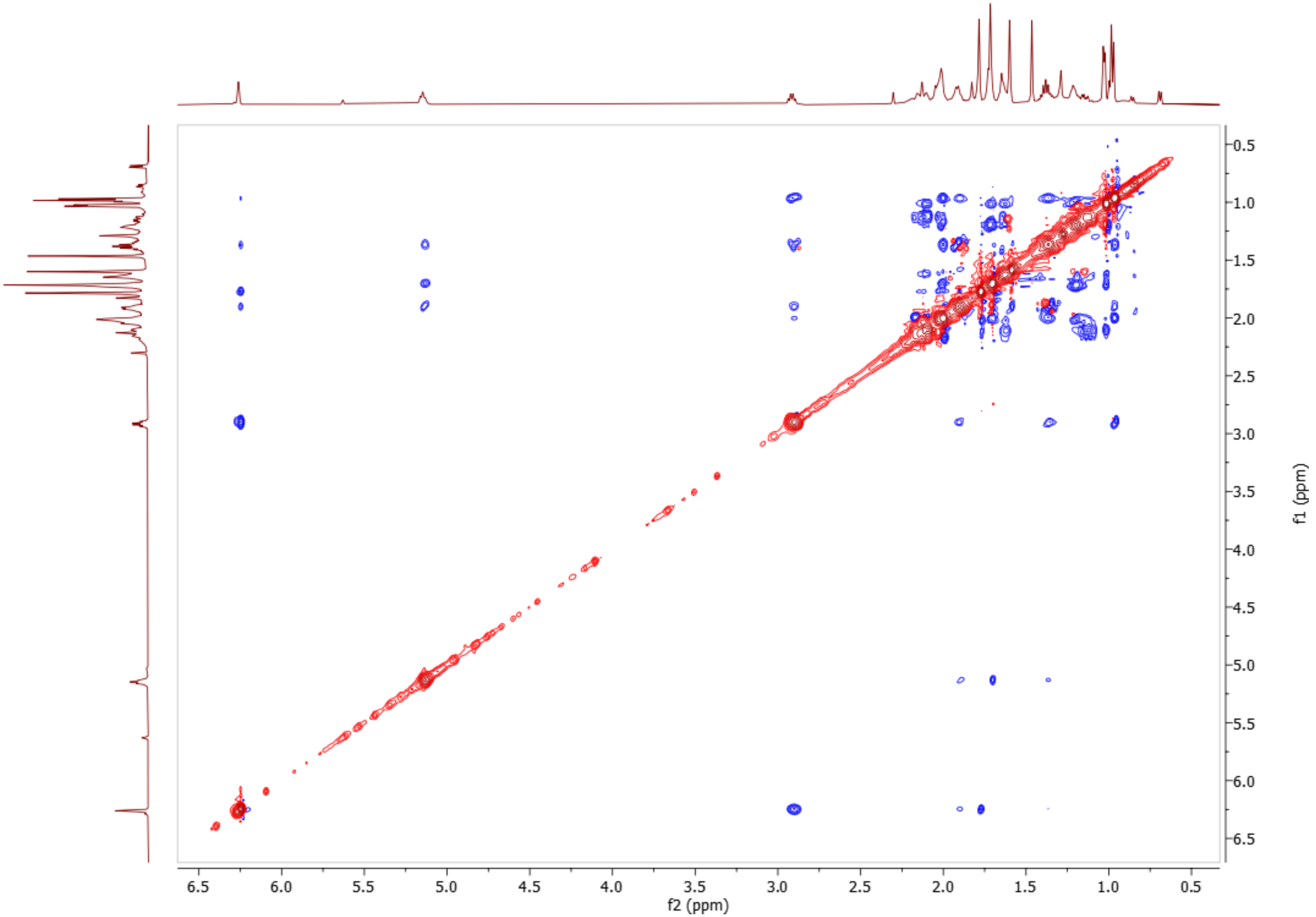

^{1}H-^{1}H COSY NMR spectrum of isoelisabethatriene C (**S1**) measured at 500 MHz in $CDCl_3$.
